# Ecology and Genetic Lineages of Nasal *Staphylococcus aureus* and MRSA Carriage in Healthy Persons with or without Animal-Related Occupational Risks of Colonization: A Review of Global Reports

**DOI:** 10.3390/pathogens10081000

**Published:** 2021-08-08

**Authors:** Idris Nasir Abdullahi, Carmen Lozano, Laura Ruiz-Ripa, Rosa Fernández-Fernández, Myriam Zarazaga, Carmen Torres

**Affiliations:** Area of Biochemistry and Molecular Biology, University of La Rioja, Madre de Dios 53, 26006 Logroño, Spain; idris-nasir.abdullahi@unirioja.es (I.N.A.); carmen.lozano@unirioja.es (C.L.); laura_ruiz_10@hotmail.com (L.R.-R.); rosa.fernandez.1995@gmail.com (R.F.-F.); myriam.zarazaga@unirioja.es (M.Z.)

**Keywords:** *Staphylococcus aureus*, MRSA, nasal colonization, genetic lineages, CC398, livestock, *S. pseudintermedius*, CoNS

## Abstract

In this conceptual review, we thoroughly searched for appropriate English articles on nasal staphylococci carriage among healthy people with no reported risk of colonization (Group A), food handlers (Group B), veterinarians (Group C), and livestock farmers (Group D) published between 2000 and 2021. Random-effects analyses of proportions were performed to determine the pooled prevalence of *S. aureus*, MRSA, MRSA-CC398, and MSSA-CC398, as well as the prevalence of PVL-positive *S. aureus* from all eligible studies. A total of 166 eligible papers were evaluated for Groups A/B/C/D (n = 58/31/26/51). The pooled prevalence of *S. aureus* and MRSA in healthy humans of Groups A to D were 15.9, 7.8, 34.9, and 27.1%, and 0.8, 0.9, 8.6, and 13.5%, respectively. The pooled prevalence of MRSA-CC398 nasal carriage among healthy humans was as follows: Group A/B (<0.05%), Group C (1.4%), Group D (5.4%); and the following among Group D: pig farmers (8.4%) and dairy farmers (4.7%). The pooled prevalence of CC398 lineage among the MSSA and MRSA isolates from studies of the four groups were Group A (2.9 and 6.9%), B (1.5 and 0.0%), C (47.6% in MRSA), and D (11.5 and 58.8%). Moreover, MSSA-CC398 isolates of Groups A and B were mostly of *spa*-t571 (animal-independent clade), while those of Groups C and D were *spa*-t011 and t034. The MRSA-CC398 was predominately of t011 and t034 in all the groups (with few other *spa-*types, livestock-associated clades). The pooled prevalence of MSSA and MRSA isolates carrying the PVL encoding genes were 11.5 and 9.6% (ranges: 0.0–76.9 and 0.0–28.6%), respectively. Moreover, one PVL-positive MSSA-t011-CC398 isolate was detected in Group A. Contact with livestock and veterinary practice seems to increase the risk of carrying MRSA-CC398, but not in food handlers. Thus, this emphasizes the need for integrated molecular epidemiology of zoonotic staphylococci.

## 1. Introduction

Many coagulase-positive and negative staphylococci are normal microbiota of the nasal cavity. However, some of them are of great public health importance due to their capacity to produce staphylococcal infections and diseases in humans and animals, and being responsible for zoonosis [1,2].

The main reservoir site for *staphylococcal* nasal carriage is the anterior nares and vestibules [3]. *Staphylococcus aureus* (*S. aureus*) is the most important nasal staphylococci and has been found in about 30% of healthy adults [3]. It was estimated that previous nasal colonization in 30% of the cases of bacteremia was due to *S. aureus* [4]. Essentially, *S. aureus* is an important cause of community-acquired (CA) and hospital-acquired (HA) human infections, and a potential strategy for controlling these infections is by eliminating or minimizing nasal carriage, such as those with intermittent and persistent carriage of the same or different *S. aureus* strains [5]. A more worrisome dimension is the isolation of nasal *S. aureus* in vulnerable groups such as the immunocompromised and immunosuppressed [6]. For instance, a study by Leshem et al. [7] reported identical *S. aureus* strains in 80% of infants and their mothers. Furthermore, 90% of these newborns contracted *S. aureus* from their maternal nasal strains [7].

The transmission of CA- and HA-methicillin-resistant *S. aureus* (MRSA) has increased the challenge of infection control. Since the first case was reported in 1961 in the United Kingdom [8], MRSA has been recognized to be most often associated with various infections in patients exposed to nosocomial settings, which is known as HA-MRSA. The advent of CA-MRSA gave rise to a substantial change in the epidemiology of MRSA isolates that were observed during the past few decades. The increasing number of infections caused by CA-MRSA in community settings has attracted much attention from scientists worldwide [8]. Although CA-MRSA is often defined by an absence of risk factors for HA-MRSA infections, it has also been differentiated through the possession of unique antimicrobial resistance patterns and molecular characteristics [9]. Generally, HA-MRSA typically harbors SCC*mec* I, II, and III, while CA-MRSA carries SCC*mec* IV or V [10]. It appears that CA-MRSA is less resistant to antibiotics than HA-MRSA [11]. Additionally, CA-MRSA isolates often carry the *lukSF-PV* genes that code for Panton–Valentine leukocidin (PVL), a cytolytic and toxic substance that has tropism to neutrophils [12]. Moreover, it is well established that *S. aureus* is notorious for its ability to produce a series of virulence factors. For instance, *S. aureus* causes food poisoning due to its ability to produce enterotoxins. Thus, food handlers carrying enterotoxin-producing *S. aureus* can contaminate food, thus leading to food poisoning. Consequently, food handlers may constitute a reservoir of virulent strains of *S. aureus* in their nose and may be vehicles of their transmission into food due to uncontrolled hand-to-nose activities [13].

Aside from HA- and CA-MRSA infections, the emergence of livestock-associated (LA)-MRSA, especially of the lineage MRSA-CC398, has also caused alarming rates of staphylococcal colonization and infection among humans in contact with livestock, mostly with pigs, suggesting an increased risk of zoonotic transmission [14]. This MRSA-CC398 lineage could, subsequently, be dispersed to the environment and to other species of animals through the food chain and direct contact [15]. In this sense, a recent study performed in Spain demonstrated a significant association between the rate of MRSA-CC398 at hospital level and the density of pig farming in the surrounding regions [16]. Nevertheless, this association seems not to be when MSSA-CC398 isolates are considered [17,18].

Occupational contact with animals who had persistent nasal *S. aureus* colonization or contaminated animal products such as meat, milk, and cheese could result in contracting *S. aureus* by healthy humans [19]. In certain circumstances, veterinarians and farmers could serve as a source of transmission of *S. aureus* to previously non-colonized animals (reverse zoonosis) [20]. Moreover, food handlers can also serve as another vehicle for the transmission of pathogenic staphylococci via nasal discharge [21].

Moreover, veterinarians and veterinary students who frequently encounter sick and healthy animals are at risk of contracting certain staphylococci predominantly found in animals. Hence, studying the nasal ecology of veterinarians and veterinary students may provide greater insight into through zoonotic transmission of staphylococci-microbiota in animals. In this regard, it is important to remark that *Staphylococcus pseudintermedius* (*S. pseudintermedius*), a common colonizer of companion animals (particularly dogs) is frequently associated with skin and soft tissue infections (SSTI) in these animals [22]. Similarly, SSTIs occur more frequently in humans who have close contact with dogs with active *S. pseudintermedius* infections [22]. Moreover, there are cases of human infection by *S. pseudintermedius* in persons that have dogs with similar bacteria [23].

Staphylococci are commonly comprised of multiple genetic lineages that have distinctive phenotypic and genotypic properties. It is important to investigate the genetic diversity (population structure) of staphylococcal strains that colonize the nasal cavities to understand how commensal strains present in healthy human might act as a predisposing (or preventing) factor for future invasive infections [24].

Coagulase-negative staphylococci (CoNS) and their antimicrobial resistance genes have been well described in many studies. Although, the role of CoNS in staphylococcal diseases is not completely understood because the bacteria that colonize the nasal cavity under physiological conditions have been interpreted as contamination in most microbiological analysis. Moreover, CoNS have an important role in the pathogenesis of laryngological infection due to their virulence factors such as enzymes, toxins, and biofilm formation [25].

There is evidence that *S. epidermidis* isolated from infections are a subset of those found on the skin’s surface and the nasal cavity [26]. This implies that certain lineages or specific virulence factors are associated with the emergence of *S. epidermidis* from a background of harmless ancestors [26]. For instance, the pathogenesis of *S. epidermidis* is associated with antibiotic resistance (such as those due to *mecA*), virulence genes (such as polysaccharide intercellular proteins) that enable attachment to host tissues and biofilms [27].

It is worthy to note that some CoNS such as *S. haemolyticus* or *S. lugdunensis* have been implicated in laryngological infections and inflammations related to tissues found in skull and neck bones, such as rhinosinusitis, necrotizing sinusitis, nasal polyps, nares, and nasal skin infections, periprosthetic joint infections, osteomyelitis, pharyngitis, and tonsillitis [25,27]. Hence, the molecular epidemiology of CoNS nasal carriage deserves to be studied in detail.

There have been many reports on the incidence or prevalence of nasal staphylococci carriage from single studies, single population or study group, cities, or individual countries but with a paucity of countries-wise or global systematic reviews or meta-analyses in healthy people. For instance, Awulachew et al. [28] systematically and selectively analyzed the *S. aureus* nasopharyngeal prevalence and their antimicrobial resistance phenotypes in healthy people. Of which, they reported a pooled global prevalence of *S. aureus* nasopharyngeal human carriage of 22%. Moreover, the pooled prevalence from Europe was slightly higher (25%) than the global prevalence [28]. It is worthy to note that the study by Awulachew et al. [28] and many others focused solely on either *S. aureus*, MRSA, CoNS, or healthy people without categorizing specific risk groups with nasal staphylococci colonization.

The characterization of nasal staphylococci provides a good model for the understanding of the molecular ecology of antimicrobial resistance and virulence genes of human health importance. Hence, this study aims to conceptually review appropriate and eligible data on the nasal staphylococcal community (with a focus on their prevalence), antimicrobial resistance, virulence genes, and genetic lineages in healthy humans with or without occupational risk of nasal staphylococci carriage related to food-producing animals.

## 2. Methodology

### 2.1. Study Design

This is a conceptual review of the distribution pattern and prevalence of the staphylococci community, their genetic lineages, antimicrobial resistance phenotypes and genotypes, and virulence genes in the nasal cavity of healthy humans, that was conducted using the best available evidence from global reports. Studies were classified based on whether they were on *S. aureus* (SA), MRSA, coagulase-positive staphylococci (CoPS), CoNS, or CoNS and CoPS. In this article, special focus was given to *S. aureus*-CC398 and -ST9, and *mecA*-mediated and *lukSF-PV*-producing *S. aureus* nasal isolates.

### 2.2. Articles Search

A thorough and comprehensive review of suitable and eligible full-text articles was conducted from ‘PubMed’, ‘Scopus’, ‘Hinari’, ‘Google Scholar’, and ‘Web of Science’ from 18 January to 11 April 2021 on peer-reviewed articles published between 2000 and 2021 on staphylococcal nasal carriages in healthy humans. This study identified 4702 records; after the elimination of duplicates, 615 remained. After screening titles and abstracts, we retained and assessed 321 full-text articles for eligibility.

### 2.3. Selection of Studies

Studies identified in the literature search were checked by title and abstract. The papers with relevant abstracts were examined in detail. The criteria for the inclusion and exclusion of the studies were established by the investigators before the literature was reviewed. The inclusion criteria were as follows: (1) studies that were original articles, short communications, correspondence, or letters that provided sufficient original data about the prevalence of ‘*Staphylococcus aureus* nasal carriage’, ‘MRSA nasal carriage’, ‘Coagulase-negative staphylococci’, ‘Coagulase positive staphylococci’, ‘Methicillin-resistant *Staphylococcus’*, ‘non-*aureus* staphylococcal nasal carriage’, ‘MRSP nasal carriage’, ‘Nasal staphylococci in veterinarians’, ‘Nasal staphylococci in veterinary students’, ‘Nasal staphylococci in livestock farmers’, ‘Nasal staphylococci in food handlers’, and ‘Nasal staphylococci in healthy people’; (2) studies in which all MRSA strains were adequately presented; and (3) studies that were published in English. The exclusion criteria were: (1) studies that contained duplicate data or were overlapping articles; (2) reviews and conference abstracts; (3) articles that included fewer than 10 subjects; (4) studies performed on healthy humans working in the hospitals or attending health centers/nursing homes and prison, or prison inmates or human healthcare students, or specific groups of populations that their social or living conditions could affect the nasal staphylococci status (homeless and athletes, among others); (5) articles that were published before the year 2000, (6) longitudinal studies with no specific nasal staphylococci prevalence at the initial sampling, and (7) studies that were solely on staphylococci isolates with no or full information of the number of persons and occupation they were isolated from.

### 2.4. Category of Studied People

The following four groups of healthy individuals were tested in this study:

Group A: Healthy people with no known risk of colonization.

Group B: Food handlers.

Group C: Veterinarians and veterinary students.

Group D: Livestock farmers.

### 2.5. Data Extraction

Authors independently ascertained the characteristics of each study, including the first author’s surname, year of publication, continent, country, study years, detection method, staphylococci prevalence, antimicrobial resistance phenotypes and genotypes, virulence factors, and molecular typing reports. When there was disagreement, the relevant paper was reviewed, and the differences were resolved by consensus. Finally, 166 full texts were included because they were the only available articles that directly focused on the distribution pattern of the staphylococci community, genetic lineages, antimicrobial resistance phenotypes and genotypes, and/or virulence genes in nasal cavities of healthy humans.

### 2.6. Statistical Analysis

The pooled prevalence of nasal carriage of *S. aureus*, MRSA or other non-*aureus* staphylococci was calculated. Medcalc software Version 2019.19.0.7 (Ostend, Belgium) was used for all statistical analysis. Where possible, an analysis of pooled prevalence was carried out using the random-effects model. Moreover, the pooled rate of nasal carriage by CC398 *S. aureus* isolates (MRSA or MSSA) was calculated, using the articles in which molecular typing of isolates were performed.

## 3. Main Findings and Discussion

### 3.1. Eligible Study Characteristics

**Figure 1:**
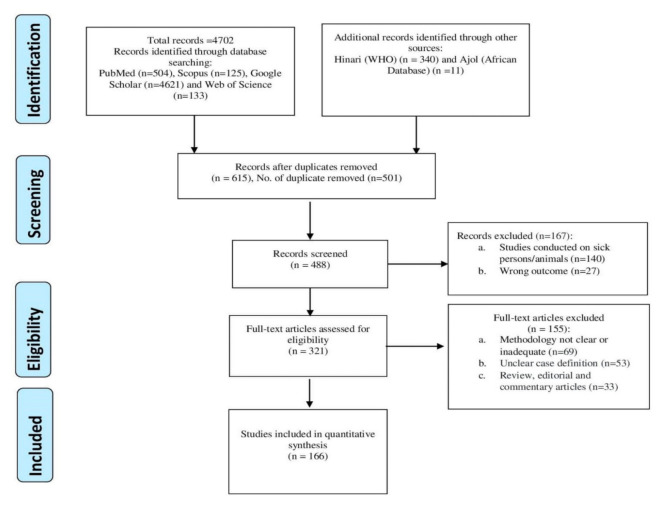
Identification and selection flowchart of articles on nasal staphylococci carriage in healthy human.

Data were extracted and synthesized from 166 articles after applying exclusion criteria (Figure 1). There were 58 studies on healthy people without a known risk of *S. aureus* nasal carriage (Group A), while 31, 26, and 51 were on food handlers, veterinarians/veterinary students, and livestock farmers (Groups B–D), respectively (Table 1, Supplementary Tables S1–S4). Out of the 58 studies on healthy humans of Group A, 51, 4, and 3 had cross-sectional, prospective, and cohort study designs, respectively. Among the eligible studies on food handlers, only one of them was a retrospective study, whereas five out of the studies on Group C were prospective studies. Five of the studies on livestock farmers had a prospective study design, whereas the others were cross-sectional studies (Supplementary Tables S1–S4).

Out of the studies on Group A, 12, 18, 15, 12, and 1 were from Africa, Asia, Europe, America, and Australia, respectively. Out of the studies on Group B, 8, 12, 7, and 4 were from Africa, Asia, Europe, and America, respectively. Of the studies in Group C, 3, 4, 12, 6, and 1 were from Africa, Asia, Europe, America, and Australia, respectively. Additionally, 10, 18, 18, and 5 of the studies in Group D were from Africa, Asia, Europe, and America, respectively (Supplementary Tables S1–S4) [13,20,23,29,30,31,32,33,34,35,36,37,38,39,40,41,42,43,44,45,46,47,48,49,50,51,52,53,54,55,56,57,58,59,60,61,62,63,64,65,66,67,68,69,70,71,72,73,74,75,76,77,78,79,80,81,82,83,84,85,86,87,88,89,90,91,92,93,94,95,96,97,98,99,100,101,102,103,104,105,106,107,108,109,110,111,112,113,114,115,116,117,118,119,120,121,122,123,124,125,126,127,128,129,130,131,132,133,134,135,136,137,138,139,140,141,142,143,144,145,146,147,148,149,150,151,152,153,154,155,156,157,158,159,160,161,162,163,164,165,166,167,168,169,170,171,172,173,174,175,176,177,178,179,180,181,182,183,184,185,186,187,188,189,190,191].

### 3.2. Prevalence of Nasal S. aureus and MRSA in Healthy Humans without Known Risk of Carriage (Group A)

Among all the studies on Group A (Supplementary Table S1), 53 were solely on *S. aureus* nasal carriage [20,29,30,31,32,33,34,35,36,37,38,39,40,41,42,43,44,45,46,47,48,49,50,51,52,53,54,55,56,57,58,59,60,61,62,63,64,65,66,67,68,69,70,71,72,73,74,75,76,77,78,79,80], 4 were on both CoNS and CoPS [81,82,83,84], whereas one was solely on CoNS [85]. Of all the eligible studies conducted from 2000 to 2021 (Supplementary Table S1), the calculated pooled prevalence of *S. aureus* and MRSA nasal carriage among healthy people was 15.9 and 0.8%, respectively, while the prevalence range was 2.3–79.6 and 0.0–17.5%, respectively (Table 1).

**Figure 2:**
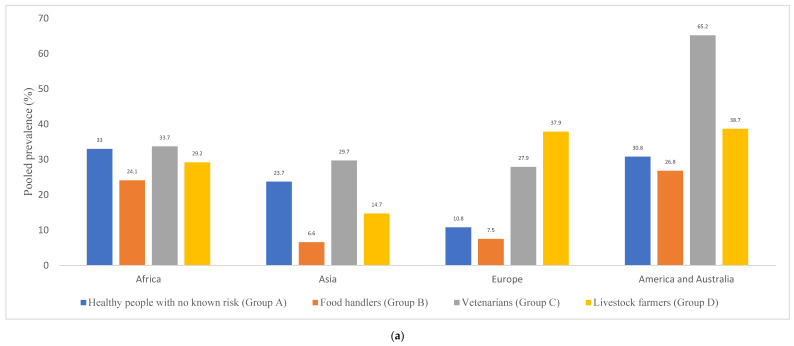

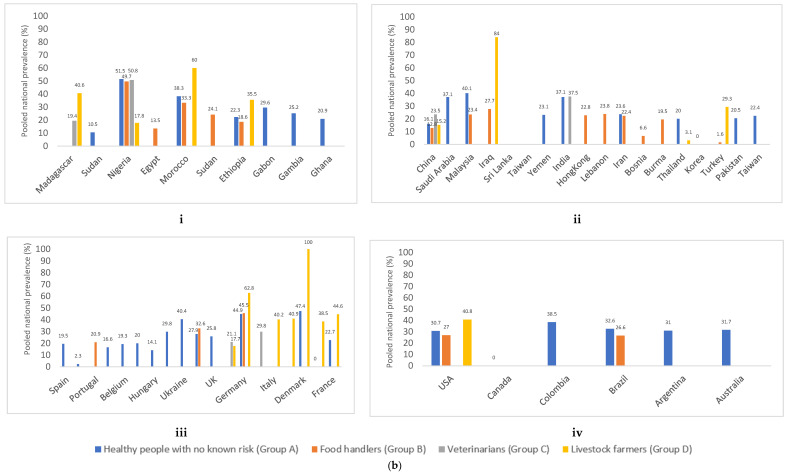
Pooled prevalence of *S. aureus* nasal carriage among healthy people with or without occupational risks of colonization (Groups A–D) by continent (**a**) and grouped by countries (**b**): (**i**) Africa; (**ii**) Asia; (**iii**) Europe; (**iv**) America and Australia. The number of studies per continent in Groups A, B, C, and D, respectively, were as follows: Africa (13, 10, 2, and 7); Asia (16, 11, 2, 5); Europe (22, 7, 2, and 9); America and Australia (12, 3, 1, and 5).

Figure 2 shows the pooled rates of *S. aureus* nasal carriage detected in studies performed in countries of different continents. Of which, the highest pooled prevalence of nasal carriage of *S. aureus* in healthy humans of Group A was obtained from African countries (33.0%, with a range of 10.5–51.5%), followed by countries of the Americas (30.8%, range 30.7–38.5%), countries of Asia (23.7%, range 16.1–40.3%), and the lowest pooled nasal carriage rate by the countries of Europe (10.7%, range 2.3–47.4%) (Figure 2a,b). Different variables could be responsible for the nasal *S. aureus* carriage variation among the continents and studies (such as the methodologies used, specific characteristics of the countries or of the individuals tested, or a pattern of antimicrobial use, among others). For instance, studies among Dutch children revealed a decreasing carriage rate during the first year of life, remaining stable at 20–30% until it increases again to 40–50% between the age of 6 to 12 years [192,193]. Moreover, in West Africa, these rates might be considerably different due to co-colonization with other pathogens or living conditions, such as large family sizes and lower sanitary standards, which are all associated with higher *S. aureus* nasal carriage [194,195].

**Figure 3:**
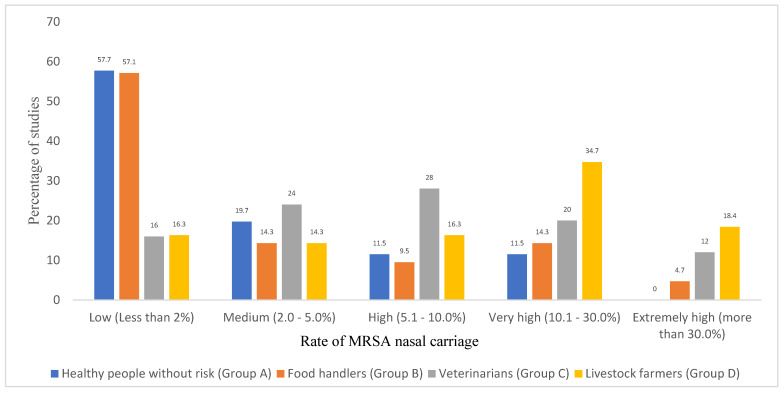
Percentage of eligible studies carried out on healthy humans of Groups A–D showing different rates of MRSA nasal carriage. NB—The number of eligible studies on nasal MRSA carriage per Groups A, B, C, and D were 52, 21, 25, and 49, respectively (included in Table 2).

The rate of MRSA carriage in the healthy people of Group A was low (less than 2%) in most of the reviewed studies (n = 30/52, 57.7%) that were performed in 24 countries of all continents (Figure 3). A medium MRSA prevalence (2–5%) was reported in 10 studies (19.2%) performed in Colombia, Spain, Morocco, India, Ukraine, Mexico, Argentina, and Ethiopia. Moreover, a high prevalence (5.1–10.0%) was identified in six studies (11.5%) performed in Spain, Iran, Nigeria, and Brazil. Finally, a very high MRSA prevalence (10.1–20.0%) was identified in six studies (11.5%) performed in Nigeria, Malaysia, Iraq, and Iran (Table 2). It appears that most studies with a very high MRSA prevalence were from developing countries (with few exceptions) and might be due to uncontrolled use or misuse of antimicrobial drugs in most of these countries [196].

**Figure 4:**
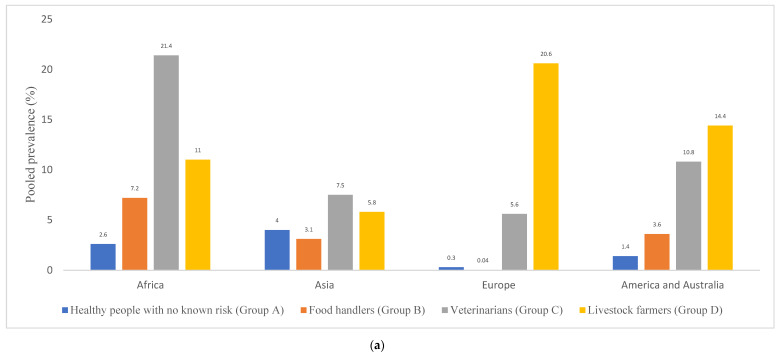

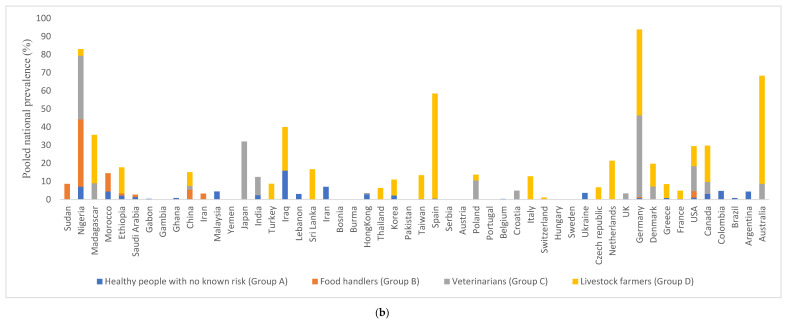
Pooled prevalence of MRSA nasal carriage among healthy people with or without occupational risks of colonization (Groups A-D) by continent (**a**) and grouped by countries/continents (**b**). In each country, the indicated pooled prevalence in each group (A–D) was analyzed in an independent way. The number of studies per continent in Groups A, B, C, and D, respectively, were as follows: Africa (10, 5, 3, and 8); Asia (17, 9, 4, 11); Europe (12, 6, 13, and 24); America and Australia (13, 1, 5, and 6).

Figure 4a,b showed the pooled prevalence of MRSA nasal carriages by countries and continents in healthy people of Group A. Of which, Iraq, Nigeria, Iran, Morocco, and Colombia had the highest pooled prevalence of MRSA. This also reflected on the pooled prevalence of nasal MRSA carriage by continent. Of which, the highest was obtained from Asia (4.0%), followed by Africa (2.6%), America/Australia (1.4%), and least in Europe (0.3%) (Figure 4a). The high prevalence reported in these countries and continents could be due, in part, to differences in the laboratory protocol and media used for the identification of isolation and the identification of MRSA. In most studies from the developing countries, MRSA detection were mainly based on phenotypic detection (Supplementary Table S1).

### 3.3. Prevalence of Nasal S. aureus and MRSA Carriage among Food Handlers (Group B)

The LA-MRSA is widely disseminated as a nasal colonizer of conventionally raised livestock and humans subjected to occupational exposure. Reports on the contamination of raw meat, milk, and other animal products raise the question as to whether occupationally exposed food handlers are at particular risk of nasal colonization by LA-MRSA. Nasal carriage of *S. aureus*, particularly those producing enterotoxins, constitutes the main risk of contamination in food, followed by possible food poisoning. Therefore, the investigation of *S. aureus* carriage among food handlers and the analysis of the prevalence of toxin genes in colonizing strains is important to prevent food contamination with toxigenic strains that may be related to food poisoning or other diseases [21]. Very recently, the relevance of the superantigen SEIW (encoded by *selw* gene) on *S. aureus* pathogenesis has been postulated [197]. The SEIW has been shown to be highly prevalent among *S. aureus* isolates, and it could be a unique superantigen expressed by those of the CC398 lineage [197]. Hence, *S. aureus* acquisition by handling contaminated milk, meat, and meat products should be considered.

According to our analysis, a relatively low pooled rate of nasal MRSA carriage in food handlers among eligible studies was found: 0.9% from 21 eligible studies with a range of 0.0–37.1%, whereas the pooled rate of *S. aureus* nasal carriage was 7.8% with a range of 1.4–60% (Table 1).

In relation to the pooled nasal *S. aureus* carriage of healthy humans of Group B by continents, the highest values were detected in the Americas and Africa (26.8 and 24.1%, respectively), with lower values in countries of Europe (7.5%) and Asia (6.6%) (Figure 2a). In African countries, Nigeria had the highest pooled prevalence (49.7%) (Figure 2b(i)) [85,86,87]. Moreover, among European countries, a study in Germany reported the highest *S. aureus* prevalence of 45.4% among butchers, meat sellers, and cooks [110]; the least prevalence was reported from Bosnia (1.4%) [106].

If we consider the studies included in Table 2 in relation to the MRSA nasal carriage of Group B, 57.1% of them (n = 12) reported a low rate of colonization (<2%), 14.3% of the studies reported a medium rate of colonization (2–5%), 9.5% of the studies reported a high rate of colonization (5–10%), while 9.5% of the studies reported a very high rate (10–20%), and 8.5% an extremely high rate (20–40%) (Figure 3).

The overall low prevalence of nasal MRSA carriage from food handlers (0.9%, similar to that from Group A) may be associated with the lack of direct contact with live animals (but with their food products), although it could also be affected by the adoption of preventive measures of personal hygiene and environmental sanitation, such as consistent hand washing, the use of face mask, and consistent hand glove practice.

### 3.4. Prevalence of Nasal S. aureus and MRSA Carriage among Veterinarians and Veterinary Students (Group C)

Cases of colonization or infection caused by MRSA are frequently reported in people who work with animals, including veterinary personnel and students. Most of the eligible studies on veterinarians included in this review focused on MRSA, probably because of possibility of zoonotic acquisition.

According to our results, the pooled prevalence of *S. aureus* nasal carriage in veterinary personnel (Group C) was 34.9% (range 19.4–50.8%), whereas the corresponding of MRSA was 8.6% (range 0.7–38.4%) (Table 1). A study carried out by Anueyiagu et al. [20] in Nigeria reported the highest prevalence of MRSA in veterinary students (38.4%) and least in a study from Hong Kong [141] that reported 0.7% from veterinary personnel.

With respect to the *S. aureus* nasal carriage prevalence by continents, a similar pooled prevalence was obtained in individuals of Group C in countries of Europe, Asia, and Africa (27.9–33.7%). However, a high prevalence was detected in one unique study included in the America continent [131] (Figure 2).

Of the 25 studies in which the prevalence of MRSA among veterinary personnel has been evaluated, 16.0% of them reported a low rate of nasal carriage (<2%), 24.0% of the studies indicated a medium rate (2–5%), 28.0% of the studies a high rate of carriage (5.1–10%), 20.0% a very high rate (10.1–30%), and 12.0% reported an extremely high rate of nasal carriage (30–50%) (Table 2, Figure 3).

The higher prevalence of MRSA carriage in veterinary personnel has been proven by multiple studies all over the world. The rates in Europe vary from 0.7–44.9% [127,128]. Traditionally, high prevalence data come from countries with well-developed livestock production, such as the Netherlands, Denmark, or Germany [124,128,130,198] (Table 2). The type of veterinary practice, frequency of contact with animals, time since exposure, and the study design could be factors that led to international differences in prevalence rates.

### 3.5. Prevalence of Nasal S. aureus and MRSA Carriage among Livestock Farmers (Group D)

According to our results, the pooled prevalence of *S. aureus* and MRSA nasal carriage among livestock workers was 27.1 and 13.5%, respectively. Moreover, the range of *S. aureus* and MRSA nasal carriages were 3.1–62.8 and 0.0–85.8%, respectively (Table 1; Supplementary Table S4). This category of the population had the highest pooled MRSA nasal carriage prevalence.

In relation to the pooled prevalence of *S. aureus* nasal carriage among farmers by continents *(*Figure 2a), a higher rate was obtained from Europe and America (37.9 and 38.7%, respectively), with lower values in Africa and Asia (1.7 and 29.2%, respectively). In Europe, Germany and France reported the highest prevalence of 62.8 and 44.6%, respectively. However, a small sample size (n = 16) study conducted in Denmark reported a 100% *S. aureus* nasal carriage among livestock workers [170] (Figure 2b(iii)). In Africa, Morocco and Madagascar had pooled a *S. aureus* prevalence of 60.0 and 40.6% (Figure 2b(i)). Additionally, the USA, Argentina, Malaysia, Colombia, and Brazil had pooled *S. aureus* prevalence in the range 30–41% (Figure 2b(iv)).

If we consider the studies included in Table 2 in relation to the MRSA nasal carriage of Group D, only 16.3% of the 49 studies of this group reported a low MRSA nasal carriage (<2%), 14.3% of the studies reported a medium rate of colonization (2–5%), 16.3% of the studies reported a high rate (5.1–10%), while 34.7 and 14.3% of the studies reported very high (10.1–30%) and extremely high (30.1–>50%) nasal carriage rates, respectively (Figure 3). The calculated pooled prevalence of nasal MRSA carriage among livestock farmers by continent was highest in Europe (20.6%), followed by America/Australia (14.4%), Africa (11.0%), and least in Asia (5.8%) (Figure 4a).

In relation to MRSA in African countries, Madagascar and Ethiopia had the highest pooled MRSA prevalence in livestock farmers (26.5 and 14.2%, respectively) [141,143], while Nigeria and Morocco had the lowest [142,145] (Figure 4b). Moreover, specific studies in Spain and Germany on pig farmers reported a very high MRSA prevalence of 57.9% and 84.7%/85.8%, respectively [128,171,181]; these studies were performed in regions with a very high density of pig farming in both countries. Other European studies reported a lower prevalence of MRSA in pig farmers, and even a study in Switzerland did not detect any pig farmer with LA-MRSA nasal colonization [122].

**Figure 5:**
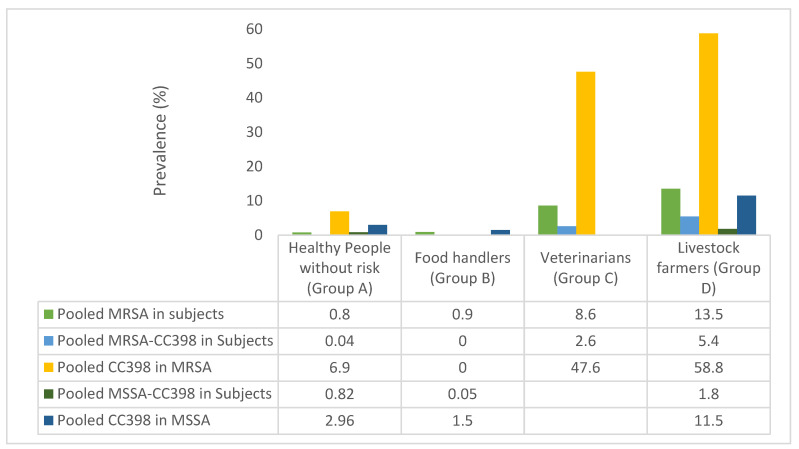
Pooled rates of MRSA, MRSA-CC398, and MSSA-CC398 nasal carriage in the healthy individuals of the Groups A–D, and rate of CC398 lineage in relation to MRSA and MSSA isolates. NB-a—Pooled MRSA rates were obtained from the Supplementary Tables S1–S4 and pooled MRSA-CC398 and MSSA-CC398 data of Table S7; b—No study reported MRSA-CC398 lineage in food handlers; c—None of analyzed studies carried out molecular typing of nasal MSSA in Group C; d—There were only two studies (of 13 eligible) that identified MRSA-CC398 in healthy people without risk of nasal colonization; e—There were two studies (of 5 eligible) that reported MSSA-CC398 in food handlers.

**Figure 6:**
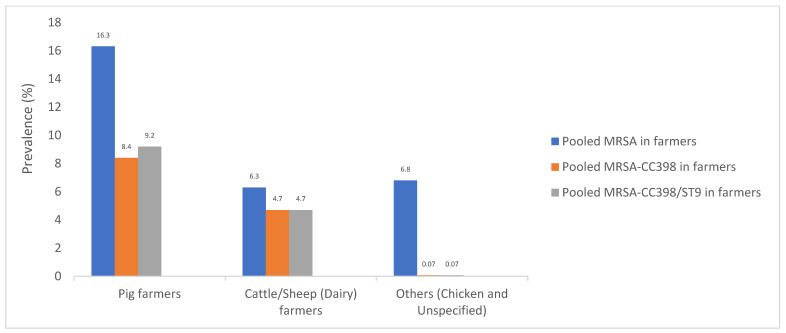
Pooled rates of MRSA, MRSA-CC398, and MRSA-CC398/ST9 nasal carriage in livestock farmers (group D). NB-a—Pooled MRSA rates in farmers were obtained from Supplementary Table S4 and pooled MRSA-CC398 and MSSA-CC398 from Supplementary Table S7; b—There were 19 and 5 studies in which we performed molecular typing of MRSA in pig and dairy farmers, respectively; c—There were 4 molecular typing studies classified as others (chicken and unspecified/mixed type of farmers).

**Figure 7:**
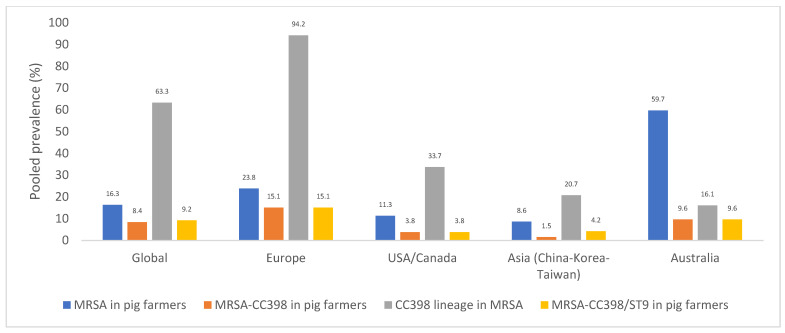
Pooled rates of MRSA, MRSA-CC398, and MRSA-CC398/ST9 nasal carriage in pig farmers depending on the continent of origin, and rate of CC398 lineage in relation to MRSA isolates. NB—Only one study from Australia on 52 pig farmers was included in this analysis.

A pooled global prevalence of MRSA in all livestock farmers was 13.5% (Figure 5); Nevertheless, differences were observed when the type of livestock animals were taken into account. In this respect, a pooled MRSA prevalence of 16.3% was obtained when only pig farmers were considered (Figure 6). Of which, the following differences in the prevalence of MRSA in pig farmers by continents were noted: Europe (23.9%), Asia (8.6%) (China, Korea, Thailand, and Taiwan), and the USA/Canada (11.3%) (Figure 7). In dairy farmers, a pooled prevalence of 6.3% MRSA nasal carriage was obtained (Figure 6). It is important to remark that a single study reported a very high MRSA rate (32.9%) isolated from the Netherlands in dairy workers [180]. This must have affected the net pooled prevalence in this category of farmers. Similarly, a single study in Australia reported a very high prevalence (59.6%) of MRSA in pig farmers [185]. Moreover, a 6.8% pooled prevalence of MRSA was calculated from 10 other studies that comprised of poultry and unspecified/mixed-type farmers (Figure 6).

### 3.6. Antibiotic Resistance Identified in Staphylococci of Nasal Cavities of Healthy Humans

Out of the 58 studies on healthy humans of Group A, only 47 reported antimicrobial resistance phenotypes, while 40 detected the antimicrobial resistance genes profile of the staphylococci isolates (Supplementary Table S1). Some of these studies individually reported the *mecA* gene from *S. aureus* isolated from nasal samples (Supplementary Table S1). The *mec*A gene was the unique gene associated to methicillin resistance in all eligible studies and none of them found the *mec*C gene. Aside from *S. aureus* isolates, *mecA* was identified in some *S. epidermidis* isolates among healthy veterinary students in Greece [140].

Aside from the gene *mecA* for methicillin resistance in nasal *S. aureus* isolates, others such as *erm*(A), *erm*(C), *msr*(A), and *erm*(T) genes were reported by Lozano et al. [67], while *erm*(C) and *erm*(A) were detected by Kock et al. [175]. Furthermore, *tet*(M)*, tet*(L), *aacA, aphD, dfrK, erm*(C), and *erm*(T) were reported by Paterson et al. [136]. The *erm*(T) gene was detected in MSSA and MRSA of lineage CC398 [67,136]. Other antimicrobial resistance genes were identified in both CoPS and CoNS (Supplementary Tables S1–S4). As the present review focused on the *S. aureus*-CC398 lineage, it is necessary to remark that the detection of tetracycline and erythromycin resistance genes (*tet*(M) and *erm*(T)) are often potential markers of livestock-association such as the MRSA-CC398 and MRSA-CC398/ST9 [17,155]. This suggests that tetracycline resistance may be useful in the determination of the epidemiological source of MRSA isolates.

### 3.7. Prevalence Pattern of Panton Valentine Leukocidin in Nasal Staphylococcus aureus in Healthy Humans

Diverse categories of virulence genes have been detected in nasal staphylococci of healthy humans (Supplementary Tables S1–S4). Staphylococcal virulence factors have been shown to contribute to the increased severity of associated infections. Particularly, the virulence factors that have been described in nasal *S. aureus* are the Panton–Valentine Leucocidin (PVL), staphylococcal enterotoxins, and toxic shock syndrome toxin [199,200,201,202,203].

In healthy humans, Osman et al. [97], Karapsias et al. [59], Mourabit et al. [38], El-Shenawy et al. [86], and Lozano et al. [67] studies individually identified *sea*, *sec*, and *sed, etb; lukF*/*S-PV, tst-1, eta, hla, hld, hlg-2, hlg, seb, sek, sep* genes from *S. aureus* nasal isolates. In this present review, the detection of the *lukF*/*S-PV* gene (encoding PVL) in nasal *S. aureus* isolates was given special attention due to its clinical and epidemiological significance.

Supplementary Table S5 shows the 18 studies (from 13 different countries) in which the genes encoding PVL have been analyzed in nasal *S. aureus* of healthy humans (most of them of Group A and only three of Groups B/D), indicating the prevalence in each of the studies as well as the associated *S. aureus* genetic lineages. The pooled prevalence of PVL-MSSA and PVL-MRSA were 11.5 and 9.6%, respectively (with ranges of 0.0–76.9 and 0.0–28.6%, respectively). If we consider the pooled prevalence in each of the countries, the highest prevalence of PVL-MSSA was detected in Ghana, with a pooled prevalence of 67.9% [33,34], whereas, in the case of PVL-MRSA, the highest prevalence of 28.6% was reported in Argentine children [79]. In China, Gong et al. [48] reported a very high prevalence of PVL-MSSA of 76.9% in healthy children, while Yan et al. [49] reported a very low prevalence of 2.3% in healthy adults. However, in the USA, a nasal PVL-MSSA prevalence of <5% was reported by Velasco et al. [69] and Wardyn et al. [66], but 29.0% by Wardyn et al. [73] in humans of Group A. Based on these findings, it can be inferred that PVL was more frequent in MSSA than in MRSA nasal isolates in most of the 18 eligible studies (Supplementary Table S5). Additionally, studies from Sub-Saharan Africa suggest that the region is MSSA-PVL endemic due to the very high prevalence especially reported [33,34].

The genetic lineages of PVL-MSSA isolates were reported in eight of the eligible studies, showing diverse clonal complexes, such as CC152, CC1, CC5, CC121, CC15, CC30 CC8, CC88, CC96, CC97, CC45, CC707, CC7, CC6, or CC22 (Supplementary Table S5). Interestingly, the PVL encoding genes were detected in a CC398-t011-MSSA isolate recovered from the nasal sample of a healthy human of Group A in China [49]. The production of PVL is very unusual among CC398 *S. aureus* isolates. For other MSSA-non-CC398 isolates, the predominant PVL-MSSA in the Ghanaian community was CC152-t355 [33,34]. In Nigeria, the lineages CC5-t311/t279/t18346 were reported from chicken and pig slaughterhouse workers [145]. Moreover, a PVL-positive/ST2250 nasal *S. argenteus* isolate that corresponded to CC2250 was identified by Aung et al. [115]. In relation to MRSA-PVL positive isolates, the lineages ST80-t044, ST5-t313, and ST59-t3527 were detected in three of the studies [59,79,157] (Supplementary Table S5).

### 3.8. Genetic Lineages in Nasal S. aureus of Healthy Humans of Groups A–D, with Special Focus on the CC398 Lineage

Fifty-two studies reported the molecular typing (staphylococcal protein-A (*spa*)*,* sequence types (ST) and/or clonal complexes (CC)) of *S. aureus* isolated from the nasal cavity of healthy humans of Groups A–D (Supplementary Table S6). In Figure 5, it is shown the pooled prevalence of MRSA in healthy humans of the groups A–D (obtained with data of Supplementary Tables S1–S4), and the pooled prevalence of MRSA-CC398 (in relation to healthy individuals and to typed MRSA isolates) and of MSSA-CC398 (in relation to healthy individuals and to typed MSSA isolates) in the four studied groups (obtained from data presented in Supplementary Table S6). The pooled prevalence of MRSA among humans of group A, B, C, and D was of 0.8%, 0.9%, 8.6%, and 13.5%, respectively.

The CC398 lineage was scarcely detected among healthy humans of Group A, with pooled prevalence of 0.04% (for MRSA-CC398) and 0.82% (for MSSA-CC398). Moreover, the lineage CC398 represented 2.9 and 6.9% of total MSSA and MRSA isolates of Group A, respectively (Figure 5). The MSSA-CC398 isolates were detected in 3 of the 12 studies in which *S. aureus* molecular typing was performed [49,67,72], and these studies were carried out in Spain, China, and the USA; the *spa*-type t571 was found in most of cases, although a few isolates with *spa*-types t034 and t1451 were also identified. In the studies of China and the USA, the CC398 isolates represented 8–10% of the total MSSA isolates. In relation to MRSA-CC398 in Group A, it was detected in only 3 of the 15 selected studies, performed in China and in different European countries [49,62,63]; these isolates were ascribed to *spa*-types t034, t011, and t108. The most frequent lineages detected in Group A in MSSA versus MRSA isolates, other than CC398, were as follows: CC15, C30, CC5, CC45, CC152, CC84 versus CC5, CC152, and CC88 (Supplementary Table S6).

The data obtained in the studies of Group B were very similar to the ones of Group A, with a very low pooled prevalence of MSSA-CC398 and MRSA-CC398 nasal carriages among food handlers (≤0.05%) (Figure 5). In this sense, only two studies performed in Hong Kong and Germany detected MSSA-CC398 (*spa*, t034, t571, and t1451) [82,88]; on the other hand, no MRSA-CC398 isolates were detected in the six selected studies of this group. Other lineages frequently detected in this group, in MSSA versus MRSA were as follows: CC22, CC15, and CC96 versus CC22 and CC6 (Supplementary Table S6).

The pooled rate of MRSA-CC398 nasal carriage among healthy veterinary personnel (Group C) was of 2.6%, representing 28.4% of total MRSA of this group (Figure 5). They were detected in 6 of the 11 studies in which molecular typing was performed, and they were carried out in Switzerland, the Czech Republic, and the UK [121,122,123,124,135,136]. Most of the isolates corresponded to the *spa* type t011, but in a few cases, t034 or t899 were also identified. The other lineages among MRSA isolates detected in Group C were CC22 and ST59 (Supplementary Table S6). However, molecular typing of MSSA was not performed in any of selected studies.

Finally, in Group D (healthy livestock farmers), of the 10 studies in which molecular typing was performed on MSSA, 9 of them detected MSSA-CC398 isolates (*spa* t034 and t011); these studies were performed in China, Switzerland, Germany, Poland, and the USA. On the other hand, of the 28 studies in which molecular typing was performed on MRSA, CC398 isolates were detected in 17 of them (mostly of *spa*-types t011 and t034; and to a lesser extent t899, t1451, and t1456) (Figure 5; Supplementary Table S6). The pooled rates of MRSA-CC398 and MSSA-CC398 nasal carriages among livestock farmers were of 5.4 and 1.8%, respectively (Figure 5). Moreover, MRSA-CC398 and MSSA-CC398 represented 58.8 and 11.5% of total MRSA and MSSA isolates recovered from livestock farmers (Figure 5).

The countries in which nasal MRSA-CC398 was most prevalent were Germany, Poland, the USA, Canada, Switzerland, the Netherlands, Australia, Korea, Spain, and Italy. However, in China and Taiwan, three studies detected ST9 in pig farmers, also considered as LA-MRSA, in some cases associated with *spa*-type t899 (Supplementary Table S6). Interestingly, the pooled prevalence of MSSA-CC398 nasal carriage in livestock farmers (1.8%) was higher than in individuals of Groups A/B (0.82%/0.05%), with associated *spa*-types t011, t034, and t108; moreover, the rate of CC398 among MSSA isolates was higher in Group D (11.8%) than in groups A/B (<3%) (Figure 5; Supplementary Table S6). Other lineages, in addition to CC398 detected in studies of Group D among MSSA versus MRSA were as follows: CC7 (China), CC45, CC15, and CC30 versus CC7 and CC9 (China and Thailand) (Supplementary Table S6).

The data of MRSA, MRSA-CC398, and MRSA-CC398/ST9 in relation with the type of livestock is shown in Figure 6. In this sense, pig farmers showed higher rates of MRSA (16.3%) and MRSA-CC398 nasal carriages (8.4%) than dairy farmers (6.3 and 4.7%, respectively). These rates were lower in the studies in which chicken farmers, or unspecified or mixed type of farmers were included (6.8 and 0.07%, respectively). Nevertheless, the lineage CC398 represented a high proportion of MRSA isolates in both pig and dairy farmers (63.3 and 75%, respectively). The rate of MRSA-CC398/ST9 (LA-MRSA) nasal carriage among pig farmers was 9.2% (Figure 6). The molecular surveillance of the evolution of *S. aureus*-CC398 lineage should not only be in pig farms but in other livestock farms. For instance, cows’ farms are considered the main animal reservoir for the emergence of human epidemic clones of *S. aureus,* according to a recent study that analyzed the gene exchange as the driver of the ecological success of this multi-host bacterial pathogen [204].

Differences were observed in the data of the pooled rates of MRSA, MRSA-CC398, and MRSA-CC398/ST9 nasal carriages of pig farmers when the different continents were compared (Figure 7). In this sense, the highest rate of MRSA-CC398 nasal rates was obtained in European countries (15.1%), especially in Germany, Switzerland, Italy, and Spain [121,161,172,174,181]; this lineage represented 94.2% of the MRSA isolates recovered from pig farmers. The most prevalent LA-MRSA lineage in Europe is ST398. This ST dominated most of the studies on humans with risk of animal contact due to occupation. Most animal-related MRSA strains belonged to *spa*-types t011, t034, and t1451, and typically showed resistance to tetracycline, an antibiotic frequently used in animal food production.

The rate of MRSA-CC398 nasal carriage among pig farmers in the studies carried out in the USA/Canada was of 3.8% and represented one/third of the MRSA isolates recovered in this population. Moreover, the rate of nasal carriage found in the studies performed in Asian countries (China, Korea, and Taiwan) was of 1.5%, although it increased to 4.2% when the ST9 lineage was also considered. The ST9 lineage is a relevant LA-MRSA among Asian countries.

The CC398 lineage has been reported from *S. aureus* isolates from various human populations and geographical locations. Based on the high rate of CC398-*S. aureus* reported from these studies, measures for the prevention of infections in humans by MRSA CC398 should focus on humans with direct exposure to livestock, especially with pigs. Regimens for screening and admission to hospitals should include farmers raising productive livestock and veterinarians with subsequent precaution measures.

**Figure 8:**
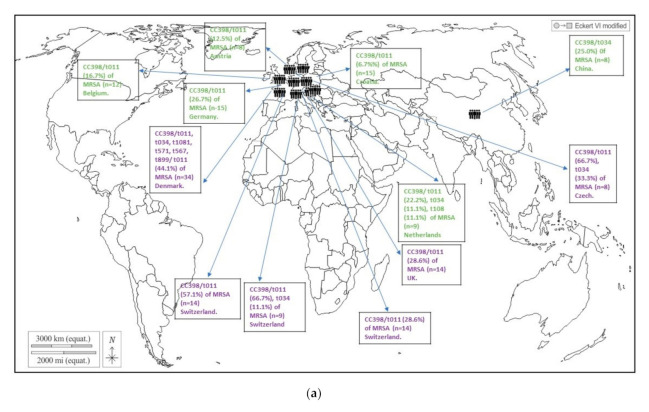

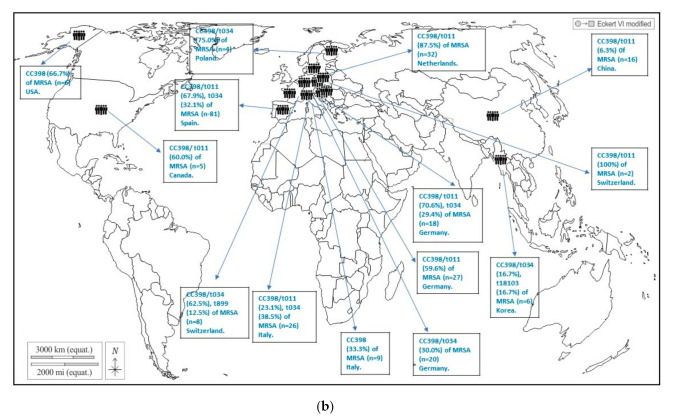
Geographic distribution of CC398 isolates detected in healthy humans of groups A, C, and D (*spa* types identified). (**a**) Group A (green) and C (blue); (**b**) Group D (purple). No CC398-MRSA was reported in Group B. Citations for Figure 8a: China [49], Austria [62], Belgium [62], Croatia [62], Netherlands [62], Germany [62], Switzerland [121,122,123], Denmark [124], Czech Republic [135], UK [136]. Citations for Figure 8b: Poland [125], Korea [152], China [155], Switzerland [121,168], Italy [161,167], Germany [172,174,175], Spain [181], Netherlands [181], USA [184], Canada [186].

According to the global data analyzed in this study, healthy people of Groups A and B showed very low rates of nasal carriages of MRSA-CC398 (<0.05%) and MSSA-CC398 (<1%); the predominant *spa* type among MSSA-CC398 isolates was t571, which represent a livestock-independent clade [17,205]. Findings from this analysis suggest that food handlers seem to be at a low risk of nasal colonization by the MRSA-CC398 lineage, as previously indicated [110]. However, a higher rate of MRSA-CC398 nasal carriage was detected in healthy humans of Group C (veterinarians) and especially among those of Group D (livestock farmers), with pooled rates of 2.6 and 5.4%, respectively (Figure 5), of which *spa*-types t034 and t011 predominated ( Supplementary Table S6; Figure 8). This prevalence increased when only pig farmers were considered (8.42%) (Figure 6). Moreover, a moderate prevalence of MSSA-CC398 was also found in farmers of Group D (1.8%), with t034 and t011 as predominant *spa* types (livestock-related clades), with no detection of t571; MSSA-CC398 represented 11.5% of total MSSA isolates (Figure 5, Supplementary Table S6). It seems that direct and intensive contact with livestock (especially pigs) is a risk factor for colonization with MRSA-CC398, and at a lower level with MSSA-CC398 (*spa* t011 or t034, animal-associated).

The global prevalence of MRSA-CC398 in pig farmers was 8.4%, with the following differences across continents: Europe, 15.1% (with 94.2% among MRSA); USA/Canada, 3.8% (with 33.7% among MRSA); and China–Korea–Taiwan, 1.5% (or 4.2% if we consider only CC398 or CC398+ST9) (Figure 7). The ST9-MRSA clone was first reported in 2010 in Italy [157]. However, instead of ST9, ST398 was the first to be reported from pigs, and have since then prevailed among pigs in European countries, the USA, Canada, and Asia [157]. ST9 (a CC398 hybrid) has also predominantly been reported in swine-associated environments in some Asia countries [157]. Among the MRSA-CC398 in pig farmers, 227 of the isolates had their *spa* typed, with majority being t011 (44.0%, n = 100 isolates) and t034 (28.6%, n = 65 isolates) However, 10 other *spa* types linked to CC398 were reported (t899, t1451, t1197, t108, among others).

### 3.9. Non-Aureus Staphylococci Nasal Colonization in Healthy People (Groups A to D)

Aside from *S. aureus* isolates, 16 different studies recovered and reported non-*aureus* staphylococci ( Supplementary Table S7). Most of them were *S. epidermidis.* Perhaps the high proportion of *S. epidermidis* isolated from the nasal cavity of healthy people was because this CoNS is a major part of the normal microbiota of the skin (including those on the nose). Moreover, *S. epidermidis* is one of the most abundant colonizers of healthy human mucosa including that in the respiratory tract [206].

Moreover, Abadi et al. [82] and Falomir et al. [83] reported *non-aureus* CoPS and CoNS from the nasal cavity of healthy humans who did not have animal contact (Supplementary Table S7). In most of the studies that reported CoNS, *S. haemolyticus, S. lugdunensis*, and *S. saprophyticus* were ones of the major isolated species, in addition to *S. epidermidis.* It could be that humans encountered these isolates through the environment [207] or during the consumption of contaminated animal-derived food, implying these as routes of transmission of non-*S. aureus* to humans [208].

Besides *S. aureus* isolated from the nasal cavity of livestock workers, Sinlapasorn et al. [188] reported the identification of *S. sciuri, S. haemolyticus, S. arlettae, S. cohnii* subsp*. urealyticus, S. hominis* subsp. *hominis, S. chromogenes*, and *S. epidermidis* isolates recovered from Thailand pig farmers (Supplementary Table S7). Interestingly, the rates of MRCoNS among pig-farm workers recorded by Sinlapasorn et al. [188] was high (16.3%). This finding confirmed that both MRSA and various species of MRCoNS can be frequently found in livestock farmers [188].

Veterinary personnel may also be colonized by non-*aureus* CoPS of animal origin. Aside from the *S. aureus* nasal colonization in veterinarians, three studies reported the recovery of *S. pseudintermedius* from veterinarians of Denmark, Czech Republic, and Hong Kong, representing nasal colonization between 0.7–3.9%; interestingly, two of these studies recovered methicillin-resistant *S. pseudintermedius* (MRSP) isolates [138,139]. Dogs are frequently colonized and occasionally infected by this microorganism that could be transferred to in-contact humans, as is the case of veterinarians [209]. Several studies have isolated *S. pseudintermedius* from 46–92% of healthy dogs, with the highest prevalence at the perineum, followed by either the nasal or oral mucosa [210,211].

### 3.10. Genetic Studies of Nasal S. pseudintermedius Colonization from Healthy People

The few genomic studies of *S. pseudintermedius* have offered a glimpse of its diversity, epidemiological characteristics, and antimicrobial resistance gene profile [185,212]. Among them, *S. pseudintermedius* was reported from studies by Paul et al. [138], Boost et al. [139], and Neradova et al. [135] with a prevalence of 3.9, 0.7, and 1.5%, respectively ( Supplementary Table S7). However, only a study by Boost et al. [139] performed the molecular typing of the *S. pseudintermedius* isolate. Of which, it was MRSP and belonged to ST71.

Although the three studies reported a low *S. pseudintermedius* carriage rate (<5%), they may be epidemiologically considered high on the basis that *S. pseudintermedius* is not a normal human microbiota. Hence, the *S. pseudintermedius* colonized veterinary personnel may have an increased risk for the acquisition of MRSP because they are regularly exposed to animals (especially dogs and cats) and other small animals with skin and soft tissue infections [213].

## 4. Limitation of the Study

Despite the fact that this current article comprehensively synthesized data from published articles to obtain various pooled prevalence, it is necessary to be cautious in the interpretation of some data, as the pooled prevalence of *S. aureus* and MRSA in some countries were solely from one or two studies (See supplementary files).

## 5. Conclusions and Recommendations

These findings, put together, demonstrated that contact with livestock and veterinary practice increases the risk of being an MRSA-CC398 nasal carrier, while food handlers seem to be at a lower risk for MRSA-CC398 carriage. Thus, this emphasizes the need for an integrated molecular epidemiology of zoonotic staphylococci to enhance adequate surveillance and accurate diagnostics. It is very important to implement a long-term surveillance of the genetic lineages of nasal staphylococci. Moreover, there is the crucial need to precisely determine the virulome and resistome of *S. pseudintermedius* and other non-*aureus* staphylococci colonizing healthy humans.

Most of the eligible studies used in this current article were performed in European and Asian countries. Hence, it is recommended that more studies should be performed in other continents to have a more and balanced picture of this subject matter. To achieve a formidable control of antimicrobial resistance, country-wise, regional, and global antimicrobial-resistant surveillance are very critical. These will help to detect and measure the extent of the emergence of new resistant lineages with the capacity to spread and switch between healthy animals and humans. Taken together, our findings provide a framework that will provide a critical foundation for future studies on the population structure and dynamics, antimicrobial resistance of nasal staphylococci as well as the occupational risks in food handlers, veterinarians, and livestock farmers.

## Figures and Tables

**Table 1:** Summary of the pooled global prevalence of *Staphylococcus aureus* and MRSA nasal carriages in the four studied groups (A to D).

Study Groups	Number of Studies Included ^a^	Pooled *S. aureus* Nasal Carriage Rate (%) (Range)	Number of *S. aureus* Studies Included	Total Number		Pooled MRSA Nasal Carriage Rate (%) (Range)	Number of MRSA Studies Included	Total Number	
								People	MRSA
				People	*S. aureus*				
A	58	15.9 (2.3–79.6)	55	133,310	21,133	0.8 (0.0–17.5)	52	131,578	1020
B	31	7.8 (1.4–60.0)	31	35,875	2803	0.9 (0.0–37.1)	21	18,211	167
C	26	34.9 (19.4–50.8)	7	614	214	8.6 (0.7–38.4)	25	3735	343
D	51	27.1 (3.1–62.5)	25	4310	1169	13.5 (0.0–85.8)	49	7033	946

^a^ Studies included correspond to those of Supplementary Tables S1–S4.

**Table 2:** Prevalence categories of MRSA nasal carriage studies performed in healthy persons with or without occupation risks of colonization (Groups A–D).

Prevalence Range of MRSA		Healthy People without Risk (Group A, 52 Studies)		Food Handlers (Group B, 21 Studies)		Veterinarians (Group C, 25 Studies)		Livestock Farmers (Group D, 49 Studies)	
		No. of Studies (%)/Countries	Reference	No. of Studies (%)/Countries	Reference	No. of Studies (%)/Countries	References	No. of Studies (%)/Countries	Reference
Low <2%		30 (57.7)/Ghana, Gambia, Gabon, Egypt, Nigeria, China, Thailand, Pakistan, Malaysia, Spain, Serbia, Greece, Australia, USA, Brazil, Denmark, France, India, Germany, Austria, Croatia, Ukraine, Hungary, Sweden, UK, The Netherlands	[20,33,34,35,38,39,46,47,48,49,50,52,53,55,56,59,60,61,62,63,65,67,69,70,71,73,74,80,84,85]	12 (57.1%)/Sudan, Ethiopia, Malaysia, Hong Kong, Turkey, Burma, Germany, Greece, Bosnia, Portugal, Brazil	[13,88,90,100,102,103,106,107,108,110,114,115]	4 (16.0)/Denmark, Hong Kong, China, Czech Republic	[119,127,138,139]	8 (16.3)/Nigeria, China, Switzerland, Italy, Morocco, Ethiopia	[142,145,148,154,155,163,168,179]
Medium 2.0–5.0%		10 (19.2)/Colombia, Spain, Morocco, Ethiopia, India, Ukraine, Mexico, Argentina	[36,37,51,64,66,76,77,78,79,83]	3 (14.3)/Lebanon, USA, Iran	[96,97,111]	6 (24.0)/Switzerland, UK, Denmark	[122,123,126,129,130,136]	7 (14.3)/Korea, Thailand, France, Italy, Poland, USA, Nigeria	[125,146,159,162,177,184,188]
High 5.1–10.0%		6 (11.5)/Spain, Iran, Nigeria, Brazil	[30,41,43,66,75,82]	2 (9.5%)/China, Hong Kong	[98,101]	7 (28.0)/USA, Canada, Australia, Switzerland, India, Czech Republic, Madagascar	[116,120,121,131,133,134,135]	8 (16.3)/Thailand, Turkey, Nigeria, Italy, Switzerland, USA, The Netherlands, Greece	[121,144,150,153,161,164,166,182]
Very High	10.1–20.0%	6/(11.5)/Nigeria, Malaysia, Iraq, Iran, USA	[31,42,44,45,54,68]	2 (9.5)/Sudan, Iraq	[89,95]	4 (16.0)/USA, Poland, Denmark	[124,125,132,137]	9 (18.4%)/Iraq, Korea, Taiwan, China, Sri Lanka, Denmark, Nigeria, Canada, Italy	[147,151,152,157,160,167,170,183,186]
	20.1–30.0%	NA	NA	1 (4.8)/Portugal	[109]	1 (4.0)/Nigeria	[117]	8 (16.3)/Nigeria, Madagascar, Ethiopia, Germany, Italy, USA	[141,143,149,172,174,176,178,185]
Extremely High	30.1–40.0%	NA	NA	1 (4.8)/Nigeria	[92]	2 (8.0)/Nigeria, Japan	[20,118]	2 (4.1)/The Netherlands, Italy	[169,180]
	40.1–50.0%	NA	NA	NA	NA	1 (4.0)/Germany	[128]	2 (4.1)/Germany, The Netherlands	[165,173]
	>50%	NA	NA	NA	NA	NA	NA	5 (10.2)/Germany, Spain, Australia	[128,171,175,181,187]

## Data Availability

The datasets in excel sheet used for the analyses of the main findings during this study can be accessed through the corresponding author on request.

## Supplementary Materials

The following are available online at https://www.mdpi.com/article/10.3390/pathogens10081000/s1, Table S1: Study characteristics, species, antimicrobial resistance and virulence genes of Staphylococcal nasal carriages in healthy humans that did not report any risk factor of S. aureus colonization (n = 58), Table S2: Study characteristics, species, antimicrobial resistance pattern and virulence genes detected in staphylococcal nasal isolates recovered from healthy food-handlers (n = 31), Table S3: Study characteristics, species, antimicrobial resistance, and virulence genes of staphylococcal nasal carriages in veterinary students and practitioners (n = 26), Table S4: Study characteristics, species, antimicrobial resistance pattern, and virulence genes detected in staphylococcal nasal isolates recovered from healthy livestock farmers (n = 51), Table S5: Prevalence pattern and genetic profile of PVL-positive S. aureus isolates in the nasal cavity of healthy people of the Groups A–D, Table S6: Molecular typing of S. aureus isolated from the nasal cavity of healthy humans with or without occupational risks of colonization (Groups A–D), Table S7: Study characteristics, species, and antimicrobial resistance profile of non-aureus staphylococci nasal carriage in healthy people with or without risk of colonization (Groups A–D).

## References

[1] Kumpitsch C., Koskinen K., Schöpf V., Moissl-Eichinger C. (2019). The microbiome of the upper respiratory tract in health and disease. BMC Biol..

[2] Zondervan N.A., Martins Dos Santos V.A.P., Suarez-Diez M., Saccenti E. (2021). Phenotype and multi-omics comparison of *Staphylococcus* and *Streptococcus* uncovers pathogenic traits and predicts zoonotic potential. BMC Genom..

[3] Sakr A., Brégeon F., Mège J.-L., Rolain J.-M., Blin O. (2018). *Staphylococcus aureus* nasal colonization: An update on mechanisms, epidemiology, risk factors, and subsequent infections. Front. Microbiol..

[4] Tong S.Y.C., Davis J.S., Eichenberger E., Holland T.L., Fowler V.G. (2015). *Staphylococcus aureus* infections: Epidemiology, pathophysiology, clinical manifestations, and management. Clin. Microbiol. Rev..

[5] Steed L.L., Costello J., Lohia S., Jones T., Spannhake E.W., Nguyen S. (2014). Reduction of nasal *Staphylococcus aureus* carriage in health care professionals by treatment with a nonantibiotic, alcohol-based nasal antiseptic. Am. J. Infect. Control..

[6] McNeil J.C. (2014). *Staphylococcus aureus*–Antimicrobial resistance and the immunocompromised Child. Infect. Drug Resist..

[7] Leshem E., Maayan-Metzger A., Rahav G., Dolitzki M., Kuint J., Roytman Y., Goral A., Novikov I., Fluss R., Keller N. (2012). Transmission of *Staphylococcus aureus* from Mothers to Newborns. Pediatr. Infect. Dis. J..

[8] Peng H., Liu D., Ma Y., Gao W. (2018). Comparison of community- and healthcare-associated Methicillin-Resistant *Staphylococcus aureus* isolates at a Chinese tertiary hospital, 2012–2017. Sci. Rep..

[9] Siddiqui A.H., Koirala J., Siddiqui A.H., Koirala J. (2020). Methicillin resistant *Staphylococcus aureus*. StatPearls.

[10] Moosavian M., Shahin M., Navidifar T., Torabipour M. (2018). Typing of Staphylococcal Cassette Chromosome *mec* encoding Methicillin resistance in *Staphylococcus aureus* isolates in Ahvaz, Iran. New Microbes New Infect..

[11] Wang X., Li X., Liu W., Huang W., Fu Q., Li M. (2016). Molecular characteristic and virulence gene profiles of community-associated Methicillin-Resistant *Staphylococcus aureus* isolates from pediatric patients in Shanghai, China. Front. Microbiol..

[12] Otter J.A., Kearns A.M., French G.L., Ellington M.J. (2010). Panton-Valentine Leukocidin-encoding Bacteriophage and gene sequence variation in community-associated Methicillin-Resistant *Staphylococcus aureus*. Clin. Microbiol. Infect..

[13] Castro A., Santos C., Meireles H., Silva J., Teixeira P. (2016). Food handlers as potential sources of dissemination of virulent strains of *Staphylococcus aureus* in the Community. J. Infect. Public Health.

[14] Zarazaga M., Gómez P., Ceballos S., Torres C., Fetsch A. (2018). Molecular epidemiology of *Staphylococcus aureus* lineages in the animal-human interface. Staphylococcus aureus.

[15] Bouchami O., Fraqueza M.J., Faria N.A., Alves V., Lawal O.U., de Lencastre H., Miragaia M. (2020). Evidence for the dissemination to humans of methicillin-resistant *Staphylococcus aureus* ST398 through the pork production chain: A study in a Portuguese slaughterhouse. Microorganisms.

[16] Ceballos S., Aspiroz C., Ruiz-Ripa L., Reynaga E., Azcona-Gutiérrez J.M., Rezusta A., Seral C., Antoñanzas F., Torres L., López C. (2019). Epidemiology of MRSA CC398 in hospitals located in Spanish regions with different pig-farming densities: A Multicentre study. J. Antimicrob. Chemother..

[17] Mama O.M., Aspiroz C., Ruiz-Ripa L., Ceballos S., Iñiguez-Barrio M., Cercenado E., Azcona J.M., López-Cerero L., Seral C., López-Calleja A.I. (2021). Prevalence and genetic characteristics of *Staphylococcus aureus* CC398 isolates from invasive infections in Spanish hospitals, focusing on the livestock-independent CC398-MSSA Clade. Front. Microbiol..

[18] Bouiller K., Bertrand X., Hocquet D., Chirouze C. (2020). Human infection of Methicillin-Susceptible *Staphylococcus aureus* CC398: A review. Microorganisms.

[19] Kadariya J., Smith T.C., Thapaliya D. (2014). *Staphylococcus aureus* and staphylococcal food-borne disease: An ongoing challenge in public health. Biomed. Res. Int..

[20] Anueyiagu K.N., Kopmut J.J., Lagi C.A., Okoh K.N. (2020). Nasal carriage of MRSA among healthy college students and livestock. Vet. Sci. Res. Rev..

[21] Bencardino D., Amagliani G., Brandi G. (2021). Carriage of *Staphylococcus aureus* among food handlers: An ongoing challenge in public health. Food Control..

[22] Somayaji R., Rubin J.E., Priyantha M.A., Church D. (2016). Exploring *Staphylococcus pseudintermedius:* An emerging zoonotic pathogen?. Future Microbiol..

[23] Lozano C., Rezusta A., Ferrer I., Pérez-Laguna V., Zarazaga M., Ruiz-Ripa L., Revillo M.J., Torres C. (2017). *Staphylococcus pseudintermedius* human infection cases in Spain: Dog-to-Human transmission. Vector Borne Zoonotic Dis..

[24] Byrd A.L., Belkaid Y., Segre J.A. (2018). The human skin microbiome. Nat. Rev. Microbiol..

[25] Michalik M., Samet A., Podbielska-Kubera A., Savini V., Międzobrodzki J., Kosecka-Strojek M. (2020). Coagulase-negative Staphylococci (CoNS) as a significant etiological factor of laryngological infections: A review. Ann. Clin. Microbiol. Antimicrob..

[26] Méric G., Mageiros L., Pensar J., Laabei M., Yahara K., Pascoe B., Kittiwan N., Tadee P., Post V., Lamble S. (2018). Disease-associated genotypes of the commensal skin bacterium *Staphylococcus epidermidis*. Nat. Commun..

[27] Sabaté Brescó M., Harris L.G., Thompson K., Stanic B., Morgenstern M., O′Mahony L., Richards R.G., Moriarty T.F. (2017). Pathogenic mechanisms and host interactions in *Staphylococcus epidermidis* device-related infection. Front. Microbiol..

[28] Awulachew E.W., Diriba K., Anja A., Wudneh F. (2020). Nasopharyngeal carriage of *Staphylococcus aureus* and its antimicrobial resistance pattern among healthy people: Systematic review and meta-analysis. J Bacteriol. Parasitol..

[29] Elsanousi R.M.A., Elsanousi S.M. (2020). Nasal carriage of *Staphylococcus* species among Sudanese community in Khartoum State, Sudan. J. Microbiol. Res..

[30] Ogefere H.O., Ogunleye L.A. (2019). Molecular characterization of Methicillin-Resistant staphylococci among apparently healthy students. Universa Med..

[31] Onanuga A., Temedie T.C. (2011). Nasal carriage of multi-drug resistant *Staphylococcus aureus* in healthy inhabitants of Amassoma in Niger Delta Region of Nigeria. Afr. Health Sci..

[32] Nsofor C. (2015). Nasal carriage of *Staphylococcus aureus* among apparently healthy school children in Owerri metropolis, Nigeria. MOJ Cell Sci. Rep..

[33] Eibach D., Nagel M., Hogan B., Azuure C., Krumkamp R., Dekker D., Gajdiss M., Brunke M., Sarpong N., Owusu-Dabo E. (2017). Nasal Carriage of *Staphylococcus aureus* among children in the Ashanti region of Ghana. PLoS ONE.

[34] Egyir B., Guardabassi L., Esson J., Nielsen S.S., Newman M.J., Addo K.K., Larsen A.R. (2014). Insights into nasal carriage of *Staphylococcus aureus in* an Urban and a rural community in Ghana. PLoS ONE.

[35] Ateba Ngoa U., Schaumburg F., Adegnika A.A., Kösters K., Möller T., Fernandes J.F., Alabi A., Issifou S., Becker K., Grobusch M.P. (2012). Epidemiology and population structure of *Staphylococcus aureus* in various population groups from a rural and semi urban area in Gabon, Central Africa. Acta Trop..

[36] Mourabit N., Arakrak A., Bakkali M., Laglaoui A. (2017). Nasal carriage of sequence type 22 MRSA and livestock-associated ST398 clones in Tangier, Morocco. J. Infect. Dev. Ctries..

[37] Tigabu A., Tiruneh M., Mekonnen F. (2018). Nasal carriage rate, antimicrobial susceptibility pattern, and associated factors of *Staphylococcus aureus* with special emphasis on MRSA among urban and rural elementary school children in Gondar, Northwest Ethiopia: A comparative cross-sectional study. Adv. Prev. Med..

[38] Ebruke C., Dione M.M., Walter B., Worwui A., Adegbola R.A., Roca A., Antonio M. (2016). High genetic diversity of *Staphylococcus aureus* strains colonising the nasopharynx of Gambian villagers before widespread use of pneumococcal conjugate vaccines. BMC Microbiol..

[39] El-Mahdy T.S., Al-Agamy M.H., Emara M., Barakat A., Goering R.V. (2018). Complex clonal diversity of *Staphylococcus aureus* nasal colonization among community personnel, healthcare workers, and clinical students in the Eastern province, Saudi Arabia. BioMed Res. Int..

[40] AL-Haj N.A., Hauter J.M., Al-Bulili N.H., Al-Hotami R.A., Al-Horaibi M.T. (2018). Nasal carriage of *Staphylococcus aureus* among students of public schools in Sana′a. Yemen. Res J Microbiol..

[41] Soltani B., Taghavi Ardakani A., Moravveji A., Erami M., Haji Rezaei M., Moniri R., Namazi M. (2014). Risk factors for Methicillin resistant *Staphylococcus aureus* nasal colonization of healthy children. Jundishapur J. Microbiol..

[42] Khorvash F., Abdi F., Kashani H.H., Naeini F.F., Narimani T. (2012). *Staphylococcus aureus* in acne pathogenesis: A case-control study. N. Am. J. Med. Sci..

[43] Mobasherizadeh S., Shojaei H., Havaei S.A., Mostafavizadeh K., Davoodabadi F., Khorvash F., Kushki A.M., Daei-Naser A., Ghanbari F. (2016). Nasal carriage screening of community-associated methicillin resistant *Staphylococcus aureus* in healthy children of a developing country. Adv. Biomed. Res..

[44] Alshami A.A. (2018). Rate of methicillin-resistant *Staphylococcus aureus* (MRSA) nasal carriage among healthy students in faculty of veterinary medicine/University of Kufa and the importance of Annual Screening. Int. J. Pharm. Sci. Med. (IJPSM).

[45] Rasheed N.A., Hussein N.R. (2020). Methicillin-resistant *Staphylococcus aureus* carriage rate and molecular characterization of the staphylococcal cassette chromosome *mec* among Syrian refugees in Iraq. Int. J. Infect. Dis..

[46] Suvarnsit K., Kiratisin P., Bunnag C., Tantilipikorn P. (2019). Prevalence of nasal carriage of *Staphylococcus aureus* in allergic rhinitis patients and healthy controls in Thailand. Asian Pac. J. Allergy Immunol..

[47] Chen B.J., Xie X.Y., Ni L.J., Dai X.L., Lu Y., Wu X.Q., Li H.Y., Yao Y.D., Huang S.Y. (2017). Factors associated with *Staphylococcus aureus* nasal carriage and molecular characteristics among the general population at a medical college campus in Guangzhou, South China. Ann. Clin. Microbiol. Antimicrob..

[48] Gong Z., Shu M., Xia Q., Tan S., Zhou W., Zhu Y. (2017). *Staphylococcus aureus* nasal carriage and its antibiotic resistance profiles in children in high altitude areas of Southwestern China. Arch. Argent. Pediatr..

[49] Yan X., Song Y., Yu X., Tao X., Yan J., Luo F., Zhang H., Zhang J., Li Q., He L. (2015). Factors associated with *Staphylococcus aureus* nasal carriage among healthy people in Northern China. Clin. Microbiol. Infect..

[50] Jamil J., Zaman K., Ullah S., Ali I., Kalsoom (2020). Colonization of *Staphylococcus aureus* in nasal cavities of healthy individuals from district Swabi, KP, Paki-stan. J. Pak. Med. Assoc..

[51] Chatterjee S.S., Ray P., Aggarwal A., Das A., Sharma M. (2009). A community-based study on nasal carriage of *Staphylococcus aureus*. Indian J. Med. Res..

[52] Fomda B.A., Thokar M.A., Khan A., Bhat J.A., Zahoor D., Bashir G., Majid A., Ray P. (2014). Nasal carriage of methicillin-resistant *Staphylococcus aureus* among healthy population of Kashmir, India. Indian J. Med. Microbiol..

[53] Choi C.S., Yin C.S., Bakar A.A., Sakewi Z., Naing N.N., Jamal F., Othman N. (2006). Nasal carriage of *Staphylococcus aureus* among healthy adults. J. Microbiol. Immunol. Infect..

[54] Anbazhagan D., Hui M.J., Aisyah N., Syazwani A., Keong T.P., Shuen L.J., Sundram M.S., Subramaniam H., Kumarasamy V. (2020). Nasal carriage of methicillin-resistant *Staphylococcus aureus* among undergraduates in Malaysia. Int. Res. J. med. Med. Sci..

[55] Wang H.-K., Huang C.-Y., Chen C.-J., Huang Y.-C. (2017). Nasal *Staphylococcus aureus* and Methicillin-Resistant *Staphylococcus aureus* carriage among college student athletes in Northern Taiwan. J. Microbiol. Immunol. Infect..

[56] Dinić M., Vuković S., Stanković Đorđević D., Bogdanović M. (2013). Nasal carriage of *Staphylococcus aureus* in healthy adults and in school children. Acta Fac. Med. Naissensis.

[57] Ritchie S.R., Isdale E., Priest P., Rainey P.B., Thomas M.G. (2016). The turnover of strains in intermittent and persistent nasal carriers of *Staphylococcus aureus*. J. Infect..

[58] Andersen P.S., Larsen L.A., Fowler V.G., Stegger M., Skov R.L., Christensen K. (2013). Risk factors for *Staphylococcus aureus* nasal colonization in Danish middle-aged and elderly Twins. Eur. J. Clin. Microbiol. Infect. Dis..

[59] Karapsias S., Piperaki E.T., Spiliopoulou I., Katsanis G., Tseleni-Kotsovili A. (2008). Methicillin-resistant *Staphylococcus aureus* nasal carriage among healthy employees of the Hellenic Air Force. Euro Surveill..

[60] Kirkliauskienė A., Ambrozaitis A., Skov R.L., Frimodt-Møller N. (2010). The prevalence of *Staphylococcus aureus* nose and throat carriage by healthy adults. Visuomenės Sveik..

[61] Mehraj J., Akmatov M.K., Strömpl J., Gatzemeier A., Layer F., Werner G., Pieper D.H., Medina E., Witte W., Pessler F. (2014). Methicillin-sensitive and methicillin-resistant *Staphylococcus aureus* nasal carriage in a random sample of non-hospitalized adult population in northern Germany. PLoS ONE.

[62] den Heijer C.D.J., van Bijnen E.M.E., Paget W.J., Pringle M., Goossens H., Bruggeman C.A., Schellevis F.G., Stobberingh E.E., APRES Study Team (2013). Prevalence and resistance of commensal *Staphylococcus aureus,* including meticillin-resistant *S. aureus,* in nine European countries: A cross-sectional study. Lancet Infect. Dis..

[63] Becker K., Schaumburg F., Fegeler C., Friedrich A.W., Köck R. (2017). *Staphylococcus aureus* from the German general population is highly diverse. Int. J. Med. Microbiol..

[64] Netsvyetayeva I., Fraczek M., Piskorska K., Golas M., Sikora M., Mlynarczyk A., Swoboda-Kopec E., Marusza W., Palmieri B., Iannitti T. (2014). *Staphylococcus aureus* nasal carriage in Ukraine: Antibacterial resistance and virulence factor encoding genes. BMC Infect. Dis..

[65] Falomir M.P., Gozalbo D., Rico H. (2014). Occurrence of methicillin-resistant *Staphylococcus aureus* in the nasal cavity of healthy volunteer students of the University of Valencia (Spain). J. Microbiol. Immunol. Infect..

[66] Teira R., Teira A., Campo A.B., de Benito I. (2013). Prevalence of nasopharyngeal colonization by methicillin-resistant *Staphylococcus aureus* in a population of high school students in Torrelavega (Spain). Enferm. Infecc. Microbiol. Clin..

[67] Lozano C., Gómez-Sanz E., Benito D., Aspiroz C., Zarazaga M., Torres C. (2011). *Staphylococcus aureus* nasal carriage, virulence traits, antibiotic resistance mechanisms, and genetic lineages in healthy humans in Spain, with detection of CC398 and CC97 strains. Int. J. Med. Microbiol..

[68] Muthukrishnan G., Lamers R.P., Ellis A., Paramanandam V., Persaud A.B., Tafur S., Parkinson C.L., Cole A.M. (2013). Longitudinal genetic analyses of *Staphylococcus aureus* nasal carriage dynamics in a diverse population. BMC Infect. Dis..

[69] Velasco V., Buyukcangaz E., Sherwood J.S., Stepan R.M., Koslofsky R.J., Logue C.M. (2015). Characterization of *Staphylococcus aureus* from humans and a comparison with isolates of animal origin, in North Dakota, United States. PLoS ONE.

[70] Kuehnert M.J., Kruszon-Moran D., Hill H.A., McQuillan G., McAllister S.K., Fosheim G., McDougal L.K., Chaitram J., Jensen B., Fridkin S.K. (2006). Prevalence of *Staphylococcus aureus* nasal colonization in the United States, 2001–2002. J. Infect. Dis..

[71] Gorwitz R.J., Kruszon-Moran D., McAllister S.K., McQuillan G., McDougal L.K., Fosheim G.E., Jensen B.J., Killgore G., Tenover F.C., Kuehnert M.J. (2008). Changes in the prevalence of nasal colonization with *Staphylococcus aureus* in the United States, 2001–2004. J. Infect. Dis..

[72] Wardyn S.E., Forshey B.M., Farina S.A., Kates A.E., Nair R., Quick M.K., Wu J.Y., Hanson B.M., O′Malley S.M., Shows H.W. (2015). Swine farming is a risk factor for infection with and high prevalence of carriage of multidrug-resistant *Staphylococcus aureus*. Clin. Infect. Dis..

[73] Wardyn S.E., Forshey B.M., Smith T.C. (2012). High prevalence of Panton-valentine leukocidin among methicillin-sensitive *Staphylococcus aureus* colonization isolates in Rural Iowa. Microb. Drug Resist..

[74] Pires F.V., da Cunha M.D., Abraão L.M., Martins P.Y.F., Camargo C.H., Fortaleza C.M.C.B. (2014). Nasal carriage of *Staphylococcus aureus* in Botucatu, Brazil: A population-based survey. PLoS ONE.

[75] Braga E.D.V., Aguiar-Alves F., de Freitas M.D.F.N., de e Silva M.O., Correa T.V., Snyder R.E., de Araújo V.A., Marlow M.A., Riley L.W., Setúbal S. (2014). High prevalence of *Staphylococcus aureus* and methicillin-resistant *S. aureus* colonization among healthy children attending public daycare centers in informal settlements in a large urban center in Brazil. BMC Infect. Dis..

[76] Hamdan-Partida A., Sainz-Espuñes T., Bustos-Martínez J. (2010). Characterization and persistence of *Staphylococcus aureus* strains isolated from the anterior nares and throats of healthy carriers in a Mexican community. J. Clin. Microbiol..

[77] Rebollo-Pérez J., Ordoñez-Tapia C., Herazo-Herazo C., Reyes-Ramos N. (2011). Nasal Carriage of Panton Valentine leukocidin-positive methicillin-resistant *Staphylococcus aureus* in healthy preschool children. Rev. Salud Publica (Bogota).

[78] Hanselman B.A., Kruth S.A., Rousseau J., Weese J.S. (2008). Methicillin-resistant *Staphylococcus aureus* colonization in Schoolteachers in Ontario. Can. J. Infect. Dis. Med. Microbiol..

[79] Gardella N., Murzicato S., Di Gregorio S., Cuirolo A., Desse J., Crudo F., Gutkind G., Mollerach M. (2011). Prevalence and characterization of methicillin-resistant *Staphylococcus aureus* among healthy children in a city of Argentina. Infect. Genet. Evol..

[80] Munckhof W.J., Nimmo G.R., Schooneveldt J.M., Schlebusch S., Stephens A.J., Williams G., Huygens F., Giffard P. (2009). Nasal carriage of *Staphylococcus aureus*, including community-associated methicillin-resistant strains, in Queensland adults. Clin. Microbiol. Infect..

[81] Ogefere H.O., Umaru G., Ibadin E.E., Omoregie R. (2020). Prevalence of methicillin-resistant staphylococci among apparently healthy students attending a tertiary institution in Benin City, Nigeria. Nig. J. Bas. App. Sci..

[82] Abadi M.I.M., Moniri R., Khorshidi A., Piroozmand A., Mousavi S.G.A., Dastehgoli K., Ghazikalayeh H.M. (2015). Molecular characteristics of nasal carriage methicillin-resistant coagulase negative staphylococci in school students. Jundishapur J Microbiol.

[83] Falomir D.G., Vidal H.R., Llorens M.P.F. (2019). Commensal *Staphylococcus* isolates from the nasal cavity of community older adults in Valencia (Spain) and their resistance to methicillin and other antibiotics. Eur. J. Health Res..

[84] Falomir M.P., Jávega A., Rico H., Gozalbo D. (2018). Nasal Isolates of Comensal *Staphylococcus aureus* and non-*aureus* species from healthy young adults in Valencia (Spain) and their resistance to chemotherapeutic agents. Ann. Epidemiol. Public Health.

[85] Lebeaux D., Barbier F., Angebault C., Benmahdi L., Ruppé E., Felix B., Gaillard K., Djossou F., Epelboin L., Dupont C. (2012). Evolution of nasal carriage of methicillin-resistant coagulase-negative staphylococci in a remote population. Antimicrob. Agents Chemother..

[86] El-Shenawy M., El-Hosseiny L., Tawfeek M., El-Shenawy M., Baghdadi H., Saleh O., Mañes J., Soriano J.M. (2013). Nasal carriage of enterotoxigenic *Staphylococcus aureus* and risk factors among food handlers-Egypt. Food Public Health.

[87] Dagnew M., Tiruneh M., Moges F., Tekeste Z. (2012). Survey of Nasal carriage of *Staphylococcus aureus* and intestinal parasites among food handlers working at Gondar University, Northwest Ethiopia. BMC Public Health.

[88] Beyene G., Mamo G., Kassa T., Tasew G., Mereta S.T. (2019). Nasal and hand carriage rate of *Staphylococcus aureus* among food handlers working in Jimma Town, Southwest Ethiopia. Ethiop. J. Health Sci..

[89] Ahmed O.B. (2020). Prevalence of methicillin-resistant *Staphylococcus aureus* and classical enterotoxin genes among Sudanese Food handlers. Cureus.

[90] Al Hassan A.A., Gasim A.O., Osman M.A. (2016). Screening of food handlers for *Salmonella* and *Staphylococcus aureus* carriers at university cafeterias in Khartoum. Afr. J. Med. Sci..

[91] Emeakaroha M.C., Nkwocha I.G., Adieze N.C., Adieze I.E. (2017). Antimicrobial susceptibility pattern of *Staphylococcus aureus,* and their nasal and throat carriage among food handlers at the Federal University of Technology, Owerri Nigeria. Int. J. Biomol. Biomed. (IJBB).

[92] Omololu Aso J., Oluwatoyin Omololu Aso O., Hellen Chineye Ochada O.O.O., Shesha A. (2017). Nasal colonization of methicillin resistance *Staphylococcus aureus* among food handlers in the eateries Obafemi Awolowo University Ile Ife, Nigeria. J. Clin. Nutr. Diet..

[93] Eke S.O., Eloka C.C., Mgbachi N., Nwobodo H.A., Ekpen-Itamah U.J. (2015). Nasal carriage of *Staphylococcus aureus* among food handlers and restaurant workers in Ekpoma Edo State, Nigeria. IJCR.

[94] Abdulrahman M.A., Taher A.I. (2018). Prevalence of methicillin resistant *Staphylococcus aureus* among food handlers in Duhok City. Sci. J. Univ. Zakho.

[95] Mohammed M.J., Ali A.A. (2020). Isolation of *Staphylococcus aureus* bacteria from nasal swabs from workers in restaurants in Kirkuk City. Int. J. Drug Delivery Technol..

[96] Fooladvand S., Sarmadian H., Habibi D., van Belkum A., Ghaznavi-Rad E. (2019). High prevalence of methicillin resistant and enterotoxin gene-positive *Staphylococcus aureus* among nasally colonized food handlers in central Iran. Eur. J. Clin. Microbiol. Infect. Dis..

[97] Osman M., Kamal-Dine K., El Omari K., Rafei R., Dabboussi F., Hamze M. (2019). Prevalence of *Staphylococcus aureus* methicillin-sensitive and methicillin-resistant nasal carriage in food handlers in Lebanon: A potential source of transmission of virulent strains in the community. Access Microbiol..

[98] Boost M., Ho J., Guardabassi L., O′Donoghue M. (2013). Colonization of butchers with livestock-associated methicillin-resistant *Staphylococcus aureus*. Zoonoses Public Health.

[99] Alhashimi H.M.M., Ahmed M.M., Mustafa J.M. (2017). Nasal carriage of enterotoxigenic *Staphylococcus aureus* among food handlers in Kerbala City. Karbala Int. J. Mod. Sci..

[100] Noor-Azira A.M., Mohammad-Faid A.R., Shuhaimi M., Syafinaz A.N., Hamat R.A., Malina O. (2012). *Staphylococcus aureus* in food and nares of Food Handlers in Kuala Pilah, Malaysia. Pertanika J. Trop. Agric. Sci..

[101] Wang W., Baloch Z., Jiang T., Zhang C., Peng Z., Li F., Fanning S., Ma A., Xu J. (2017). Enterotoxigenicity and antimicrobial resistance of *Staphylococcus aureus* isolated from retail food in China. Front. Microbiol..

[102] Ho J., O′Donoghue M.M., Boost M.V. (2014). Occupational exposure to raw meat: A newly recognized risk factor for *Staphylococcus aureus* nasal colonization amongst food handlers. Int. J. Hyg. Environ. Health.

[103] Vatansever L., Sezer Ç., Bilge N. (2016). Carriage rate and methicillin resistance of *Staphylococcus aureus* in food handlers in Kars City, Turkey. Springerplus.

[104] Sepin-Özen N., Tuğlu-Ataman Ş., Seyman D., Aldağ H., Emek M. (2013). Antalya ili gıda çalışanlarında nazal *Staphylococcus aureus* taşıyıcılığının ve MRSA oran-larının üç farklı yöntem kullanılarak incelenmesi. Turk Hij. Den. Biyol. Derg..

[105] Šegalo S., Maestro D., Obradović Z., Jogunčić A. (2020). Nasal carriage rate and antimicrobial resistance pattern of *Staphylococcus aureus* among the food handlers in Canton Sarajevo, Bosnia and Herzegovina. J. Health Sci..

[106] Uzunović S., Ibrahimagić A., Kamberović F., Rijnders M.I.A., Stobberingh E.E. (2014). Molecular characterization of methicillin-susceptible and methicillin-resistant *Staphylococcus aureus* in food handlers in Bosnia and Herzegovina. Open Infect. Dis. J..

[107] de Jonge R., Verdier J.E., Havelaar A.H. (2010). Prevalence of meticillin-resistant *Staphylococcus aureus* amongst professional meat handlers in the Netherlands, March–July 2008. Eurosurveillance.

[108] Sergelidis D., Abrahim A., Anagnostou V., Govaris A., Papadopoulos T., Papa A. (2012). Prevalence, distribution, and antimicrobial susceptibility of *Staphylococcus aureus* in ready-to-eat salads and in the environment of a salad manufacturing plant in Northern Greece. Czech J. Food Sci..

[109] Ribeiro E., Clérigo A., Ribeiro E., Clérigo A. (2017). Assessment of *Staphylococcus aureus* colonization in bakery workers: A case study. Vertentes E Desafios Da Segurança.

[110] Cuny C., Layer F., Hansen S., Werner G., Witte W. (2019). Nasal colonization of humans with occupational exposure to raw meat and to raw meat products with methicillin-susceptible and methicillin-resistant *Staphylococcus aureus*. Toxins.

[111] Leibler J.H., Jordan J.A., Brownstein K., Lander L., Price L.B., Perry M.J. (2016). *Staphylococcus aureus* nasal carriage among beefpacking workers in a Midwestern United States Slaughterhouse. PLoS ONE.

[112] Acco M., Ferreira F.S., Henriques J.A.P., Tondo E.C. (2003). Identification of multiple strains of *Staphylococcus aureus* colonizing nasal mucosa of food handlers. Food Microbiol..

[113] Rall V.L.M., Sforcin J.M., Augustini V.C.M., Watanabe M.T., Fernandes A., Rall R., Silva M.G., Araújo J.P. (2010). Detection of enterotoxin genes of *Staphylococcus* sp isolated from nasal cavities and hands of food handlers. Braz. J. Microbiol..

[114] da Silva S.D.S.P., Cidral T.A., Soares M.J.D.S., de Melo M.C.N. (2015). Enterotoxin-encoding genes in *Staphylococcus* spp. from food handlers in a university restaurant. Foodborne Pathog. Dis..

[115] Aung M.S., San T., Aye M.M., Mya S., Maw W.W., Zan K.N., Htut W.H.W., Kawaguchiya M., Urushibara N., Kobayashi N. (2017). Prevalence and genetic characteristics of *Staphylococcus aureus* and *Staphylococcus argenteus* isolates harboring Panton-Valentine Leukocidin, enterotoxins, and TSST-1 genes from food handlers in Myanmar. Toxins.

[116] Rasamiravaka T., Nirinarimanana A.J., Rasamindrakotroka A. (2016). Evaluation of methicillin-resistant *Staphylococcus aureus* nasal carriage in Malagasy Veterinary students. Afr. J. Clin. Exp. Microbiol..

[117] Gaddafi M.S., Yakubu Y., Garba B., Bello M.B., Musawa A.I., Lawal H. (2020). Occurrence and antimicrobial resistant patterns of methicillin resistant *Staphylococcus aureus* (MRSA) among practicing veterinarians in Kebbi State, Nigeria. Folia Vet..

[118] Kuroda T., Kinoshita Y., Niwa H., Shinzaki Y., Tamura N., Hobo S., Kuwano A. (2016). Meticillin-resistant *Staphylococcus aureus* colonisation and infection in thoroughbred racehorses and veterinarians in Japan. Vet. Rec..

[119] Zhang W., Hao Z., Wang Y., Cao X., Logue C.M., Wang B., Yang J., Shen J., Wu C. (2011). Molecular characterization of methicillin-resistant *Staphylococcus aureus* Strains from pet animals and veterinary staff in China. Vet. J..

[120] Sekhar M.S., Rao T.S., Sharif N.M. (2017). Nasal colonization of methicillin resistant *Staphylococcus aureus* (MRSA) among dogs and dog handlers in Andhra Pradesh, India. Pharma Innov..

[121] Kittl S., Brodard I., Heim D., Andina-Pfister P., Overesch G. (2020). Methicillin-resistant *Staphylococcus aureus* strains in Swiss pigs and their relation to isolates from farmers and veterinarians. Appl. Environ. Microbiol..

[122] Huber H., Koller S., Giezendanner N., Stephan R., Zweifel C. (2010). Prevalence and characteristics of meticillin-resistant *Staphylococcus aureus* in humans in contact with farm animals, in livestock, and in food of animal origin, Switzerland, 2009. Euro Surveill..

[123] Wettstein Rosenkranz K., Rothenanger E., Brodard I., Collaud A., Overesch G., Bigler B., Marschall J., Perreten V. (2014). Nasal carriage of methicillin-resistant *Staphylococcus aureus* (MRSA) among Swiss veterinary health care providers: Detection of livestock- and healthcare-associated clones. Schweiz. Arch. Tierheilkd..

[124] Wulf M.W.H., Sørum M., van Nes A., Skov R., Melchers W.J.G., Klaassen C.H.W., Voss A. (2008). Prevalence of methicillin-resistant *Staphylococcus aureus* among veterinarians: An international study. Clin. Microbiol. Infect..

[125] Mroczkowska A., Żmudzki J., Marszałek N., Orczykowska-Kotyna M., Komorowska I., Nowak A., Grzesiak A., Czyżewska-Dors E., Dors A., Pejsak Z. (2017). Livestock-associated *Staphylococcus aureus* on Polish pig farms. PLoS ONE.

[126] Post V., Harris L.G., Morgenstern M., Geoff Richards R., Sheppard S.K., Fintan Moriarty T. (2017). Characterization of nasal methicillin-resistant *Staphylococcus aureus* isolated from international human and veterinary surgeons. J. Med. Microbiol..

[127] Žemličková H., Fridrichová M., Tyllová K., Jakubů V., Machová I. (2009). Carriage of methicillin-resistant *Staphylococcus aureus* in Veterinary personnel. Epidemiol. Infect..

[128] Cuny C., Nathaus R., Layer F., Strommenger B., Altmann D., Witte W. (2009). Nasal colonization of humans with methicillin-resistant *Staphylococcus aureus* (MRSA) CC398 with and without exposure to pigs. PLoS ONE.

[129] Heller J., Armstrong S.K., Girvan E.K., Reid S.W.J., Moodley A., Mellor D.J. (2009). Prevalence and distribution of meticillin-resistant *Staphylococcus aureus* with-in the environment and staff of a University Veterinary Clinic. J. Small Anim. Pract..

[130] Moodley A., Nightingale E.C., Stegger M., Nielsen S.S., Skov R.L., Guardabassi L. (2008). High Risk for nasal carriage of methicillin-resistant *Staphylococcus aureus* among Danish Veterinary practitioners. Scand. J. Work Environ. Health.

[131] Sun J., Yang M., Sreevatsan S., Bender J.B., Singer R.S., Knutson T.P., Marthaler D.G., Davies P.R. (2017). Longitudinal Study of *Staphylococcus aureus* colonization and infection in a cohort of swine veterinarians in the United States. BMC Infect. Dis..

[132] Anderson M.E.C., Lefebvre S.L., Weese J.S. (2008). Evaluation of prevalence and risk factors for methicillin-resistant *Staphylococcus aureus* colonization in veterinary personnel attending an international equine veterinary conference. Vet. Microbiol..

[133] Hanselman B., Kruth S., Rousseau J., Low D., Willey B., McGeer A., Weese J. (2006). Methicillin-Resistant *Staphylococcus aureus* colonization in veterinary personnel. Emerg. Infect. Dis..

[134] Worthing K.A., Brown J., Gerber L., Trott D.J., Abraham S., Norris J.M. (2018). Methicillin-resistant staphylococci amongst veterinary personnel, personnel-owned pets, patients and the hospital environment of two small animal veterinary hospitals. Vet. Microbiol..

[135] Neradova K., Jakubu V., Pomorska K., Zemlickova H. (2020). Methicillin-resistant *Staphylococcus aureus* in veterinary professionals in 2017 in the Czech Republic. BMC Vet. Res..

[136] Paterson G.K., Harrison E.M., Craven E.F., Petersen A., Larsen A.R., Ellington M.J., Török M.E., Peacock S.J., Parkhill J., Zadoks R.N. (2013). Incidence and characterisation of methicillin-resistant *Staphylococcus aureus* (MRSA) from nasal colonisation in participants attending a cattle veterinary conference in the UK. PLoS ONE.

[137] Burstiner L.C., Faires M., Weese J.S. (2010). Methicillin-resistant *Staphylococcus aureus* colonization in personnel attending a veterinary surgery conference. Vet. Surg..

[138] Paul N.C., Moodley A., Ghibaudo G., Guardabassi L. (2011). Carriage of methicillin-resistant *Staphylococcus pseudintermedius* in small animal veterinarians: Indirect evidence of zoonotic transmission: Methicillin-resistant *Staphylococcus pseudintermedius* in Veterinarians. Zoonoses Public Health.

[139] Boost M.V., So S.Y.C., Perreten V. (2011). Low rate of methicillin-resistant coagulase-positive staphylococcal colonization of veterinary personnel in Hong Kong: Staphylococcal carriage in vets. Zoonoses Public Health.

[140] Malissiova E., Chasioti M., Papadopoulos T., Komodromos D., Hadjichristodoulou C., Sergelidis D. (2018). Nasal carriage and antimicrobial susceptibility of coagulase–negative staphylococci (CoNS) among healthy veterinary students in Greece. J. Hell. Vet. Med. Soc..

[141] Rasamiravaka T., Andriatsitohanana T.T., Rasamindrakotroka A. (2017). Evaluation of methicillin-resistant *Staphylococcus aureus* nasal carriage in Malagasy pig and poultry non-industrial farmers. J. Infect. Dev. Ctries..

[142] Mourabit N., Arakrak A., Bakkali M., Zian Z., Bakkach J., Laglaoui A. (2020). Nasal carriage of *Staphylococcus aureus* in farm animals and breeders in North of Morocco. BMC Infect. Dis..

[143] Elemo K.K., Sisay T., Shiferaw A., Fato M.A. (2017). Prevalence, risk factors and multidrug resistance profile of *Staphylococcus aureus* isolated from bovine mastitis in selected dairy farms in and around Asella town, Arsi Zone, South Eastern Ethiopia. Afr. J. Microbiol. Res..

[144] Adesida S.A., Oke A.O., Amosun E.A., Coker A.O. (2019). Nasal Carriage of Methicillin resistant *Staphylococcus aureus* in livestock and farm workers in two com-munities in Lagos, Nigeria. Israel J. Vet. Med..

[145] Okorie-Kanu O.J., Anyanwu M.U., Ezenduka E.V., Mgbeahuruike A.C., Thapaliya D., Gerbig G., Ugwuijem E.E., Okorie-Kanu C.O., Agbowo P., Olo-runleke S. (2020). Molecular epidemiology, genetic diversity and antimicrobial resistance of *Staphylococcus aureus* isolated from chicken and pig carcasses, and carcass handlers. PLoS ONE.

[146] Odetokun I.A., Ballhausen B., Adetunji V.O., Ghali-Mohammed I., Adelowo M.T., Adetunji S.A., Fetsch A. (2018). Staphylococcus aureus in two municipal abattoirs in Nigeria: Risk perception, spread and public health implications. Vet. Microbiol..

[147] Otalu O.J., Kwaga J.K.P., Okolocha E.C., Islam M.Z., Moodley A. (2018). High genetic similarity of MRSA ST88 isolated from pigs and humans in Kogi State, Nigeria. Front. Microbiol..

[148] Kalayu A.A., Woldetsadik D.A., Woldeamanuel Y., Wang S.-H., Gebreyes W.A., Teferi T. (2020). Burden and antimicrobial resistance of *S. aureus* in dairy farms in Mekelle, Northern Ethiopia. BMC Vet. Res..

[149] Gaddafi M.S., Yakubu Y., Junaidu A.U., Bello M.B., Garba B., Bitrus A.A., Lawal H. (2021). Nasal colonization of pigs and farm attendants by *Staphylococcus aureus* and methicillin-resistant *Staphylococcus aureus* (MRSA) in Kebbi, Northwestern Nigeria. Thai J. Vet. Med..

[150] Rongsanam P., Yano T., Yokart W., Yamsakul P., Sutammeng S., Udpaun R., Pichpol D., Tamdee D., Anukool U. (2020). Acquisition risk factors of the SCC*mec* IX-methicillin-resistant *Staphylococcus aureus* in swine production personnel in Chiang Mai and Lamphun Provinces, Thailand. Antibiotics.

[151] Assafi M.S., Hado H.A., Abdulrahman I.S. (2020). Detection of methicillin-resistant *Staphylococcus aureus* in broiler and broilers farm workers in Duhok, Iraq by using conventional and PCR techniques. Iraqi J. Vet. Sci..

[152] Moon D.C., Jeong S.K., Hyun B.-H., Lim S.-K. (2019). Prevalence and characteristics of methicillin-resistant *Staphylococcus aureus* isolates in pigs and pig farmers in Korea. Foodborne Pathog. Dis..

[153] Garipcin M.G.U., Seker E. (2015). Nasal carriage of methicillin-resistant nasal carriage of methicillin-resistant *Staphylococcus aureus* in cattle and farm workers in Turkey. Vet. Arh..

[154] Cui S., Li J., Hu C., Jin S., Li F., Guo Y., Ran L., Ma Y. (2009). Isolation and characterization of methicillin-resistant *Staphylococcus aureus* from swine and workers in China. J. Antimicrob. Chemother..

[155] Fan Y., Wang X., Li L., Yao Z., Chen S., Ye X. (2016). Potential relationship between phenotypic and molecular characteristics in revealing livestock-associated *Staphylococcus aureus* in Chinese humans without occupational livestock contact. Front. Microbiol..

[156] Ye X., Fan Y., Wang X., Liu W., Yu H., Zhou J., Chen S., Yao Z. (2016). Livestock-associated methicillin and multidrug resistant *S. aureus* in humans is associated with occupational pig contact, not pet contact. Sci. Rep..

[157] Fang H.-W., Chiang P.-H., Huang Y.-C. (2014). Livestock-associated methicillin-resistant *Staphylococcus aureus* ST9 in pigs and related personnel in Taiwan. PLoS ONE.

[158] Lim S.K., Nam H.M., Jang G.C., Lee H.S., Jung S.C., Kim T.S. (2013). Transmission and persistence of methicillin-resistant *Staphylococcus* aureus in milk, environment, and workers in dairy cattle farms. Foodborne Pathog. Dis..

[159] Back S.H., Eom H.S., Lee H.H., Lee G.Y., Park K.T., Yang S.J. (2020). Livestock-associated methicillin-resistant *Staphylococcus aureus* in Korea: Antimicrobial resistance and molecular characteristics of LA-MRSA strains isolated from pigs, pig farmers, and farm environment. J. Vet. Sci..

[160] Jayaweera J.A.A.S., Kumbukgolla W.W. (2017). Antibiotic resistance patterns of methicillin-resistant *Staphylococcus aureus* (MRSA) isolated from livestock and associated farmers in Anuradhapura, Sri Lanka. Germs.

[161] Normanno G., Dambrosio A., Lorusso V., Samoilis G., Di Taranto P., Parisi A. (2015). Methicillin-resistant *Staphylococcus aureus* (MRSA) in slaughtered pigs and abattoir workers in Italy. Food Microbiol..

[162] Aubry-Damon H., Grenet K., Sall-Ndiaye P., Che D., Cordeiro E., Bougnoux M.-E., Rigaud E., Le Strat Y., Lemanissier V., Armand-Lefèvre L. (2004). Antimicrobial resistance in commensal flora of pig farmers. Emerg. Infect. Dis..

[163] Etter D., Corti S., Spirig S., Cernela N., Stephan R., Johler S. (2020). *Staphylococcus aureus* population structure and genomic profiles in asymptomatic carriers in Switzerland. Front. Microbiol..

[164] van Cleef B.A., Broens E.M., Voss A., Huijsdens X.W., Zuchner L., Van Benthem B.H., Kluytmans J.A.J.W., Mulders M.N., Van De Giessen A.W. (2010). High prevalence of nasal MRSA carriage in slaughterhouse workers in contact with live pigs in The Netherlands. Epidemiol. Infect..

[165] Van Cleef B.A.G.L., VAN Benthem B.H.B., Verkade E.J.M., VAN Rijen M.M.L., Kluytmans-VAN DEN Bergh M.F.Q., Graveland H., Bosch T., Verstappen K.M.H.W., Wagenaar J.A., Heederik D. (2016). Health and health-related quality of life in pig farmers carrying livestock-associated methicillin-resistant *Staphylococcus aureus*. Epidemiol Infect..

[166] Papadopoulos P., Papadopoulos T., Angelidis A.S., Kotzamanidis C., Zdragas A., Papa A., Filioussis G., Sergelidis D. (2019). Prevalence, antimicrobial susceptibility and characterization of *Staphylococcus aureus* and methicillin-resistant *Staphylococcus aureus* isolated from dairy industries in North-Central and North-Eastern Greece. Int. J. Food Microbiol..

[167] Parisi A., Caruso M., Normanno G., Latorre L., Miccolupo A., Fraccalvieri R., Intini F., Manginelli T., Santagada G. (2019). MRSA in swine, farmers and abattoir workers in southern Italy. Food Microbiol..

[168] Sakwinska O., Giddey M., Moreillon M., Morisset D., Waldvogel A., Moreillon P. (2011). *Staphylococcus aureus* host range and human-bovine host shift. Appl. Environ. Microbiol..

[169] Antoci E., Pinzone M.R., Nunnari G., Stefani S., Cacopardo B. (2013). Prevalence and molecular characteristics of methicillin-resistant *Staphylococcus aureus* (MRSA) among subjects working on bovine dairy farms. Infez. Med..

[170] Van Cleef B., Graveland H., Haenen A., Van de Giessen A., Heederik D., Wagenaar J. (2011). Persistance of livestock-associated methicillin-resistant *Staphylococcus aureus* in field workers after short-term occupation exposure to pigs and veal calves. J. Clin. Microbiol..

[171] Fischer J., Hille K., Ruddat I., Mellmann A., Köck R., Kreienbrock L. (2017). Simultaneous occurrence of MRSA and ESBL-producing *Enterobacteriaceae* on pig farms and in nasal and stool samples from farmers. Vet. Microbiol..

[172] Bunke J., Receveur K., Oeser A.C., Gutsmann I., Schubert S., Podschun R., Zell R., Fickenscher H., Krumbholz A. (2020). Epidemiology of bacteria and viruses in the respiratory tract of humans and domestic pigs. APMIS.

[173] Schnitt A., Lienen T., Wichmann-Schauer H., Cuny C., Tenhagen B.-A. (2020). The Occurrence and distribution of livestock-associated methicillin-resistant *Staphylococcus aureus* ST398 on German dairy farms. J. Dairy Sci..

[174] Dahms C., Hübner N.-O., Cuny C., Kramer A. (2014). Occurrence of methicillin-resistant *Staphylococcus aureus* in farm workers and the livestock environment in Mecklenburg-Western Pomerania, Germany. Acta Vet. Scand..

[175] Köck R., Loth B., Köksal M., Schulte-Wülwer J., Harlizius J., Friedrich A.W. (2012). Persistence of nasal colonization with livestock-associated methicillin-resistant *Staphylococcus aureus* in pig farmers after holidays from pig exposure. Appl. Environ. Microbiol..

[176] Bisdorff B., Scholhölter J.L., Claußen K., Pulz M., Nowak D., Radon K. (2012). MRSA-ST398 in livestock farmers and neighbouring residents in a rural area in Germany. Epidemiol. Infect..

[177] Giovanni N., Elisa S., Marta C., Rosa F., Loredana C., Alessandra B., Antonio P. (2020). Occurrence and characteristics of methicillin-resistant *Staphylococcus aureus* (MRSA) in buffalo bulk tank milk and the farm workers in Italy. Food Microbiol..

[178] Pirolo M., Visaggio D., Gioffrè A., Artuso I., Gherardi M., Pavia G., Samele P., Ciambrone L., Di Natale R., Spatari G. (2019). Unidirectional animal-to-human transmission of methicillin-resistant *Staphylococcus aureus* ST398 in pig farming; evidence from a surveillance study in Southern Italy. Antimicrob. Resist. Infect. Control.

[179] Mascaro V., Squillace L., Nobile C.G., Papadopoli R., Bosch T., Schouls L.M., Casalinuovo F., Musarella R., Pavia M. (2019). Prevalence of methicillin-resistant *Staphylococcus aureus* (MRSA) carriage and pattern of antibiotic resistance among sheep farmers from Southern Italy. Infect. Drug Resist..

[180] Graveland H., Wagenaar J.A., Heesterbeek H., Mevius D., van Duijkeren E., Heederik D. (2010). Methicillin resistant *Staphylococcus aureus* ST398 in veal calf farming: Human MRSA carriage related with animal antimicrobial usage and farm hygiene. PLoS ONE.

[181] Reynaga E., Navarro M., Vilamala A., Roure P., Quintana M., Garcia-Nuñez M., Figueras R., Torres C., Lucchetti G., Sabrià M. (2016). Prevalence of colonization by methicillin-resistant *Staphylococcus aureus* ST398 in pigs and pig farm workers in an area of Catalonia, Spain. BMC Infect. Dis..

[182] Neyra R.C., Frisancho J.A., Rinsky J.L., Resnick C., Carroll K.C., Rule A.M., Ross T., You Y., Price L.B., Silbergeld E.K. (2014). Multidrug-resistant and methicillin-resistant *Staphylococcus aureus* (MRSA) in hog slaughter and processing plant workers and their community in North Carolina (USA). Environ. Health Perspect..

[183] Hatcher S.M., Rhodes S.M., Stewart J.R., Silbergeld E., Pisanic N., Larsen J., Jiang S., Krosche A., Hall D., Carroll K.C. (2017). The prevalence of antibiotic-resistant *Staphylococcus aureus* nasal carriage among industrial hog operation workers, community residents, and children living in their households: North Carolina, USA. Environ. Health Perspect..

[184] Rinsky J.L., Nadimpalli M., Wing S., Hall D., Baron D., Price L.B., Larsen J., Stegger M., Stewart J., Heaney C.D. (2013). Livestock-associated methicillin and multidrug resistant *Staphylococcus aureus* is present among industrial, not antibiotic-free livestock operation workers in North Carolina. PLoS ONE.

[185] Smith T.C., Gebreyes W.A., Abley M.J., Harper A.L., Forshey B.M., Male M.J., Martin H.W., Molla B.Z., Sreevatsan S., Thakur S. (2013). Methicillin-resistant *Staphylococcus aureus* in pigs and farm workers on conventional and antibiotic-free swine farms in the USA. PLoS ONE.

[186] Khanna T., Friendship R., Dewey C., Weese J.S. (2008). Methicillin resistant *Staphylococcus aureus* colonization in pigs and pig farmers. Vet. Microbiol..

[187] Sahibzada S., Hernández-Jover M., Jordan D., Thomson P.C., Heller J. (2018). Emergence of highly prevalent CA-MRSA ST93 as an occupational risk in people working on a pig farm in Australia. PLoS ONE.

[188] Sinlapasorn S., Lulitanond A., Angkititrakul S., Chanawong A., Wilailuckana C., Tavichakorntrakool R., Chindawong K., Seelaget C., Krasaesom M., Chartchai S. (2015). SCCmec IX in Meticillin-resistant *Staphylococcus aureus* and meticillin-resistant coagulase-negative Staphylococci from pigs and workers at pig farms in Khon Kaen, Thailand. J. Med. Microbiol..

[189] Founou L.L., Founou R.C., Essack S.Y., Djoko C.F. (2018). Mannitol-fermenting methicillin-resistant staphylococci (MRS) in pig abattoirs in Cameroon and South Africa: A serious food safety threat. Int. J. Food Microbiol..

[190] Argudín M.A., Vanderhaeghen W., Vandendriessche S., Vandecandelaere I., André F.-X., Denis O., Coenye T., Butaye P. (2015). Antimicrobial resistance and population structure of *Staphylococcus epidermidis* recovered from animals and humans. Vet. Microbiol..

[191] Roberts M.C., Garland-Lewis G., Trufan S., Meschke S.J., Fowler H., Shean R.C., Greninger A.L., Rabinowitz P.M. (2018). Distribution of *Staphylococcus* species in dairy cows, workers and shared farm environments. FEMS Microbiol. Lett..

[192] Bogaert D., van Belkum A., Sluijter M., Luijendijk A., de Groot R., Rümke H.C., Verbrugh H.A., Hermans P.W.M. (2004). Colonisation by *Streptococcus pneumoniae* and *Staphylococcus aureus* in Healthy Children. Lancet.

[193] Lebon A., Labout J.A.M., Verbrugh H.A., Jaddoe V.W.V., Hofman A., van Wamel W., Moll H.A., van Belkum A. (2010). Dynamics and determinants of *Staphylococcus aureus* carriage in infancy: The generation R study. J. Clin. Microbiol..

[194] Mollema F.P.N., Richardus J.H., Behrendt M., Vaessen N., Lodder W., Hendriks W. (2010). Transmission of methicillin-resistant *Staphylococcus aureus* to household contacts. J Clin. Microbiol..

[195] Fall C., Seck A., Richard V., Ndour M., Sembene M., Laurent F., Breurec S. (2012). Epidemiology of *Staphylococcus aureus* in Pigs and farmers in the largest farm in Dakar, Senegal. Foodborne Pathog. Dis..

[196] Lee A., de Lencastre H., Garau J. (2018). Methicillin-resistant *Staphylococcus aureus*. Nat. Rev. Dis. Primers.

[197] Vrieling M., Tuffs S.W., Yebra G., van Smoorenburg M.Y., Alves J., Pickering A.C., Park J.Y., Park N., Heinrichs D.E., Benedictus L. (2020). Population analysis of Staphylococcus aureus reveals a cryptic, highly prevalent superantigen SElW that contributes to the pathogenesis of bacteremia. mBio.

[198] Cuny C., Abdelbary M.M.H., Köck R., Layer F., Scheidemann W., Werner G., Witte W. (2016). Methicillin-Resistant Staphylococcus aureus from infections in horses in germany are frequent colonizers of veterinarians but rare among MRSA from infections in humans. One Health.

[199] Cheung G.Y.C., Bae J.S., Otto M. (2021). Pathogenicity and Virulence of Staphylococcus aureus. Virulence.

[200] Seilie E.S., Bubeck Wardenburg J. (2017). Staphylococcus aureus pore-forming toxins: The interface of pathogen and host complexity. Semin. Cell Dev. Biol..

[201] Kong C., Neoh H.-M., Nathan S. (2016). Targeting Staphylococcus aureus toxins: A potential form of anti-virulence therapy. Toxins.

[202] Ballhausen B., Kriegeskorte A., van Alen S., Jung P., Köck R., Peters G., Bischoff M., Becker K. (2017). The pathogenicity and host adaptation of Livestock-Associated MRSA CC398. Vet. Microbiol..

[203] Fisher E.L., Otto M., Cheung G.Y.C. (2018). Basis of virulence in enterotoxin-mediated staphylococcal food poisoning. Front. Microbiol..

[204] Richardson E.J., Bacigalupe R., Harrison E.M., Weinert L.A., Lycett S., Vrieling M., Robb K., Hoskisson P.A., Holden M.T.G., Feil E.J. (2018). Gene exchange drives the ecological success of a multi-host bacterial pathogen. Nat. Ecol. Evol..

[205] Mama O.M., Aspiroz C., Ruiz-Ripa L., Torres C. (2020). Relevance of clonal complex CC398 in bacteremia caused by *Staphylococcus aureus* in a secondary hospital of Aragon, Spain. Enferm. Infec. Microbiol. Clin. (English ed.).

[206] Kim H.J., Jo A., Jeon Y.J., An S., Lee K.-M., Yoon S.S., Choi J.Y. (2019). Nasal commensal *Staphylococcus epidermidis* enhances interferon-λ-dependent immunity against Influenza virus. Microbiome.

[207] Dziri R., Klibi N., Lozano C., Ben Said L., Bellaaj R., Tenorio C., Boudabous A., Ben Slama K., Torres C. (2016). High prevalence of *Staphylococcus haemolyticus* and *Staphylococcus saprophyticus* in environmental samples of a Tunisian Hospital. Diagn. Microbiol. Infect. Dis..

[208] Mama O.M., Dieng M., Hanne B., Ruiz-Ripa L., Diop C.G.M., Torres C. (2019). Genetic characterisation of staphylococci of food-producing animals in Senegal. PVL detection among MSSA. BMC Vet. Res..

[209] Lynch S.A., Helbig K.J. (2021). The complex diseases of *Staphylococcus pseudintermedius* in canines: Where to next?. Vet. Sci..

[210] Paul N.C., Bärgman S.C., Moodley A., Nielsen S.S., Guardabassi L. (2012). *Staphylococcus pseudintermedius* colonization patterns and strain diversity in healthy dogs: A cross-sectional and longitudinal study. Vet. Microbiol..

[211] Iverson S.A., Brazil A.M., Ferguson J.M., Nelson K., Lautenbach E., Rankin S.C., Morris D.O., Davis M.F. (2015). Anatomical patterns of colonization of pets with Staphylococcal species in homes of people with methicillin-resistant *Staphylococcus aureus* (MRSA) skin or soft tissue infection (SSTI). Vet. Microbiol..

[212] Silva V., Oliveira A., Manageiro V., Caniça M., Contente D., Capita R., Alonso-Calleja C., Carvalho I., Capelo J.L., Igrejas G. (2021). Clonal diversity and antimicrobial resistance of methicillin-resistant *Staphylococcus pseudintermedius* isolated from canine pyoderma. Microorganisms.

[213] Perkins A.V., Sellon D.C., Gay J.M., Lofgren E.T., Moore D.A., Jones L.P., Davis M.A. (2020). Prevalence of methicillin-resistant *Staphylococcus pseudintermedius* on hand-contact and animal-contact surfaces in companion animal community hospitals. Can. Vet. J..

