# Supplementary material for: Ecology and Genetic Lineages of Nasal Staphylococcus aureus and MRSA Carriage in Healthy Persons with or without Animal-Related Occupational Risks of Colonization: A Review of Global Reports
Source: Pathogens. 2021 Aug 8;10(8):1000. doi: 10.3390/pathogens10081000 (PMC8400700; doi:10.3390/pathogens10081000)
Supplement: Supplementary file 1 [file pathogens-10-01000-s001.zip › SUPPLEMENTARY FILE-POST PUBLICATION-corrected.pdf]

SUPPLEMENTARY FILE

Supplementary Table S1: Study characteristics, species, antimicrobial resistance and virulence genes of Staphylococcal nasal carriages in healthy humans that did not report any risk factor of *S. aureus* colonization (n = 58)

Author (s) (reference)	Staphylococcal species	Continent	Country/Subjects	Study design	Sample size	No. of staphylococcal positive cases (%)	Detection method[a]	Detection of *S. aureus* (%)/ MRSA (%)	*Staphylococcus* species (non-*aureus*) detected (%)[b]	Antimicrobial resistance phenotype technique/ Major resistant types[c]	Antimicrobial resistance genes	Virulence gene
Elsanousi and Elsanousi [29]	*S. aureus*	Africa	Sudan/ Healthy people	Cross-sectional	220	180 (81.8)	C	23 (10.5)/NT	NR	NT	NT	NT
Ogefere and Ogunleye [30]	*S. aureus*	Africa	Nigeria /Healthy students	Cross-sectional	400	229 (57.3)	C/PCR	229 (57.3)/ 23 (5.8)	NT	DD<br>FOX, OXA, ERY, GEN,	*mecA*	NT
Onanuga and Temedie [31]	*S. aureus*	Africa	Nigeria /Healthy community residents	Cross-sectional	120	40 (33.3)	C	40 (33.3)/ 21 (17.5)	NA	DD<br>OXA, AMP, ERY, DOX, CIP, VAN	NT	NT
Nsofor *et al* [32]	*S. aureus*	Africa	Nigeria/Healthy school children	Cross-sectional	270	152 (56.3)	C	152 (56.3)/ NT	NA	NT	NT	NT
Anueyiagu *et al* [20]	*S. aureus*	Africa	Nigeria/ Healthy students	Prospective	130	83 (51.9)	C	85 (51.9)/ 2 (1.5)	NA	DD<br>OXA, ERY, GEN, STR, CIP, CHL	NT	NT
Eibach *et al* [33]	*S. aureus*	Africa	Ghana/Healthy children	Cross-sectional	544	120/ (22.1)	C/ PCR/ MT	120/ (22.1)/ 2 (0.37)	NT	FOX: DD<br>PEN, TET, CLI, ERY, GEN, SXT,	*mecA*	*lukS/F-PV*, TSST-1
Egyi *et al* [34]	*S. aureus*	Africa	Ghana/ Healthy people	Cross-sectional	234	49 (21.0)	C/PCR	49 (21.0)/ 2 (0.3)	NT	DD<br>PEN, FOX, TET, ERY	*mecA*	*lukS/F-PV*
Ngoa *et al* [35]	*S. aureus*	Africa	Gabon/Healthy community	Cross-sectional	205	60 (29.2)	C/PCR/MT	60 (29.2)/ 1 (0.49)	NT	DD<br>PEN, ERY, CLO, CLI, TET	*mecA*	NT
Mourabit *et al* [36]	*S. aureus*	Africa	Morocco/Healthy Volunteers	Cross-sectional	465	178 (38.3)	C / MALDI / PCR	178 (38.3)/ 21 (4.5)	NA	DD<br>FOX, OXA, GEN, ERY, TET.	*mecA*	ND

Tigabu *et al* [37]	*S. aureus*	Africa	Ethiopia/ Healthy Primary and High School students	Cross-sectional	640	143 (22.3)	C	143 (22.3/ 14 (2.2)	NA	DD<br>FOX, ERY, TET, CIP, GEN	NT	NT
Ebruke *et al* [38]	*S. aureus*	Africa	Gambia/ Healthy people	Cross-sectional	600	151 (25.2)	C/PCR/MT	151 (25.2)/ 0 (0.0)	NT	DD<br>SXT, TET	ND	NT
El-Mahdy *et al* [39]	*S. aureus*	Asia	Saudi Arabia/Healthy adults	Prospective	70	26 (37.1)	C / PCR / MT	26 (37.1)/ 1 (1.4)	NA	DD<br>AMP, AMC, ERY	*mecA*	NT
Al-Haj *et al* [40]	*S. aureus*	Asia	Yemen/Healthy students	Cross-sectional	450	104 (23.1)	C	104 (23.1)/NR	NA	DD<br>AMX, ERY, CIP, CHL, GEN	NA	NA
Soltani *et al* [41]	*S. aureus*	Asia	Iran/Healthy Children	Cross-sectional	350	92 (26.2)	C	92 (26.2)/ 33 (9.4)	NA	DD<br>OXA, CIP, CLI, VAN	NT	NT
Khorvash *et al* [42]	*S. aureus*	Asia	Iran/Healthy Adult	Cross-sectional	158	42 (26.6)	C	42 (26.6)/ 5 (11.9)	NA	DD<br>PEN, OXA, TET, ERY, DOX, RIF	NT	NT
Mobasherizadeh *et al* [43].	*S. aureus*	Asia	Iran/Healthy children	Cross-sectional	410	115 (28.0)	C/PCR	115 (28.0)/25 (6.1)	NA	DD<br>FOX, ERY, PEN, TET, CLI, CIP	*mecA*	*eap*
Al-Shami and Al-Shammari [44]	*S. aureus*	Asia	Iraq/ Healthy students	Prospective	71	15 (21.1)	C	15 (21.1)/ 11(15.5)	NA	DD<br>PEN, FOX, ERY, VAN	NT	NT
Rasheed and Hussain [45]	*S. aureus*	Asia	Iraq/Heathy refugees and host communities	Comparative Cross-sectional	492 heathy and 355 host community members	NR	C / PCR	NR/76 (15.4) and NR/49 (13.8) refugees and community, respectively	NA	DD<br>GEN, ERY, CIP, STR, CHL	*mecA*: 98.7% in host community and 85.7% in refugees	NT
Suvarnsit *et al* [46]	*S. aureus*	Asia	Thailand/ Healthy volunteers	Prospective	100	20 (20.0)	C	20 (20.0)/0 (0.0)	NA	DD<br>OXA	NT	NT
Chen *et al* [47]	*S. aureus*	Asia	China/ Healthy community	Cross-sectional	295	73 (24.7)	C / PCR	73 (24.7)/ 1 (0.3)	NA	DD<br>FOX	*mecA*	*sea, seb*

Gong *et al* [48]	*S. aureus*	Asia	China/ Healthy children	Cross-sectional	314	16 (5.10)	C/ PCR	16 (5.10) / 3 (0.95)	NT	DD OXA, CLI, CIP	*mecA*	*lukS/F-PV*
Yan *et al* [49]	*S. aureus*	Asia	China/Healthy adults	Cohort	2448	403 (16.3)	C/ PCR/ MT	403 (16.3)/8 (0.33)	NA[d]	NT[d]	*mecA*	*lukS/F-PV*
Jamil *et al* [50]	*S. aureus*	Asia	Pakistan/ Healthy people	Cross-sectional	200	41 (20.5)	C	41 (20.5)/ 0 (0.0)	NT	DD PEN, OXA, ERY, CIP, SXT	NT	NT
Chatterjee *et al* [51]	*S. aureus*	Asia	India/ healthy children	Cross-sectional	489	C/PCR/MT	256 (52.5)	256 (52.5)/16 (3.27)	NT	DD OXA, GEN, CLI, ERY	*mecA*	*NT*
Fomda *et al* [52]	S. aureus	Asia	India/ healthy people	Cross-sectional	820	C/PCR	229 (27.92)	229 (27.92) /15 (1.8)	NT	DD PEN, AMP, OXA, FOX, CIP, SXT, DOX, CHL, TET	*mecA*	NT
Choi *et al* [53]	*S. aureus*	Asia	Malaysia/ Healthy adults	Cross-sectional	346	81 (23.4)	C	81 (23.4)/ 1 (0.29)	NT	DD PEN, OXA, ERY, TET, GEN	NT	NT
Anbazhagan *et al* [54]	*S. aureus*	Asia	Malaysia/ Healthy students	Cross-sectional	147	117 (79.6)	C	117 (79.6)/ 21 (13.9)	NA	DD OXA	NA	NA
Wang *et al* [55]	*S. aureus*	Asia	Taiwan/ healthy students	Cross-sectional	259	58 (22.4)	C/PCR/MT	58 (22.4) / 4 (1.5)	NT	DD OXA	*mecA*	ND
Dinić *et al* [56]	*S. aureus*	Europe	Serbia / healthy Adults & children	Cohort	58908	1381 (2.34)	C	1381 (2.34)/ 123 (0.21)	NA	DD PEN, FOX, ERY, CLI	NT	NT
Ritchie *et al* [57]	*S. aureus*	Europe	Germany/ healthy students	Prospective	122	69 (57.0)	C / MT	69 (57.0)/ NT	NA	NT	NT	NA
Andersen *et al* [58]	*S. aureus*	Europe	Denmark/ healthy population	Prospective	2196	543 (24.7)	C/PCR	543 (24.7)/ NR	NA	NT	NT	NT
Karapsias *et al* [59]	*S. aureus*	Europe	Greece/ Healthy Air force staff	Cohort	959	MRSA: 9 (0.94)	C/PCR/MT	NR/ 9 (0.94)	NA	NT	*mecA*	*lukS/F-PV*
Kirkliauskienė *et al* [60]	*S. aureus*	Europe	Denmark/Healthy persons	Cross-sectional	537	273 (50.8)	C/PCR	273 (50.8)/ 0 (0.00)	NA	DD PEN, OXA, FOX, TET, CLI, ERY	NT	*lukS/F-PV*

Mehraj *et al* [61]	*S. aureus*	Europe	Germany / healthy people	Cross-sectional	389	85 (21.9)	C /PCR/MT	85 (21.9)/5 (1.3)	NT	DD<br>PEN, OXA, CIP, MFL, ERY, CLI	*mecA*	NT
den Heijer *et al* [62]	*S. aureus*	Europe	Austria/ healthy people	Cross-sectional	3309	549 (16.6)	C /PCR/MT	549 (16.6)/ 8 (0.2)	NT	DD<br>PEN, OXA, CIP, AZT, CLIN, GEN, TET	*mecA*	NT
den Heijer *et al* [62]	*S. aureus*	Europe	Belgium/ healthy people	Cross-sectional	3025	584 (19.3)	C /PCR/MT	584 (19.3)/12 (0.4)	NT	DD<br>PEN, OXA, CIP, AZT, CLIN, GEN, TET	*mecA*	NT
den Heijer *et al* [62]	*S. aureus*	Europe	Croatia/ healthy people	Cross-sectional	3960	792 (20.0)	C /PCR/MT	792 (20.0)/15 (0.4)	NT	DD<br>PEN, OXA, CIP, AZT, CLIN, GEN, TET	*mecA*	NT
den Heijer *et al* [62]	*S. aureus*	Europe	France/ healthy people	Cross-sectional	3858	876 (22.7)	C /PCR/MT	876 (22.7)/16 (0.4)	NT	DD<br>PEN, OXA, CIP, AZT, CLIN, GEN, TET	*mecA*	NT
den Heijer *et al* [62]	*S. aureus*	Europe	Hungary/ healthy people	Cross-sectional	3847	542 (14.1)	C /PCR/MT	542 (14.1)/8 (0.2)	NT	DD<br>PEN, CIP, AZT, CLIN, GEN, TET, OXA	*mecA*	NT
den Heijer *et al* [62]	*S. aureus*	Europe	Netherlands/ healthy people	Cross-sectional	3847	1073 (27.9)	C /PCR/MT	1073 (27.9)/9 (0.2)	NT	DD<br>PEN, CIP, OXA, AZT, CLIN, GEN, TET	*mecA*	NT
den Heijer *et al* [62]	*S. aureus*	Europe	Spain/ healthy people	Cross-sectional	3990	770 (19.3)	C /PCR/MT	770 (19.3)/10 (0.3)	NT	DD<br>PEN, CIP, OXA,AZT, CLIN, GEN, TET	*mecA*	NT
den Heijer *et al* [62]	*S. aureus*	Europe	Sweden/ healthy people	Cross-sectional	3214	958 (29.8)	C /PCR/MT	958 (29.8)/ 0 (0.0)	NT	DD<br>PEN, CIP, AZT, CLIN,GEN, TET	ND	NT
den Heijer *et al* [62]	*S. aureus*	Europe	UK/ healthy people	Cross-sectional	3156	814 (25.8)	C /PCR/MT	814 (25.8)/13 (0.4)	NT	DD<br>PEN, CIP, OXA,AZT, CLIN, GEN, TET	*mecA*	NT
Becker *et al* [63]	*S. aureus*	Europe	Germany/ healthy people	Cross-sectional	1878	C/PCR/MT	920 (40.9)	920 (40.9)/15 (0.8)	NT	DD	*mecA*	*tst*, *sec*, *seg*, *sei*, *lukFS*

										FOX, LEV, TET, SXT, CLI, ERY, TET		
Netsvyetayeva *et al* [64]	*S. aureus*	Europe	Ukraine/ healthy people	Cross-sectional	245	C/PCR/MT	99 (40.4)	99 (40.4)/ 9 (3.7)		DD<br>FOX, GEN, SXT, TET, CIP, RIF, ERY, CLI	*mecA*	l*ukPV eta etb etd fnbA*
Falomir *et al* [65]	*S. aureus*	Europe	Spain/Healthy university students	Cross-sectional	203	45 (22.2)	C	45 (22.2)/0 (0.0)	NT	DD<br>FOX	NT	NT
Tiera *et al* [66]	*S. aureus*	Europe	Spain/Healthy students	Cross-sectional	102	14 (13.7)	C	14 (13.7)/ 6 (5.9)	NT	DD<br>OXA	NT	NT
Lozano *et al* [67]	*S. aureus*	Europe	Spain/Healthy persons	Cross-sectional	278	53 (19.1)	C/PCR/ MT	53 (19.1)/ 1 (0.36)	NA	DD<br>PEN, OXA, FOX, CIP, ERY	*mecA*, *msr*(A), *erm*(A), *erm*(C), erm(T)	*eta, hla, hld, hlg-2, hlg, seb, tst-1, sea, sep*
Muthukrishnan *et al* [68]	*S. aureus*	America	USA/Healthy persons	Prospective	109	61 (55.9)	C / MT	61 (55.9)/ 7 (11.5)	NA	DD<br>FOX	*mecA*	NT
Velasco *et al* [69]	*S. aureus*	America	USA/Healthy people	Cross-sectional	550	42 (7.6)	C/PCR/MT	42 (7.6)/ 0 (0.0)	NA	DD<br>PEN, TET, ERY, CIP, KAN,	ND	ND
Kuehnert *et al* [70]	*S. aureus*	America	USA/Healthy people	Cross-sectional	9622	3117 (32.4)	C/PCR	3117 (32.4)/77 (0.8)	NT	DD<br>PEN, OXA, TET, ERY, LEV	*mecA*	S*ea, seb, sec, sed, seg, seh*, TSST-1, *lukS/F-PV*
Gorwitz *et al* [71]	*S. aureus*	America	USA/Healthy people	Cross-sectional	9004	2575 (28.6)	C/PCR	2575 (28.6) /135 (1.5)	NT	NT	*mecA*	*lukS/F-PV*
Wardyn *et al* [72]	*S. aureus*	America	USA/Healthy community members	Cohort	1342	156 (22.5)	C/PCR/MT	156 (22.5)/ 19 (2.7)	NA	DD<br>OXA, ERY, TET, CLI	*mecA*	*lukS/F-PV*
Wardyn *et al* [73]	*S. aureus*	America	USA/Healthy rural community	Cross-sectional	131	31 (23.7)	C/ PBP2a Immnoagglutination /PCR/MT	31 (23.7)/ 0 (0.0)	NA	DD<br>PEN, ERY, SXT, LEV, CLI	ND	*lukS/F-PV*
Pires *et al*. [74]	*S. aureus*	America	Brazil/Healthy community	Cross-sectional	686	224 (32.7)	C /PCR / MT	224 (32.7)/ 6 (0.9)	NA	DD<br>FOX	All MRSA harbored *mecA*	NA
Braga *et al* [75]	*S. aureus*	America	Barzil/ healthy children	Cross-sectional	500	C/PCR	240 (48.0)	240 (48.0)/ 31 (6.2)	NT	DD<br>OXA, FOX	*mecA*	NT
Hamdan-Partida *et al* [76]	*S. aureus*	America	Mexico/ healthy people	Cross-sectional	1243	C/PCR	296 (23.9)	296 (23.9)/ 48 (3.9)	NT	DD<br>FOX, PEN, ERY, CLI, TET, CIP, SXT	*mecA*	NT

Rebollo-P ́érez *et al* [77]	*S. aureus*	America	Colombia/Healthy preschool children	Cross-sectional	104	40 (38.5)	C / PCR / MT	40 (38.5)/ 5 (4.8)	NA	DD<br><br>FOX, ERY, CLI, GEN, RIF	*mecA*	NT
Hanselman *et al* [78]	*S. aureus*	America	Canada/Healthy school teachers	Prospective	220	NR	C/PCR/PBP2a immunoagglutination/MT	79 (35.9) /7 (3.2)	NA	DD<br><br>OXA	NT	*lukS/F-PV*
Gardella *et al* [79]	*S. aureus*	America	Argentina/Healthy children	Cross-sectional	316	98 (31.0)	C/PCR/MT	98 (31.0)/ 14 (4.4)	NT	DD<br><br>ERY, CLI, RIF	*mecA*	*lukS/F-PV* and sea
Munckhof *et al* [80]	*S. aureus*	Australia	Australia/ Healthy volunteers	Prospective	303	96 (31.7)	C / PCR	96 (31.7)/0 (0.0)	NA	DD<br>OXA	ND	NT
Ogefere *et al* [81]	CoPS and CoNS	Africa	Nigeria /Healthy students	Cross-sectional	350	220 (62.9)	C	148 (42.3)/ NT	NR	NT	NT	NT
Abadi *et al* [82]	CoPS and CoNS	Asia	Iran/Healthy school students	Cross-sectional	600	539 (89.8)	C / PCR	109 (18.2)/ 46 (7.7)	SL (29), SHae(24) SSa(23.6), SE (12.5), SSli(11.1)<br><br>MR-isolates: 72 (16.7) MRCoNS were identified. SSa had the highest MR rate.	DD<br><br>FOX	*mecA*	NT
Falomir *et al* [83]	CoPS and CoNS	Europe	Spain/Healthy older persons (average 63.7 years)	Cross-sectional	27	4 (15.0)	C	4 (15.0)/ 1 (3.7)	SW, Spa, SKi , Sxy	DD<br><br>(*S. aureus)*<br>PEN, OXA CLI<br><br>(CoNS)<br>PEN, OXA, TET, CLA, RIF, CIP	NT	NT
Falomir *et al* [84]	CoPS and CoNS	Europe	Spain/Healthy university students	Cross-sectional	445	99 (22.2)	C	99 (22.2)/6 (1.3)	Sin, SSa, SCa, SKl, SW.	DD<br><br>OXA, ERY, MUP	*NT*	NT
Lebeaux *et al* [85]	CoNS	Europe	France / Healthy community	Cross-sectional	154	72 (46.7)	C/PCR/MT	34 (21.5)/ 2 (1.29)	SE (21.4), Shae (27), SH (18.3), SSa (16.2)	NT	*mecA* in MRSA	NT

[a] Detection method: C/MALDI/PCR/MT: Culture / MALDI-TOF-MS / PCR / Molecular typing
[b] SE*: S. epidermidis;* SHae*: S. haemolyticus;* SSa*: S. saprophyticus;* SL*: S. lugdunensis*; SCa: *S. capitis*; SC: *S. cohini:* SH: *S. hominis*; SHy: *S. hyicus;* SCs: *S. caseolyticus;* SSi: *S. simulans*; SD; *S. delphini;* SSli: S. *schleiferi* SW: *S. warnieri*; SPas: *S. pasteuri.*
[c]Technique used: Disk-diffusion method (DD-method), MIC determination (MIC). Top 4 groups of antimicrobial agents: beta-lactams (BL). Fluoroquinolones (FQ), Macrolides (Mc), Tetracycline (Tet), Vancomycin (Van)
[d]NT: Not tested
NA: Not available (tested but result not published on article)
ND: Not detected
NR: Not reported in detail

PEN: Penicillin; AMX: Amoxicillin; ERY: Erythromycin; CHL: Chloramphenicol; CLI: Clindamycin; STR: Streptomycin; Van: vancomycin; TET: Tetracycline; OXA: Oxacillin; RIF: Rifampicin, FOX: Cefoxitin; LEV: Levofloxacin; CIP: Ciprofloxacin;; KAN: Kanamycin; GEN; Gentamycin; MUP: Mupirocin; SXT: Trimethoprim/sulfamethoxazole

Supplementary Table S2. Study characteristics, species, antimicrobial resistance pattern and virulence genes detected in staphylococcal nasal isolates recovered from healthy food-handlers (n = 31).

Author (s) (reference)	Staphylococcal species	Continent	Country/ Subjects	Study design	Sample size	No. of Staphylococcal Positive Cases (%)	Detection method C/MALDI/PCR/ MT[a]	Detection of *S. aureus* (%)/ MRSA (%)	*Staphylococcus species* (non-*aureus*) detected (%)[b]	Antimicrobial Resistance phenotype Technique/ Major resistant types[c]	Antimicrobial Resistance genes	Virulence genes
El-Shenawy *et al* [86]	*S. aureus*	Africa	Egypt / heathy Food handlers	Cross-sectional	200	61 (30.5)	C /STE- Reverse Passive Latex Agglutination (RPLA)	23 (10.5)/NT	NA	NT	NA	*sea, seb, sec, sed*
Dagnew *et al* [87]	*S. aureus*	Africa	Ethiopia/ Food handers	Cross-sectional	200	41 (20.5)	C	41 (20.5) /NT	NT	NT	NT	NT
Beyene *et al* [88]	*S. aureus*	Africa	Ethiopia/ food handlers	Cross-sectional	300	52 (17.3)	C	52 (17.3) /4 (1.3)	NT	DD<br>OXA	NT	NT
Ahmad [89]	*S. aureus*	Africa	Sudan/ Food Handlers	Cross-sectional	372	93 (25.0)	C/PCR	93 (25) / 39 (10.2)	NT	NT	NT	*sea, seb, sec, sed*
Al Hassan *et al* [90]	*S. aureus*	Africa	Sudan/ University Cafeterias food-handers	Cross-sectional	80	16 (20)	C	16 (20.0) / 0 (0.0)	NT	DD<br>OXA	NT	NT
Emeakaroha *et al* [91]	*S. aureus*	Africa	Nigeria / food handlers	Cross-sectional	54	21 (38.9)	C	21 (38.9) / NT	NT	DD<br>PEN, TET, ERY, CIP	NT	NT
Omololu-Aso *et al* [92]	*S. aureus*	Africa	Nigeria/ Food-handlers	Cross-sectional	35	13 (37.1)	C	13 (37.1) / 13 (37.1)	NT	DD method<br>OXA	NT	NT
Eke *et al* [93]	*S. aureus*	Africa	Nigeria/ Food handlers and restaurant workers	Cross-sectional	100	60 (60.0)	C	60 (60.0)/ NT	NT	DD<br>PEN, CIP, GEN, COT, ERY	NT	NT
Abdulrahman and Taher [94]	*S. aureus*	Asia	Iraq/Food handlers (fast food)	Cross-sectional	200	53 (26.5)	C/PCR	53 (26.5) /NT	NA	NT	ND	NT
Mohammed and Ali [95]	*S. aureus*	Asia	Iraq/ Restaura	Cross-sectional	100	30 (30.0)	C	30 (30.0) / 16 (16.0)	NT	DD	NT	NT

			nt workers							OXA, FOX, CIP, GEN, TET, CLI		
Fooladvand *et al* [96]	*S. aureus*	Asia	Iran. Food handlers	Cross-sectional	1113	C/PCR	224 (20.1)	224 (20.1)/ 37 (3.3)	NT	NT	mecA	ssei, seg, sea, seb, sec, seh
Osman *et al* [97]	*S. aureus*	Asia	Lebanon / healthy Food handlers	Cross-sectional	160	38 (23.8)	C / Maldi / PCR	38 (23.8)/ 5 (3.1)	NA	DD<br>FOX	NT	*sea, sec, sed, etb*
Boost *et al* [98]	*S. aureus*	Asia	Hong Kong/ Pork Butchers	Cross-sectional	300	NR	C/PCR/MT	NR/17 (5.6)	NT	DD<br>FOX	*mecA*	NT
Alhashimi *et al* [99]	*S. aureus*	Asia	Iran/ Food handlers	Cross-sectional	332	99 (30.1)	C/PCR	99 (30.1) / NA	NA	NT	NT	*sea, seb, sec, sed, see*
Noor-Azira *et al* [100]	*S. aureus*	Asia	Malaysia/ Food handers	Cross-sectional	64	15 (23.4)	C	15 (23.4)/ 0.0	NT	DD<br>FOX	NT	NT
Wang *et al* [101]	*S. aureus*	Asia	China/ Pig-related farmers	Cross-sectional	335	43 (12.8)	C/PCR.MT	43 (12.8)/17 (5.1)	NT	DD<br>PEN, FOX, PEN, ERY, TET, CLI	*mecA*	*scn, chp, sak, sea*
Ho *et al* [102]	*S. aureus*	Asia	Hong Kong/ Raw and/or cooked foods handlers	Cross-sectional	434	99 (22.8)	C/PCR/ MT	99 (22.8) /5 (1.15)	NT	DD<br>FOX	*mecA*	*sea, seb*
Vatansever *et al* [103]	*S, aureus*	Asia	Turkey/ Food handlers	Cross-sectional	28	10 (35.7)	C/PCR	10 (35.7) / 0 (0.0)	NT	DD<br>FOX, TMP	NR	NT
Sepin-Özen *et al* [104]	*S. aureus*	Asia	Turkey/ Food-handlers	Retrospective	15600	526 (3.37)	C	526 (3.4) / NT	NT	NT	NT	NT
Šegalo *et al* [105]	*S, aureus*	Europe	Bosnia and Herzegovina/Food handlers	retrospective study in	1139	792 (7.1)	C	792 (7.1)/ NT	NA	NT	NT	NT
Uzunovic *et al* [106]	*S. aureus*	Europe	Bosnia and Herzegov	Cross-sectional	13690	189 (1.4)	C	189 (1.4) / 3 (0.02)	NT	NT	*mecA*	NT

			ina/ Food handlers									
Castro *et al* [13]	*S. aureus*	Europe	Portugal/ Food handlers	Cross-sectional	162	32 (19.8)	C/ PCR	32 (19.8) / 0 (0.0)	NA	DD<br>FOX	NA	*tst, sea, sej, seg, sei, sed, , seh, sec*$_{bov}$
De Jonge et al [107]	*S. aureus*	Europe	Netherlands/ Food handlers	Cross-sectional	95	31 (32.6)	C/PCR/MT	31 (32.6)/0 (0.0)	NA	DD<br>FOX	ND	ND
Sergelidis *et al* [108]	*S. aureus*	Europe	Greece/ Food handers (Salad)	Cross-sectional	12	6 (50.0)	C	6 (50.0) / (0.0)	NT	DD<br>OXA	NT	NT
Ribeiro and Clerigo, [109]	*S. aureus*	Europe	Portugal/ bakery worker	Cross-sectional	10	4 (40.0)	C	4 (40.0) /1 (25.0)	NT	NT	NT	NT
Cuny *et al* [110]	*S. aureus*	Europe	Germany / Butchers, meat sellers, and cooks	Cross-sectional	286	130 (45.5)	C/PCR/MT	130 (21.5)/ 2 (0.33)	NT	DD<br>FOX	*mecA*	NT
Leibler *et al* [111]	*S. aureus*	America	USA / Healthy Beef-packing workers	Cross-sectional	137	37 (27.0)	C / PCR	37 (27.0)/ 5 (3.6)	NA	DD<br>PEN, FOX, ERY, CLI, CIP	NT	NA
Acco *et al* [112]	*S. aureus*	America	Brazil/ Food handlers jelly fruit and cereal foods industry	Cross-sectional	47	14 (30)	C/PCR	14 (30.0) / NA	NT	DD<br>PEN, CIP, ERY, CLI	NT	NT
Rall *et al* [113]	CoPS and CoNS	America	Brazil/ Food handers	Cross-sectional	62	15 (22.1)	C/PCR	15 (22.1)/NR	S.ep, Sw	NT	NT	NT
Da Silva *et al* [114]	*CoPS, CoNS*	America	Brazil/ food handlers	Cross-sectional	60	12 (20.0)	C.PCR	12 (20.0)/0 (0.0)	Sw, Slug, Sep, Shae, Shom, Scoh, Ssim, Scap	DD<br>FOX,	ND	sea seb sec sed see seg seh sei
Aung *et al* [115]	*CoPS*	Asia	Burma/ food handlers	Cross-sectional	563	110 (19.5)	C/ PCR/MT	110 (19.5)/ 0 (0.0)	*Sarg, 1* (0.8%)	NT	NR	*tsst, lukS/F*-PV, *sea, sec*

[a] Detection method: C/MALDI/PCR/MT: Culture / MALDI-TOF-MS / PCR / Molecular typing
[b] SE*: S. epidermidis;* SHae*: S. haemolyticus;* SSa*: S. saprophyticus;* SL*: S. lugdunensis*; SCa: *S. capitis*; SC: *S. cohini:* SH: *S. hominis*; SHy: *S. hyicus;* SCs: *S. caseolyticus;* SSi: *S. simulans*; SD; *S. delphini;* SSli: S. *schleiferi* SW: *S. warnieri*; SPas: *S. pasteuri.; Sarg: S. argenteus*
cTechnique used: Disk-diffusion method (DD), MIC determination (MIC). Top 4 groups of antimicrobial agents: beta-lactams (BL). Fluoroquinolones (FQ), Macrolides (Mc), Tetracycline (Tet), Vancomycin (Van)
[d]NT: Not tested
NA: Not available (tested but result not published on article)
ND: Not detected
NR: Not reported in detail

PEN: Penicillin; AMX: Amoxicillin; ERY: Erythromycin; CHL: Chloramphenicol; CLI: Clindamycin; STR: Streptomycin; Van: vancomycin; TET: Tetracycline; OXA: Oxacillin; RIF: Rifampicin, FOX: Cefoxitin; LEV: Levofloxacin; CIP: Ciprofloxacin;; KAN: Kanamycin; GEN; Gentamycin; MUP: Mupirocin; SXT: Trimethoprim/sulfamethoxazole

Supplementary Table S3. Study characteristics, species, antimicrobial resistance, and virulence genes of staphylococcal nasal carriages in veterinary students and practitioners (n= 26)

Author (s) (reference)	Staphylococcal species	Continent	Country/Subjects	Study design	Sample size	No. of staphylococcal positive cases (%)	Detection method[a]	Detection of *S. aureus* (%)/ MRSA (%)	*Staphylococcus* species (non-*aureus*) detected (%)[b]	Antimicrobial resistance phenotype technique/ Major resistant types[c]	Antimicrobial Resistance genes	Virulence genes
Rasamiravaka *et al* [116]	*S. aureus*	Africa	Madagascar /Veterinary students	155	Cross-sectional	30 (19.4)	C	30 (19.4)/ 14 (9.03)	NT	DD<br>PEN, GEN, ERY, TET, VAN, OXA	NT	NT
Anueyiagu *et al* [23]	*S. aureus*	Africa	Nigeria/veterinary students	130	Cross-sectional	66 (50.8)	C	66 (50.8)/ 50 (38.4)	NT	DD<br>OXA, GEN, ERY, CHL, STR, CIP	NT	NT
Gaddafi *et al* [117]	*S. aureus*	Africa	Nigeria/Veterinarians	41	Prospective	NR	C/PCR	NR/ 10 (24.4)	NR	DD<br>PEN, OXA, ERY, NEO, GEN	*mecA*	NT
Kuroda *et al* [118]	*S. aureus*	Asia	Japan/Veterinarians	53	Prospective	NR	C/PCR	NR / 16 (30.1)	NT	DD method<br>FOX	*mecA*	NT
Zhang *et al* [119]	*S. aureus*	Asia	China/Veterinary Staff	51	Cross-sectional	12 (23.5)	C/PCR/MT	12 (23.5) /1 (1.9)		DD<br>PEN, OXA, CLI, ERY, TET, GEN	*nuc,mecA, linA, erm*(B), *aacA-aphD, tet*(K)	NT
Sekhar *et al* [120]	*S. aureus*	Asia	India/Veterinary Students	40	Prospective	15 (37.5)	C/PCR	15 (37.5)/ 4 (10.0)	10.0, 15.0 and 10.0 in owners pets, veterinary students respectively	DD<br>FOX, OXA	*mecA, blaZ*	*NT*
Kittl *et al* [121]	*S. aureus*	Europe	Switzerland /Veterinarians	212	Cross-sectional	NR	C/PCR/MT	NR/ 14 (6.6)	NT	NR	*mecA*, *aac*(6')-Ie-*aph*(2'')-Ia, *dfrK, tet*(M), *str, blaZ*	*chp, sak, scn, lukED, tst*
Huber *et al* [122]	*S. aureus*	Europe	Switzerland /Veterinarians	133	Cross-sectional	NR	C/PCR/MT	NR/ 4 (3.0)	NT	DD<br>PEN, OXA, ERY, CLI, GEN	*mecA*	ND
Wettstein-Rosenkranz *et al* [123]	*S. aureus*	Europe	Switzerland/Veterinary care providers	369	Cross-sectional	NR	C/PCR/MT	NR/ 14 (3.4)	NT	DD<br>PEN, FOX, GEN, TET	*mecA*,, *tet*(M) *tet*(K)], *dfrK*, *dfrG*, *aac*(6')-Ie – *aph*(2')- Ia, *blaZ*	*hlgA* hlgB hlgC sea, sed seg sei sej selm seln selo ser selu sak chp scn *lukED*

Wulf *et al* [124]	*S. aureus*	Europe	Denmark/ Veterinarians	272	Cross-sectional	NR	C/PCR/MT	NR/ 34 (12.5)	NT	DD FOX, ERY, CLI, TET, GEN, CIP, SXT	*mecA*	NT
Mroczkowska *et al* [125]	*S. aureus*	Europe	Poland /Veterinarians	38	Prospective	8 (21.1)	C/PCR/MT	8 (21.1)/ 4 (10.5)	NT	DD FOX	*mecA*	NR
Post *et al* [126]	*S. aureus*	Europe	UK/ veterinary Surgeon's conference	60	Cross-sectional	NR	C/PCR/MT	NR/3 (5.0)	NT	DD FOX, RIF, GEN, TOB, AMK, TET	*mecA*	*can, fib, sdrD*
Žemličková et al [127]	*S. aureus*	Europe	Czech/ Veterinarians	280	Cross-sectional	NR	C/PCR/MT	NR/ 2 (0.7)	NT	DD FOX	*mecA*	ND
Cuny et al [128]	*S. aureus*	Europe	Germany/ Pig Veterinarians	49	Cross-sectional	NR	C/PCR	NR/22 (44.9)	NT	DD PEN, OXA, TET, ERY, CLI	*mecA*	NT
Heller *et al* [129]	*S. aureus*	Europe	UK/ University clinic veterinarians	64	Cross-sectional	NR	C/PCR	NR / 2 (3.1)	NT	NR	*mecA*	NT
Moodley *et al* [130]	*S. aureus*	Europe	Denmark/Veterinary practitioners	222	Cross-sectional	NR	C/PCR	NR/ 9 (4.1)	NT	DD OXA, ERY, CLI, CHL, FD, MUP, SXT, TET	*mecA*	NT
Sun *et al* [131]	*S. aureus*	America	USA/ Swine veterinarians	66	Cross-sectional	43 (65.2)	C/PCR	43 (65.2)/6 (9.5)	NA	DD OXA	*mecA*	*scn, sak*
Anderson *et al* [132]	*S. aureus*	America	USA/ veterinary personnel	257	Cross-sectional	NR	C/ PBP2a immunoagglutination /PCR/MT	NR/26 (10.1)	NT	NT	*mecA*	NT
Hanselman *et al* [133]	*S. aureus*	America	Canada/Veterinary personnel	417	Cross-sectional	NR	C/PCR/PBP2a/MT	NR/27 (6.5)	NT	DD FOX, ERY, CIP, TET, GEN, SXT	*mecA*	ND
Worthing *et al* [134]	*S. aureus*	Australia	Australia/Veterinary personnel	46	Cross-sectional	NR	C/PCR/ MT	NR/4 (8.7)	NT	NT	*mecA*	NT
Neradova *et al* [135]	CoPS and CoNS	Europe	Czech Republic/Veterinary personnel	134	Cross-sectional	40 (29.9)	C/PCR/MT	40 (29.9)/ 9 (6.7)	SPs	DD FOX, CXT, TET, GEN, CIP	*mecA*	NT

Paterson *et al* [136].	CoPS and CoNS	Europe	UK/Cattle veterinary practitioners	307	Cross-sectional	NR	C/PCR/MT	NR/ 8 (2.6)	*SHae*	MIC<br><br>OXA, FOX, ERY, CLI, TET, SXT	*yrA tet*(M) *tet*(L), *aacA*, *aphD*, *dfrK erm*(C), *erm*(T), *mecA*	NT
Burstiner *et al* [137]	CoPS and CoNS	America	USA/Academic veterinary staff	341	Cross-sectional	NR	C/PCR/MT	NR/59 (17.3)	NT	DD<br><br>FOX	*mecA*	NT
Paul *et al* [138]	CoPS	Europe	Denmark/ small animal veterinarian	128	Prospective	NR	C/PCR	NR/ 2 (1.6)	MRSP, 5 (3.9)	DD<br><br>FOX	*mecA*	NT
Boost *et al* [139]	CoPS	Asia	Hong Kong/Veterinary personnel	150	Cross-sectional	NR	C/PCR/MT	NR/ 1 (0.7)	SPs	NT	*mecA*	NT
Malissiova *et al* [140]	CoNS	Europe	Greece/Healthy veterinary students	81	Prospective	22 (27.2)	C / PCR / MALDI	ND	SHae(54), SW(45.8), SE (16.6), SPas (4.2) SCa (4.2).	DD<br><br>FOX	*mecA (S. epidermidis)*	NT

[a] Detection method: C/MALDI/PCR/MT: Culture / MALDI-TOF-MS / PCR / Molecular typing; STE: *Staphylococcal* Enterotoxins

[b] SE*: S. epidermidis;* SHae*: S. haemolyticus;* SSa*: S. saprophyticus;* SL*: S. lugdunensis*; SCa: *S. capitis*; SC: *S. cohini:* SH: *S. hominis*; SHy: *S. hyicus;* SCs: *S. caseolyticus;* SSi: *S. simians*; SD; *S. delphini;* SSli: S. *schleiferi* SW: *S. warnieri*; SPas: *S. pasteuri.*

cTechnique used: Disk-diffusion method (DD-method), MIC determination (MIC). Top 4 groups of antimicrobial agents: beta-lactams (BL). Fluoroquinolones (FQ), Macrolides (Mc), Tetracycline (Tet), Vancomycin (Van)

[d]NT: Not tested

NA: Not available (tested but result not published on article)

ND: Not detected

NR: Not reported in detail

PEN: Penicillin; AMX: Amoxicillin; ERY: Erythromycin; CHL: Chloramphenicol; CLI: Clindamycin; STR: Streptomycin; Van: vancomycin; TET: Tetracycline; OXA: Oxacillin; RIF: Rifampicin, FOX: Cefoxitin; LEV: Levofloxacin; CIP: Ciprofloxacin;; KAN: Kanamycin; GEN; Gentamycin; MUP: Mupirocin; SXT: Trimethoprim/sulfamethoxazole

Supplementary Table S4. Study characteristics, species, antimicrobial resistance pattern, and virulence genes detected in staphylococcal nasal isolates recovered from healthy livestock farmers (n = 51).

Author (s) (reference)	Staphylococcal species	Continent	Country/Subjects	Sample size	Study design	No. of staphylococcal positive cases (%)	Detection method C/MALDI/PCR/MT[a]	Detection of *S. aureus* (%)/ MRSA (%)	*Staphylococcus species* (non-*aureus*) detected (%)[b]	Antimicrobial resistance phenotype technique[c]/ Major resistant types	Antimicrobial resistance genes	Virulence genes
Rasamiravaka *et al* [141]	*S. aureus*	Africa	Madagascar/ poultry non-industrial farmers	170	Cross-sectional	69 (38.3)	C/PCR	69 (38.3)/ 45 (26.4)	NT	DD<br><br>PEN, FOX, TET, OFX, COT, OXA, ERY	NT	NT
Mourabit *et al* [142]	*S. aureus*	Africa	Morocco/ livestock breeders	50	Cross-sectional	30 (60.0)	C/PCR	30 (60.0)/ 0 (0.0)	NT	DD<br><br>FOX, TET, ERY, KAN	ND	*tst-1*
Elemo *et al* [143]	*S. aureus*	Africa	Ethiopia/ dairy workers	98	Cross-sectional	38 (39.6)	C	38 (39.6)/ 23 (23.5)	NT	DD<br><br>PEN, AMP, FOX, OXA, TET, SXT	NT	NT
Adesida *et al* [144]	*S. aureus*	Africa	Nigeria/ farm personnel (cow, sheep, goats, pigs)	50	Prospective	28 (56.0)	C/PCR	28 (56.0)/ 3 (6.0)	NT	DD<br><br>OXA, FOX, SXT, ERY, GEN	*mecA*	NT
Okorie-Kanu *et al* [145]	*S. aureus*	Africa	Nigeria/ chicken and pig slaughterhouses workers.	45 each from chicken and pig slaughterhouses workers.	Comparative cross-sectional	5 (11.1) versus 3 (6.7)	C/PCR/MT	5 (11.1)/ 0 (0.0) versus 3 (6.7)/0 (0.0)	NT	DD<br><br>PEN, OXA, ERY, CLI, TET, SXT	ND	*lukS/F*-PV
Odetokun *et al* [146]	*S. aureus*	Africa	Nigeria/ Pig workers	260	Cross-sectional	35 (13.5)	C/PCR/MT	35 (13.5)/8 (3.1)		NT	*mecA*	*NT*
Otalu *et al* [147]	*S. aureus*	Africa	Nigeria/ pig farmers	Cross-sectional	55	C/PCR/MT	NR	NR/6 (10.1)	NT	DD<br>PEN, FOX	*mecA*	*scn*
Kalayu *et al* [148]	S. aureus	Africa	Ethiopia/ Diary farmers	Cross-sectional	71	C/PCR	22 (31.0)	22 (31.0)/ 1 (1.4)	NT	DD<br><br>FOX, CLI, ERY, PEN, TET, SXT	*mecA*	NT
Gaddafi *et al* [149]	*S. aureus*	Africa	Nigeria/ pig farm attendants	12	Cross-sectional	NR	C/PCR	NR/3 (25.0)	NT	DD<br><br>OXA, ERY, NEO, SUL, GEN	*mecA*	NT

Rongsanam *et al* [150]	*S. aureus*	Asia	Thailand/ swine production workers	202	Cross-sectional	NR	C/PCR	NR/16 (7.9)	NT	DD<br><br>PEN, FOX, TET, CIP, CLI, SXT, GEN	*mecA*	NT
Assafi *et al* [151]	*S. aureus*	Asia	Iraq/ broilers farm workers	75	Cross-sectional	63 (84.0)	C/PCR	63 (84.0)/18 (13.3)	*NT*	DD<br><br>OXA, VAN, CIP, CHL, AMK	*mecA*	NT
Moon *et al* [152]	*S. aureus*	Asia	Korea/ Pig farmers	126	Cross-sectional	NR	C/PCR/MT	NR/21 (16.7)	NT	DD<br><br>FOX	*mecA*	NT
Garipcin and Seker [153]	*S. aureus*	Asia	Turkey/ cattle farm workers	150	Cross-sectional	44 (29.3)	C/PCR	44 (29.3)/13 (8.7)	NT	DD<br><br>OXA, GEN, CIP, RIF, NEO, TET	*mecA*	NT
Cui *et al* [154]	*S. aureus*	Asia	China/ Pig workers	120	Cross-sectional	NR	C/PCR/MT	NR/ 2 (1.7)	NT	MIC<br><br>FOX, CIP, TET, GEN, CLI, ERY	*mecA*	NT
Fan *et al* [155]	*S. aureus*	Asia	China/ livestock workers	1162	Cross-sectional	108 (9.3)	C/PCR/MT	108 (9.3)/ 16 (1.4)	NT	DD<br><br>FOX, ERY, CLI, TET, RIF, CHL, CIP, LZD	*mecA*	*chp*, *scn*, *sea*, *sak*, *sep*
Ye *et al* [156]	*S. aureus*	Asia	China/ Pig workers	244	Cross-sectional	37(15.2)	C/PCR/MT	37(15.2)/ 26 (10.7)	NT	DD<br><br>FOX, CLI, TET, ERY, CHL, CIP	*mecA*	*scn*, *sea*, *sak*, *chp*
Fang *et al* [157]	*S. aureus*	Asia	Taiwan/ pig farm workers	100	Cross-sectional	NR	C/PCR/MT	NR/13 (13.0)	NT	DD<br><br>PEN, DOX, FOX, CIP, ERY, CLI, SXT	*mecA*	*lukS/F*-PV
Lim et *al* [158]	*S. aureus*	Asia	Korea/ Diary farm workers	43	Cross-sectional	NR	C.PCR	NR/1 (2.3)	NT	DD<br>FOX	*mecA*	NT
Back *et al* [159]	*S. aureus*	Asia	Korea/ pig farm workers	186	Prospective	NR	C/PCR/MT	NR/ 6 (3.2)	NT	DD<br><br>AMP, FOX, CHL, CLI, ERY, CEF, GEN, TET, CIP	*mecA*, *tet*(M), *tet*(K), *tet*(L)	*sed*
Jayaweera and Kumbukgolla [160]	*S. aureus*	Asia	Sri Lanka/ livestock farmers (Pigs, cattle, poultry)	94 (31 in piggery, 32 in poultry and 31 in diary)	Cross-sectional	NR	C/PCR	NR/ 15 (15.9)<br><br>8 in Pig,	NT	DD<br><br>OXA, ERY, CLI, TET, GEN	*mecA*	NT

								4 in Poultry, 3 in cattle farmers				
Normanno *et al* [161]	*S. aureus*	Europe	Italy/ pigs and abattoir workers	113	Cross-sectional	NR	C/PCR/MT	NR/ 9 (7.9)	NT	DD<br><br>FOX, CLI, ERY, KAN, TET	*mecA*	ND
Aubry-Damon *et al* [162]	*S. aureus*	Europe	France / African pig farmers	112	Cross-sectional	50 (44.6)	C	50 (44.6)/ 5 (4.5)	NT	DD<br><br>OXA, PEF, GEN	NT	NT
Etter *et al* [163]	*S. aureus*	Europe	Switzerland / famers	160 (87 pupils and 73 members of farmer families)	Cross-sectional	73 (45.6)	C/MALDI/PCR/MT	73 (45.6)/2 (1.25)	NT	NR	*mph*(C), *aphA3, fosB, mecA, blaZ, InuA, aadD, tet*(K), *fosB*	*tst, sea, sab, sec, sel, sec, seg, sel, egc*
Kittl *et al* [121]	*S. aureus*	Europe	Switzerland / animal farmers (pigs)	156	Cross-sectional	NR	C/PCR/MT	NR / 8 (5.1)	NT	NT	*mecA, dfrG, erm*(A), *erm*(C), *spc, tet*(K), *tet*(M), *vga*(E), *blaZ*	*scn, chp, sak, tsst*
VAN CLEEF *et al* [164]	*S. aureus*	Europe	Netherlands/ pig slaughterhouse workers	Cross-sectional	249	C/PCR	NR	NR/14 (5.6)	NT	DD<br><br>TET, ERY, CLI, SXT, GEN, TOB	*mecA*	NT
Cuny et al [128]	*S. aureus*	Europe	Germany/ Pig farmers	113	Cross-sectional	NR	C/PCR	NR/97 (85.8)	NT	DD<br>PEN, OXA, TET, ERY, CLI	*mecA*	NT
Van cleef *et al* [165]	*S. aureus*	Europe	Netherlands/ pig farmers	Cross-sectional	120	C/PCR	NR	NR/ 53 (44.2)		DD<br>FOX	*mecA*	NT
Papadopoulos *et al* [166]	*S. aureus*	Europe	Greece/ Diary workers	Cross-sectional	26	C/PCR/MT	10 (38.5)	10 (38.5) /2 (7.7)	NT	DD<br><br>OXA, PEN, GEN, KAN, VAN	mecA	Sea, sec
Parisi *et al* [167]	*S. aureus*	Europe	Italy/ Pig farm workers	Cross-sectional	150	C/PCR/MT	NR	NR/26 (17.3)	NT	DD<br><br>AK, AMP, CTX, CHL, ERY, SXT, TET, FOX	*mecA*	NT
Sakwinska *et al* [168]	*S. aureus*	Europe	Switzerland/ livestock workers	Cross-sectional	158	C/PCR/MT	57 (36)	57 (36)/ 2 (1.3)	NT	NT	*mecA*	NT
Antoci *et al* [169]	*S. aureus*	Europe	Italy/ Diary farmers	Cross-sectional	113	C/PCR/MT	NR	NR/ 40 (36.0)	NT	DD<br><br>OXA, FOX	*mecA*	ND

van Cleef *et al* [170]	*S. aureus*	Europe	Denmark/ pig farm workers	16	Cross-sectional	16 (100.0)	C/PCR/MT	16 (100.0)/2 (12.5)	NT	NR	*mecA*	NT
Fischer *et al* [171]	*S. aureus*	Europe	Germany/ pig farmers	85	Cross-sectional	NR	C/PCR/MT	NR/ 72 (84.7)	NT	DD<br>FOX	NT	NT
Bunke *et al* [172]	*S. aureus*	Europe	Germany/ Pig farmers	86	Cross-sectional	54 (62.8)	C/PCR	54 (62.8)/ 18 (20.9)	NT	DD<br>FOX	*mecA*	NT
Schnitt *et al* [173]	*S. aureus*	Europe	Germany/Cattle worker	14	Prospective	NT	C/ PCR/MT	NR/6 (42.9)	NT	DD<br>FOX	*mecA*	NT
Dahms *et al* [174]	*S. aureus*	Europe	Germany/ pig farmers	78	Cross-sectional	NR	C/PCR/MT	NR/ 20 (25.6)	NT	DD<br>OXA, TET, ERY CLI, CIP	*mecA*	ND
Kock *et al* [175]	*S. aureus*	Europe	Germany/ pig farmers	35	Prospective	NR	C/PCR/MT	NR/27 (77.1)	NT	DD<br>FOX	*mecA*, *blaZ*, *tet*(M), *tet*(K), *erm*(A), *erm*(B), *erm*(C)	NT
Bisdorff et al [176]	*S. aureus*	Europe	Germany/ Livestock workers	190	Cross-sectional	NR	C/PCR/MT	NR/46 (24.2)	NT	DD<br>FOX	NT	NT
Giovanni *et al* [177]	*S. aureus*	Europe	Italy/ buffalo milk tank operators	24	Cross-sectional	NR	C/PCR/MT	NR/1 (4.00)	NT	MIC<br>OXA, FOX	*mecA*	*icaA*
Pirolo *et al* [178]	*S. aureus*	Europe	Italy/ pig farmers	88	Cross-sectional	49 (55.7)	C/PCR/MT	49 (55.7)/ 19 (21.6)	NT	DD<br>PEN, FOX, SXT, GEN, TET, ERY	*mecA*	NT
Mascaro *et al* [179]	*S. aureus*	Europe	Italy/ sheep farmers	275	Cross-sectional	97 (35.5)	C/ PCR/MT	97 (35.5)/3 (1.1)	NT	DD<br>OXA, FOX, TET, ERY, CLI, LZD	*mecA*	NT
Graveland *et al* [180]	*S. aureus*	Europe	Netherlands/ veal Calf Farmers	97	Cross-sectional	NR	C/PCR/MT	NR/32 (33.0)	NT	NT	*mecA*	NT
Mroczkowska *et al* [125]	*S. aureus*	Europe	Poland /pig farmers	283	Cross-sectional	50 (17.7)	C/PCR/MT	50 (17.7)/ 9 (3.2)	NT	DD<br>FOX	*mecA*	NR
Reynaga *et al* [181]	*S. aureus*	Europe	Spain/ pig farm workers	140	Cross-sectional	NR	C/ PBP2a Immunoagglutination/ PCR/MT	NR/ 81 (57.9)	NT	DD	*mecA*	NT

										OXA, FOX, TET, ERY, CLI, CIP, GEN, TOB, SXT		
Neyra *et al* [182]	*S. aureus*	America	USA/ hog plant workers	162	Cross-sectional	35 (21.6)	C/PCR/MT	35 (21.6) 9 (5.6)	NT	DD<br>FOX, ERY, CIP, CLI, TET	*mecA*	NT
Hatcher *et al* [183]	*S. aureus*	America	USA/ hog Operation Workers	198	Cross-sectional	105 (53.0)	C/PCR/MT	105 (53.0)/32 (16.6)	NT	DD<br>PEN, OXA, ERY, TET, AMP, CLI	*mecA*	*scn*
Rinsky *et al* [184]	*S. aureus*	America	USA/ industrial livestock operation and antibiotic-free livestock operation workers (pigs)	99 versus 105	Comparative cross-sectional	41 (41.0) versus 42 (40.0)	C/PCR/MT	41 (41.0)/ 3 (3.0) versus 42 (40.0)/ 3 (2.9)	NT	DD<br>OXA, GEN, TET, ERY	*mecA*	*scn*
Smith *et al* [185]	*S. aureus*	America	USA/ pig farm workers	148	Cross-sectional	NR	C/PBP2a Immunoagglutination PCR/MT	NR/31 (20.9)	NT	DD<br>FOX	NT	NT
Khanna *et al* [186]	*S. aureus*	America	Canada/ Pig farmers	25	Prospective	NR	C/PBP2a Immunoagglutination/ /MT	NR / 5 (20.0)	NT	NT	NT	NT
Sahibzada *et al* [187]	*S. aureus*	Australia	Australia/ Pig farmers	52		NR	C/PCR	NR/ 31 (59.6)	NT	NT	*mecA*	NT
Sinlapasorn *et al* [188]	CoPS and CoNS	Asia	Thailand/ Pig farmers	98	Cross-sectional	3 (3.1)	C/PCR/MT	3 (3.1) /3 (3.1)	*SSci, Shae, Sart, S. cohnii subspecies urealyticus, S. hominis subspecies hominis, SChr, SE*	MIC<br>OXA, FOX, GEN, ERY, TET	*mecA*	NT
Founou *et al* [189]	CoNS	Africa	Cameroon and South Africa/ Pig workers	53 in Cameroon and 24 in South Africa	Comparative prospective	ND	C/PCR	ND/ND	50 (94,3) and 7 (29.2) MRS in Cameroon and South Africa	DD<br>PEN, OXA, FOX, TET, SXT	*mecA*	NT

									receptivel y.<br>*SE, Shae, SL, SHom*			
Argudın *et al* [190]	CoNS	Europe	Belgium/ farmers	86	Cross-sectional	NR	C/PCR/MT	NR	Sep, 64 (75)<br>MRSE, 44 (51.1)	DD<br>PEN, FOX, CLI, KAN, TET, ERY, SXT	*mecA*	NT
Roberts *et al* [191]	CoPS and CoNS	America	USA/ healthy dairy farmers	24	Cross-sectional	5 (20.8)	C/PCR	5 (20.8)/NR	Sep, Shae, Shom, Ssci, Sxy	NT	NT	NT

[a] Detection method: C/MALDI/PCR/MT: Culture / MALDI-TOF-MS / PCR / Molecular typing
[b] SE*: S. epidermidis;* SHae*: S. haemolyticus;* SSa*: S. saprophyticus;* SL*: S. lugdunensis*; SCa: *S. capitis*; SC: *S. cohini:* SH: *S. hominis*; SHy: *S. hyicus;* SSi: *S. simulans*; SD; *S. delphini;* SSli: S. *schleiferi* SW: *S. warnieri*; SPas: *S. pasteuri: Schr: S. chromogenes; Sart: S. arlettae*
[c]Technique used: Disk-diffusion method (DD), MIC determination (MIC). Top 4 groups of antimicrobial agents: beta-lactams (BL). Fluoroquinolones (FQ), Macrolides (Mc), Tetracycline (Tet), Vancomycin (Van)
[d]NT: Not tested
NA: Not available (tested but result not published on article)
ND: Not detected
NR: Not reported in detail

PEN: Penicillin; AMX: Amoxicillin; ERY: Erythromycin; CHL: Chloramphenicol; CLI: Clindamycin; STR: Streptomycin; Van: vancomycin; TET: Tetracycline; OXA: Oxacillin; RIF: Rifampicin, FOX: Cefoxitin; LEV: Levofloxacin; CIP: Ciprofloxacin;; KAN: Kanamycin; GEN; Gentamycin; AMK: Amikacin; NEO: Neomycin; MUP: Mupirocin; SXT: Trimethoprim/sulfamethoxazole; LZD: Linezolid

Supplementary Table S5. Prevalence pattern and genetic profile of PVL-positive *S. aureus* isolates in the nasal cavity of healthy people of the Groups A-D.

Author (s) (Reference)	Country/ Subjects	Sample size/ No. S. aureus/ No. MRSA	Frequency and Prevalence (%) of PVL in all *S. aureus*	Frequency and Prevalence (%) of PVL in MSSA	Frequency and Prevalence of PVL (%) in MRSA	Genetic lineages (*spa*, CC or ST) (n) in PVL-MRSA	Genetic lineages (*spa*, CC or ST) (n) in PVL MSSA
Eibach *et al* [33]	Ghana/Healthy children	544/ 120/ (22.1)/ 2 (0.37)	69 (57.5)	69 (58.5)	0 (0.0)	ND	a. CC152/ t1096 (3), t355 (25), t1123 (2), t1172 (1), t1299 (4), t5047 (1)<br>b. CC1/ t127 (3), t591 (1)<br>c. CC121/ t311 (9), t314 (7), t645 (1), t1114 (2)<br>d. CC15/ t84 (18), t085 (1), t346 (1), t385 (1)<br>e. CC30/ t318 (1), t363 (3), t2147 (1)<br>f. CC8/ t451 (1)<br>g. CC88/ t186 (1), t4104 (2)
Egyi *et al* [34]	Ghana/ Healthy people	234/ 49 (21.0)/ 2 (0.3)	34 (69.4)	34 (72.3)	0 (0.0)	ND	a. CC5/ t10839(1), t311(3), t071(1)<br>b. CC15/ t7568(1), t084(9), t346(1), t10843(1), t10845(1)<br>c. CC30/ t021(2), t363(1)<br>d. CC97/ t359(1)<br>e. CC45/ t2771(3), t5602(3), t6038(2), t1996(1) t065(1), t10834(1), t10840(1), t10841(1), t1510(1) t3986(1), t861(2), t939(1), t2784(1) t8453(1)<br>f. CC121/ t091(2), t4499(1), t645(1)<br>g. CC152/ t454 (1), t355(12)<br>h. CC707/ t1458(1)
Gong *et al* [48]	China/ Healthy children	314/ 16 (5.10) / 3 (0.95)	10 (62.5)	10 (76.9)	0 (0.0)	NT	NT
Mourabit *et al* [36]	Morocco / healthy volunteers	465/ 178 (38.3)/ 21 (4.5)	22 (12.3)	22 (14.1)	0 (0.0)	ND	NR
Yan *et al* [49]	China/Healthy adults	2448/ 403 (16.3)/8 (0.33)	9 (2.2)	9 (2.3)	0 (0.0)	ND	a. CC5/t002 (2)<br>b. CC398 / t011 (1)<br>c. CC7/ t091 (1)<br>d. CC88/ t1376 (1)<br>e. CC6/ t701 (1)<br>f. CC5/ t167 (1)<br>g. CC121/ t645 (1)<br>h. CC22/ t7611 (1)

Karapsias *et al* [59]	Greece/ Healthy Air force staff	959/ NR/ 9 (0.94)	NR	NR	1 (11.1)	ST80/t044 (1)	NR
Kirkliauskienė *et al* [60]	Denmark/Healthy persons	537/ 273 (50.8)/ 0 (0.00)	8 (2.9)	8 (2.9)	ND	ND	NT
Gorwitz *et al* [71]	USA/Healthy people	9004/ 2575 (28.6) /135 (1.5)	NR	NR	20 (14.8)	NT	NT
Velasco *et al* [69]	USA/ Healthy people	550/ 42 (7.6)/ 0 (0.0)	0 (0.0)	0 (0.0)	0 (0.0)	ND	ND
Wardyn *et al* [73]	USA/Healthy rural community	131/ 31 (23.7)/ 0 (0.0)	9 (29.0)	9 (29.0)	0 (0.0)	ND	t021 (3), t2104 (2), t4106 (1), t4032 (1), t015 (1), t3274 (1), t279 (2)
Wardyn *et al* [72]	USA/Healthy community members	1342/ 156 (22.5)/ 19 (2.7)	5 (3.2)	NR	NR	NR	NR
Hanselman *et al* [78]	Canada/Healthy school teachers	220/ 79 /7 (3.2)	1 (14.2)	ND	1 (14.2)	NT	NT
Gardella *et al* [79]	Argentina/Healthy children	316/98 (31.0)/ 14 (4.4)	NR	0 (0.0)	4 (28.6)	ST5/t311 (3), t2121 (1)	NR
Aung *et al* [115]	Burma/ food handlers	563 / 110 (19.5)/ 0 (0.0)	15 (13.6)	15 (13.6)	0 (0.0)	ND	CC96 (10), CC1 (2), CC8 (1), CC88 (1), CC2250 (1)
Okorie-Kanu *et al* [145]	Nigeria/ chicken and pig slaughterhouses workers.	45 each/ 5 (11.1)/ 0 (0.0) versus 3 (6.7)/0 (0.0)	3 (37.5)	3 (37.5)	0 (0.0)	ND	CC5/ t311 (1), t279 (1)<br><br>CC-ND/t18346 (1)
Fang *et al* [157]	Taiwan/ pig farm workers	100/ NR/13 (13.0)	NR	NR	1 (7.7)	ST59/t3527	NR
Becker *et al* [63]	Germany/ Healthy population	1878/920 (40.9)/131 (0.7)	2 (0.22)	2 (0.25)	0 (0.0)	ND	t216 and t004
Netsvyetayeva *et al* [64]	Ukraine/ heakthy people	245/ 99 (40.4)/ 9 (3.7)	58 (58.6)	53 (58.9)	5 (55.6)	NT	NT

NT, not tested.
NR, tested but not reported.
ST, Sequence Type.
CC, Clonal Complex
ND, Not detected (after testing)

Supplementary Table S6. Molecular typing of *S. aureus* isolated from the nasal cavity of healthy humans with or without occupational risks of colonization (Groups A-D).

NT: not tested.
NR: tested but not reported.
ST: Sequence Type.
CC: Clonal Complex
ND: Not detected (after testing)

Author (s) (reference)	Country/Subjects	No. of samples /No. of *S. aureus* positive cases (%)/ No. of MRSA (%)	*spa* types/ predominant, n (%) in MSSA isolates	ST pattern/ predominant, n (%) MSSA all isolates	CC pattern/ predominant, n (%) in MSSA isolates	*spa* types/ predominant, n (%) in MRSA	ST pattern/ predominant, n (%) in MRSA	CC pattern/ predominant, n (%) in MRSA
**Healthy population without reported occupational risk of nasal staphylococci colonization (Group A)**								
Lozano *et al* [67]	Spain/ Healthy persons	278/ 53 (19.1)/ 1 (0.36) MRSA	t002, t008, t012, t018, t021, t084, t091, t122, t127, t159, t190, t207, t209, t216, t228, t233, t270, t342, t331, t375, t548, t571, 1 (1.9), 706, t985, t1008, t1318, t1451, 1 (1.9), t1641, t2147, t2949, t5955, t6092, t6287, t6288, t6289, t6647 / t012, 6 (11.3) and t002, 5 (9.4)	ST30, ST34, ST1731, ST509, ST1498, ST59, ST1141, ST121, ST398, 2 (6.7), ST97, ST12, ST121, ST1, ST5 / ST30, 9 (16.9)	CC30, CC509, CC8, CC59, CC109, CC121, CC398, 2 (3.8), CC97, CC12, CC1, CC5, CC15, CC7, CC45/ CC30, 17 (32.1)	t7701, 1	ST1649, 1	CC6, 1
Yan *et al* [49]	China/Healthy adults	2448/ 403 (16.3)/ 8 (0.3)	77 *spa* types detected/ t571, 44 (10.9) and t189, 40 (9.9)	ST398, ST2139 ST6, ST2114, ST7 ST45, ST59, ST2148, ST211, ST25, ST123, ST22/ ST398, 44 (10.9)	CC398 CC1, C7, CC45, CC59, CC88, CC6, CC22, CC121, CC5/ CC398, 44 (10.9)	t034, t2431, t437, t116 / t034, 2 (25/0)	ST398, ST2799, ST59, ST45 / ST398, 2 (25.0%)	CC398, CC59, CC45 / CC398, 2 (25.0)
El-Mahdy *et al* [39]	Saudi Arabia/Healthy adults	70/26 (37.1)/ 1 (1.4) MRSA	t084, t131, t1328, t274, t065, t688, t002, t024, t267, t128, t5078, t5078, t937, t304, t349, t355, t159/ t084, 8 (30.7)	ST80, ST2196, ST291, ST789, ST152, ST2816/ ST80, 3 (11.5)	CC15, CC80, CC22, CC30, CC45, CC5, CC8, CC97, CC1, CC101, CC22, CC121/ CC15, 8 (30.7)	t128 (1)	NT	CC1 (1)
Muthukrishnan *et al* [68]	USA /Healthy persons	109/61 (55.9) / 7 (11.5)	t334, t008, t037, t012, t1705, t189, t954, t002, t216, t3682, t209 / t008, 10 (24.4) in both intermittent and persistent carriers	ST5, ST8, ST15, ST18, ST30, ST45, ST59 / ST5, 12 (29.3) in both intermittent and persistent carriers	NR	NR	ST5, ST8, ST15, ST30, ST87/ ST30, 3 (42.9)	NR
Ritchie *et al* [57]	Germany/Healthy students	122/ 69 (57.0)/ NT	Data of *S. aureus* 43 *spa* types of 69 isolates: t002, t019, t091, t127, t189, t224, t732, t3182, t1497, t709, t359, t065, t363, t092, t4224, t338, t148, t548, t1084, t359 and others/ t189 (12 episodes), t002 (9), t127 /) and t019, t091 and t224 (3).	NT	NT	NT	NT	NT

Ebruke *et al* [38]	Gambia/ Healthy villagers	600/ 151 (25.2)/ 0 (0.0)	NR	ST1, ST5, ST6, ST8, ST15, ST25, ST30, ST41, ST45, ST72, ST97, ST121, ST199, ST508, ST509, ST567, ST669, ST707, ST728, ST730, ST1004, ST1320, ST1472, ST1973 to ST2006/ ST15 (28 %) and ST5 (4 %) of 100 isolates	NT	ND	ND	ND
Velasco *et al* [69]	USA/ Healthy people	550/ 42 (7.6)/ 0 (0.0)	NT	ST5, ST15, ST30, ST34, ST39 and ST45	ND	ND	ND	ND
Wardyn *et al* [72]	USA/ Healthy community members	693/ 156 (22.5), 19 (2.7) MRSA	Among *S. aureus* (>90% MSSA) 118 *spa* types identified: t002, t034, t012, t338, t216, t571 and others/ t002, 33 (9.4%), t034 29 (8.3%)	ST398 and ST9/ ST398, 69 (50.4)	NR[a]	NR[a]	NR[a]	NR[a]
Gardella *et al* [79]	Argentina/ Healthy children	316/ 98 (31.0)/ 14 (4.4)	NT	NT	NT	t002, t012, t127, t311, t2121 t2365 / t002, 5 (34.5), t313 (23%)	ST5 in 3 (21.4) MRSA	NT
Eibach *et al* [33]	Ghana/ Healthy children	544/120 (22.1)/ 2 (0.34)	t1096, t008 (3), t1123, t318, t363, t2147, t1172, t1299, t5047, t355, t84, t939, t157, t4454 , t939/ t355, 25 (20.3)	ST121, ST15, ST30, ST152, ST45, ST707, ST508, ST577, ST8, ST88/ ST 152, 36 (21.6)	CC121, CC15, CC30, CC152, CC45, CC707, CC508, CC8, CC88/ CC152, 36 (21.6), CC15, 21(17.8), CC45, 19 (15.2)	t1096, t4454	ST45, ST152	CC152, CC45
Ngoa *et al* [35]	Gabon/ Healthy community	205/ 60 (29.3)/ 1 (0.49)	t148 (5%), t279 (4%), t1510 (4%), t127 (4%), t1045 (4%), t002 (4%), t355 (3%) and t2723 (2%)/ t084, 23 (38.3)	ST5, ST9, ST6, ST8, ST27, ST1, ST15, ST88, ST25, ST30, ST345, ST508/ ST15, 23 (38.3)	CC5, CC 6, CC 9, CC8, CC15, CC25, CC30, CC101, CC121, CC152/ CC84 23 (38.3)	t729	ST88	CC88
Mehraj *et al* [61]	Germany / healthy people	389/85 (21.9)/5 (1.3)	t084, t091, t254, t360, t368, t491, t499, t4508, t015, t073, t550, t772, t2277, t13383, t005, t032, t1433, t1862, t012, t018, t019, t021, t275, t330, t706, t2275, t6311, t002, t242, t1265, t304, t2032, t11363, t056, t7760, t13588, t159, t160, t537, t1057, t1541, t2313, t3741, t5337, t7088, t10983, t13449, t13587/ t012 , 16 (20.0)		CC15, CC30, CC45, CC22, CC5, CC8, CC101/ CC30, 16 (20.0)	t025, t032/ t025, 4 (80.0)	NT	CC 22, 5 (100.0)
den Heijer *et al* [62]	Austria/ health people	3309/549 (16.6)/ 8 (0.2)	NT	NT	NT	t003, t005, t008, t010, t015, t034, 1 (12.5), t127	NT	NR
den Heijer *et al* [62]	Belgium/ healthy people	3025/584 (19.3)/12 (0.4)	NT	NT	NT	t008, t011, 2 (16.7), t038, t062, t231, t447 (n=3), t1923, t2346, t10847	NT	NR

den Heijer *et al* [62]	Croatia/ healthy people	3960/792 (20.0)/15 (0.4)	NT	NT	NT	t002, t003, t005, t011, 1(6.7), t014, t015, t045, t050, t127, t330, t535, t550, t2018, t5933, t10807	NT	NR
den Heijer *et al* [62]	France/ healthy people	3858/876 (22.7)/16 (0.4)	NT	NT	NT	t002, t008, t010, t121, t622, t681, t777, t2054, t5708	NT	NR
den Heijer *et al* [62]	Hungary/ healthy people	3847/542 (14.1)/8 (0.2)	NT	NT	NT	t002, t032, t127, t330, t1218	NT	NR
den Heijer *et al* [62]	Netherlands/ healthy people	3847/1073 (27.9)/9 (0.2)	NT	NT	NT	t011, 2 (22.2), t034, 1 (11.1), t038, t108, 1 (11.1), t267, t740, t1457, t10812	NT	NR
den Heijer *et al* [62]	Spain/ healthy people	3990/770 (19.3)/10 (0.3)	NT	NT	NT	t002 (n=2), t022, t230, t846, t1081, t1203, t1610, t10814	NT	NR
den Heijer *et al* [62]	Sweden/ healthy people	3214/958 (29.8)/ 0 (0.0)	NT	NT	NT	ND	ND	ND
den Heijer *et al* [62]	UK/ healthy people	3156/814 (25.8)/13 (0.4)	NT	NT	NT	t002, t020, t025, t032, t127 (n=2), t223, t852, t1214, t2436, t5414, t7922	NT	
Becker *et al* [63]	Germany/ healthy population	1878/920 (40.9)/15 (0.8)	331 spa types with t084 (n = 104; 7.7%), t091 (n = 83; 6.1%), t012 (n = 71; 5.2%) and t015 (n = 56; 4.1%) being predominant. In addition, t011 (n = 3), t034 (n = 5), t1451 (n = 4) and or t3423 (n = 1) were identified.	NT	NR	t003, t011, t084, t127, t230, t469 , t670, t159, t009,/ t011, 4 (26.7)	NT	NR
Pires *et al*. [74]	Brazil/ Healthy community	686/224 (32.7)/ 6 (0.9)	NT	NR	NR	t002 and t021/ t002, 5 (83.3)	ST5, ST1176, ST2594/ ST5, 4 (66.7)	NT
Karapsias *et al* [59]	Greece/ Healthy Air force staff	959/ 9 (0.94) MRSA	NT	NT	NT	t012 t018, t044, t046, t1051/ t012, 3 (33.3)	NT	NT
**Healthy Food Handlers (Group B)**								
Cuny *et al* [110]	Germany/ Butchers, meat sellers, and cooks	286/ 130 (21.5)/ 2 (0.33)	52 *spa*-types detected: t084, t091, t005, t008, t002, t127, t012, t015, t073, t346, t571 (2), t1451 (2) / t084, 35 (26.9) and t091, 35 (26.9)	ST182	CC1, CC5, CC7, CC8, CC9, CC15, CC22, CC30, CC34, CC101, CC121, CC398, 4 (3.1)/ CC22, 21 (16.2)	t032, 2 (100.0)	ST21, 2 (100.0)	CC22, 2 (100.0)
Ho *et al* [102]	Hong Kong/ Raw and/or cooked foods handlers	434/ 99 (22.8)/ 5 (1.15)	42 *spa* types detected: t188, t127, t034, 3 (3.2), t3625, t091, t1081, t338, t304, t843 and others/ t188, 17 (17.2%)	ST15, ST7, ST251, ST239, ST45, ST30, ST5, ST8, ST6, ST1, ST398, 4 (4.2), ST96, ST188, ST37, ST9, ST59, ST130/ ST188, 17 (18.1)	NT	t1081, t4189/ t1081, 4 (80.0)	ST45, 3 (60.0)	NT

Uzunovic *et al* [106]	Bosnia and Herzegovina/ Food handlers	13690/ 189 (1.4)/ 3 (0.02)	66 spa-types reported. Including t005, t091, t015 and t008, t159, t012, t021, t267 and t010/ t005, 20 (10.7)	ST45, ST22, ST121, ST5, ST8, ST15, ST30, ST7, ST1, ST1, ST3/ ST45, 41 (22.0)	CC015, CC192, CC159, CC02, CC084, CC008, CC024, CC021, CC012/ CC015, 41 (22.0)	t355 (2), t595 (1)	ST152 (3)	CC355/CC595 (3)
Leibler *et al* [111]	USA / Healthy Beef-packing workers	137/ 37 (27.0)/ 5 (3.6)	t021, t065, t078, t189, t276, t304, t458, t571, 1 (3.1), t688, t701, t992, t1250, t3182, t4298, t4976, t6150, t002, t008, t024, t338, t748, t1248, t1476, t2379/ t1248 , 4 (12.5)	NT	NT	t002, t008 and t024/ t008, 2 (40.0) and t024, 2 (40.0)	NT	NT
Aung *et al* [115]	Burma/ food handlers	563 / 110 (19.5)/ 0 (0.0)	NT	ST88, ST683, ST45, ST1930, ST3206, ST1, ST672, ST6, ST2641, ST2885, ST59, ST15, ST8, ST5, ST779, ST508, ST3371, ST3206/ ST1930, 12 (38.7) out of 31 selected isolates	CC88, CC8, CC45, CC96, CC1, CC672, CC6, CC59, CC15, CC5, CC779 / CC96, 11 (35.4) out of 31 selected isolates	ND	ND	ND
Boost *et al* [98]	Hong Kong/ Pork Butchers	300/ 17 (5.6) MRSA	NR	NR	NR	t701, t008, t002, t123, t359/ t701, 5 (29.4)	NT	CC6, CC8 CC5, CC97, CC45/ CC6, 5 (29.4)
**Healthy Veterinarians (Group C)**								
Boost *et al* [139]	Hong Kong/ veterinary personnel	150 / 1 (0.7) MRSA	NT	NT	NT	t701	NT	CC6
Zhang *et al* [119]	China/ Veterinary personnel	51/ 1 (1.9) MRSA	NT	NT	NT	t437	ST59	NT
Kittl *et al* [121]	Switzerland / veterinarians	212/ 14 (6.6) MRSA	NT	NT	NT	t011, t118, t17424, t038, t133, t034, t899, t5634, t1510/ t011, 8 (57.1)	ST398, ST225, ST8, ST22, ST45 / ST398, 8 (57.1)	NT
Huber *et al* [122]	Switzerland / veterinarians	133/ 4 (3.0) MRSA	NT	NT	NT	t127, t011, t064/ t011, 2 (50.0)	ST398, ST8/ ST398, 3 (75.0)	NT
Wulf *et al* [124]	Denmark/ Veterinarians	272/ 34 (12.5) MRSA	NT	NT	NT	t011, t034, t108, t571, t567, t899/ t011, 15 (44.1)	ST398, 34 (100.0)	CC398, 34 (100.0)
Neradova *et al* [135]	Czech Republic/ veterinary personnel	134 / 9 (6.7) MRSA	NT	NT	NT	t011, t034, t003, t2330 / t011, 6 (66.7)	ST398, ST225, ST4894/ ST398, 7 (77.9)	CC398 in 8 (88.9%) (CC11-*spa* type)
Žemličková et al [127]	Czech Republic/ veterinary personnel	280/2 (0.7) MRSA	NT	NT	NT	t012, t026	ST30, ST45	NT
Wettstein-Rosenkranz *et al* [123]	Switzerland/ veterinary care providers	369/14 (3.4) MRSA	NT	NT	NT	t011, t003, t014, t002, t283, t186/ t011, 4 (28.6)	ST398, ST225, ST5, ST88/ ST398, 4 (28.6)	NT
Paterson *et al* [136]	UK/ cattle veterinary practitioners	307/ 8 (2.6) MRSA	NT	NT	NT	t032, t011, t008, t216, t879/ t032, 3 (37.8)	ST22, ST398, 1 (12.5) ST2274, ST2014, ST8, ST59 / ST22, 3 (37.8)	CC22, CC398, CC2014, CC8, CC59 / CC22, 4 (50.0)

Anderson *et al* [132]	USA/ veterinary personnel	257 / 26 (10.1) MRSA	NT	NT	NT	t002, t067, t985, t064, t451, t622 / t064, 9 (34.6) and t002 (23.1)	NT	NT
Worthing *et al* [134]	Australia/ healthy veterinary personnel	46 / 4(8.7) MRSA	NR	NR	NR	t316, t441, t177, t976	ST59, ST336, ST81/ ST59, 2(50.0)	NT
**Healthy Livestock Farmers (Group D)**								
Adesida *et al* [144]	Nigeria/ farm personnel (cow, sheep, goat, pig)	50/ 28 (56.0)/ 3 (6.0)	t084, t091, t1045, t127, t939, t311, t786, t1154/ t1045, 4 (14.3) and t091, 4 (14.3)	NT	NT	t786, 3 (100.0)	NT	NT
Fan *et al* [155]	China/ livestock workers	1162/ 108 (9.3)/ 16 (1.4)	NT	ST217, ST8, ST398, 3 (3.3), ST72, ST2339, ST15, ST1863, ST45, ST2238, ST43, ST95, ST2239, ST10, ST1, ST6, ST5, ST188, ST59, ST7, ST951, ST10 / ST7, 17 (18.5)	CC217, CC8, CC398, 3 (3.3), CC72, CC2339, CC15, CC5, CC45, CC2238, CC43, CC95, CC1917, CC10, CC1, CC6, CC188, CC59, CCT7, CC59, CC10 / CC7, 17 (18.5)	NT	ST5, ST188, ST398, 1 (6.3), ST59, ST7, ST951, ST10/ ST7, 7 (43.8)	CC5, CC188, CC59, CC7, CC59, CC398, 1 (6.3), CC10/ CC7, 7 (43.8)
Ye *et al* [156]	China/ Pig workers	244/ 37(15.2)/ 26 (10.7)	NT	ST7, ST943, ST6, ST188, ST15, ST45, ST88/ ST7, 5 (45.5)	CC7, CC6, C188, CC15, CC45, CC88/ CC7, 5 (45.5)	NT	ST 188, ST7, ST9/ ST9, 16 (43.2)	CC188, CC7, CC9/ CC9, 16 (43.2)
Etter *et al* [163]	Switzerland / livestock famers	160 (87 pupils and 73 members of farmer families)<br><br>73 (45.6)/ 2 (1.25) MRSA	NT	NT	In pupils:<br><br>CC6, CC12, CC152, CC188, CC5, CC7, CC8, CC59, CC9, CC398, 3 (7.9)/ CC45, 11 (12.6) and CC15, 10 (11.5)<br><br>In family members CC1, CC22, CC101, CC121, CC182, CC5, CC7, CC8, CC59, CC97, CC361, and CC509/ CC30 (n = 9) and CC45 (n = 5)	NT	NT	CC8 and CC30 in pupils
Bunke *et al* [172]	Germany/ pig farmers	86 / 54 (62.8)/ 18 (20.9)	t011, t034, t160, , t337, t012, t588, t065, t4755, t084, t16215/ t034, 4 (12.5)	NT	CC398, 5 (10.2)	t011, t034, t4652/ t011, 12 (70.6)	NT	CC398, 18 (100.0)
Mroczkowska *et al* [125]	Poland /pig farmers	283/ 50 (17.7)/ 4 (10.5) MRSA	t057, t108, 1 (2.2), t034, t4387, t2582, t1928, t1793, t318, t334, t2462, t1430/ t034, 14 (30.4)	NT	CC5, CC8, CC15, CC398, CC182/ CC398, 33 (71.7)	t034, t1430/ t034, 3 (75.0)	NT	CC30, CC398/ CC398, 3 (75.0)
Neyra *et al* [182]	USA/ hog plant workers	162/ 35 (21.6)/9 (5.6) MRSA	NT	19 unique STs<br><br>ST1, ST5, ST8, ST15, ST34, ST45, ST72, ST97, ST398, 3 (11.5),	NT	NT	ST8, ST1/ ST8, 4 (44.4)	NT

				ST508, ST1150/ ST8, 6 (23.1)				
Hatcher *et al* [183]	USA/ hog Operation Workers	198/105 (53.0)/ 32 (16.6) MRSA	t034, 2 (2.7), t189, t1077, t1937, t337, t688, t008, t015, t493, t2949, t185, t089, t002/ t688, 11 (15.1)	ST5, ST8, ST188, ST398, 2 (2.7), ST45, ST30, ST50/ ST5 12 (16.4)	CC9, CC398, 2 (2.7)/ CC9 (NR)	6 MRSA isolates t002, 2; t008, 2 and t088, 2	ST5, 2; ST8, 4	NR
Rinsky *et al* [184]	USA/ industrial livestock operation and antibiotic-free livestock operation workers (pigs)	99 versus 105/ 41 (41.0)/ 3 (3.0) versus 42 (40.0)/ 3 (2.9)	NT	ST1, ST5, ST8, ST9, ST30, ST45, ST182, ST188, ST2551, ST2546, ST97, and ST398, 10 (11.5) / ST30, 18 (21.7)	CC1, CC5, CC8, CC9, CC30, CC45, CC30, CC182, CC97, and CC398, 10 (11.5) / CC30, 18 (21.7)	NT	ST30, ST398/ ST398, 4 (66.7)	CC30, CC398/ CC398, 4 (66.7)
Okorie-Kanu *et al* [145]	Nigeria/ chicken and pig slaughterhouses workers.	45 each from chicken and pig slaughterhouses workers.<br><br>*S. aureus* (MSSA): 5 (11.1) versus 3 (6.7)	Pig carcass handlers: t311 and t279/ t311, 2 (66.7)<br><br>Chicken carcass handlers: t084, t311, t18346/ t084, 3 (60.0)	NT	Pig carcass handlers: CC5, CC15/ CC5, 2 (66.7)<br><br>Chicken carcass handlers: CC5, CC15/ CC15, 3 (60.0)	ND	ND	ND
Khanna *et al* [186]	Canada/ Pig farmers	25/ 5 (20.0) MRSA	NR	NR	NR	t1255, t034, t002/ t034, 3 (60.0)	NT	CC398, 3 (60.0)
Kittl *et al* [121]	Switzerland / animal farmers (pigs)	156/ 8 (5.1) MRSA	NR	NR	NR	t034, t1510, t899, 1 (12.5)/ t034, 5 (62.5)	ST22, ST45, ST398/ ST398, 6 (75.0)	CC398, 6 (75.0)
Mascaro et al [179]	Italy/ sheep farmers	275/ 3 (1.1) MRSA	NR	NR	NR	NT	NT	CC1, CC30, CC22
Graveland *et al* [180]	Netherlands/ veal Calf Farmers	97/ 32 (33.0) MRSA	NR	NR	NR	t002, t011, t015, t084, t108, t166, t899, t1457, t2383/ t011 (NR)	ST5, ST398, ST45, ST34/ ST398, 28 (87.5)	NT
Smith *et al* [185]	USA/ pig farm workers	148/31 (20.9) MRSA	NR	NR	NR	t002, t011, t021, t034, t084, t179, t330, t688/ t034, 20 (64.5)	ST398, 21 (67.7)	CC398, 21 (67.7)
Sahibzada *et al* [187]	Australia/ Pig farmers	52/ 31 (59.6) MRSA	NR	NR	NR	NT	ST93 and ST398, 5 (16.1)/ ST93, 26 (84.0)	NT
Moon *et al* [152]	Korea/ Pig farmers	126/ 21 (16.7) MRSA	NR	NR	NR	t034, t8588, t189, t324, t664/ t034, 10 (47.1)	ST541, ST398, 4 (19.0) ST541, ST188, ST72, ST72/ ST541, 6 (28.6)	CC398, 10 (47.1)
Cui *et al* [154]	China/ Pig workers	120/ 2 (1.7) MRSA	NR	NR	NT	t899 in both	ST9 in both	NT
Back *et al* [165]	Korea/ pig farm workers	186/ 6 (3.6) MRSA	NR	NR	NR	t18103, 1 (16.7) t034, 1 (16.7), t664, t5440/ t664, 2 (33.3) and t5440, 2 (33.3)	ST2084, ST541, 1 (16.7), ST398, 1 (16.7)/ ST2084, 4 (66.7)	CC398 2, (16.7)
Dahms *et al* [174]	Germany/ pig farmers	78/ 20 (25.6) MRSA	NR	NR	NR	t011, t034, t1451, t2370, t10721, t3275 / t034, 6 (30.0)	NT	All were CC398
Reynaga *et al* [181]	*Spai*n/ pig farm workers	140/ 81 (57.9) MRSA	NR	NR	NR	t011, t034, t1456, t1451, t108, t943, t1197, t2346/ t011, 54 (67.9)	ST398 in all isolates	NT

Normanno *et al* [161]	Italy/ pigs and abattoir workers	113/ 9 (7.9) MRSA	NR	NR	NR	NT	ST1, ST2794, ST8, ST15, ST398/ ST1, 3 (33.3) and ST398, 3 (33.3)	CC1, CC8, CC15, CC398/ CC1, 3 (33.3) and CC398, 3 (33.3)
Sinlapasorn *et al* [188]	Thailand/ Pig farmers	98 / 3 (3.1) MRSA	NR	NR	NR	NT	ST764, ST9/ ST9, 2 (66.7)	NT
Kock *et al* [175]	Germany/ pig farmers	35/ 27 (77.1) MRSA	NR	NR	NR	t011, t034, t1451, t1197 / t011, 16 (59.3)	NT	All were CC398
Parisi *et al* [167]	Italy/ pig farm worker	150/NR/ 26 (17.5)	NR	NR	NR	t011,6 (23.1), t034, t1730, t899, 6 (23.1), t1939, t002/ t034, 10 (38.5)	ST398, ST79, ST9, ST1, ST5/ ST398, 22 (84.6)	CC9, 1 (3.8), CC398, 22 (84.6)
Papadopoulos *et al* [166]	Greece/ Dairy workers	26/10 (38.5) /2 (7.7)	NT	NR	NR	t021, t127	NT	NT
Kalayu *et al* [148]	Ethiopia/ Dairy farmers	71/22 (31.0)/ 1 (1.4)	NT	NT	NT	t064	NR	CC8
Sakwinska *et al* [168]	Switzerland /Bovine farmers	158/57 (36)/ 2 (1.3)	NT	NT	NT	t011, 2 (100)	ST398, 2 (100)	NT
VAN CLEEF *et al* [164]	Netherlands/ pig slaughterhouse workers	249/ NR/14 (5.6)	NR	NR	NR	t011, t034 1 (7.1), and t1451, 1 (7.1), t108, 1 (7.1) / t011, 11 (78.6)	NT	All were CC398
Odetokun *et al* [146]	Nigeria/ Pig workers	260/35 (13.5)/ 8 (3.1)	NR	NR	NR	t091, t1937, t816, t4235, t16571/ t091, 3 (37.5)	NT	NT
Otalu *et al* [147]	Nigeria/ Pig workers	55/NR/ 6 (10.9)	NR	NR	NR	t1603, 8 (100.0)	ST88	NT

Supplementary Table S7. Study characteristics, species, and antimicrobial resistance profile of non-*aureus* staphylococci nasal carriage in healthy people with or without risk of colonization (Groups A-D).

Author (s) (Reference)	Staphylococci group	Continent	Country/Subjects	Study design	Sample size	No. of staphylococcal positive cases (%)	Detection method	*Staphylococcus* species (non-*aureus*) detected (%)[b]	Antimicrobial Resistance phenotype Technique/ Major resistant types[c]	Antimicrobial Resistance genes

Abadi *et al* [82]	CoPS and CoNS	Asia	Iran/Healthy school students	Cross-sectional	600	539 (89.8)	C / PCR	SL (29), SHae(24) SSa(23.6), SE (12.5), SSli(11.1)<br><br>MR-isolates:<br>72 (16.7) MRCoNS were identified.<br>SSa had the highest MR rate.	DD<br><br>FOX	*mecA*
Lebeaux *et al* [85]	CoNS	Europe	France /Healthy community	Cross-sectional	154	72 (46.7)	C / PCR / MT	SE (21.4), SHae(27), SH (18.3), SSa(16.2), others(3)	NT	ND
Falomir *et al* [83]	CoPS and CoNS	Europe	Spain/ Healthy older persons (average 63.7 years)	Cross-sectional	27	4 (15.0)	C	Swa<br>Spa, Skl,<br>Sxy	DD<br><br>(CoNS)<br>PEN, OXA, TET, CLA, RIF, CIP	NT
Falomir *et al* [84]	CoPS and CoNS	Europe	Spain/ Healthy university students	Cross-sectional	445	99 (22.2)	C	Sin, Ssa, Skl, Swa, and Sca	DD<br><br>OXA, ERY, MUP	NT
Aung *et al* [115]	CoPS	Asia	Burma/ food handlers	Cross-sectional	563	110 (19.5)	C/ PCR/MT	*Sarg*, 1 (0.8)	NT	NR
Boost *et al* [139]	CoP	Asia	Hong Kong/ Veterinary personnel	150	Cross-sectional	NR	C/PCR/MT	Sps 2 (1.3)	NT	*mecA*
Malissiova *et al* [140]	CoNS	Europe	Greece/ Healthy veterinary students	81	Prospective	22 (27.2)	C / PCR / MALDI	SHae(54), SW(45.8), SE (16.6), SPas (4.2) SCa (4.2).	DD<br><br>FOX	*mecA (S. epidermidis)*
Paterson *et al* [136]	CoPS and CoNS	Europe	UK/ cattle veterinary practitioners	307	Cross-sectional	NR	C/PCR/MT	*SHae*	MIC<br><br>OXA, FOX, ERY, CLI, TET, SXT	*tetM tetL aacA, aphD, dfrK ermC ermT, mecA*

Paul *et al* [138]	CoPS	Europe	Denmark/ small animal veterinarian	128	Prospective	NR	C/PCR	MRSP, 5 (3.9)	DD FOX	*mecA*
Neradova *et al* [135]	CoPS and CoNS	Europe	Czech Republic/ Veterinary personnel	134	Cross-sectional	40 (29.9)	C/PCR/MT	Sps, 2 (1.5)	DD FOX, CXT, TET, GEN, CIP	*mecA*
Founou *et al* [189]	CoNS	Africa	Cameroon and South Africa/ Pig workers	53 in Cameroon and 24 in South Africa	Comparative prospective	ND	C/PCR	50 (94,3) and 7 (29.2) MRS in Cameroon and South Africa receptively. *Sep, SHae, SL, Slut, SH*	DD PEN, OXA, FOX, TET, SXT	*mecA*
Sinlapasorn *et al* [188]	CoPS and CoNS	Asia	Thailand/ Pig farmers	98	Cross-sectional	3 (3.1)	C/PCR/MT	SSc,Shae, *S cohnii, subspecies urealyticus, S. hominis subspecies hominis*, SChr, SE	MIC OXA, FOX, GEN, ERY, TET, OF	*mecA*
Rall *et al* [113]	CoPS and CoNS	America	Brazil/ Food handers	62	Cross-sectional	15 (22.1)	C/PCR	S.ep, Sw	NT	NT
Argudın *et al* [190]	CoNS	Europe	Belgium/ farmers	86	Cross-sectional	NR	C/PCR/MT	Sep, 64 (75) MRSE, 44 (51.1)	DD PEN, FOX, CLI, KAN, TET, ERY, SXT	*mecA*
Roberts *et al* [191]	CoPS and CoNS	America	USA/ healthy dairy farmers	24	Cross-sectional	5 (20.8)	C/PCR	Sep, Shae, Shom, Ssci, Sxy	NT	NT
Lim *et al* [158]	CoPS and CoNS	Asia	Korea/ Dairy farm workers	43	Cross-sectional	NR/ MRSA, 1 (2.3)	C/PCR/MT	MRCoNS, 1 (2.3)	*mecA*	NT

[a]C, culture; MALDI, MALDI-TOF-MS; PCR, PCR; MT, molecular typing.

[b] SE*: S. epidermidis;* SHae*: S. haemolyticus;* SSa*: S. saprophyticus;* SL*: S. lugdunensis*; SCa: *S. capitis*; SC: *S. cohini:* SH: *S. hominis*; SHy: *S. hyicus;* SCs: *S. caseolyticus;* SSi: *S. simulans*; SD; *S. delphini;* SSli: S. *schleiferi* SW: *S. warnieri*; SPas: *S. pasteuri. S. pseudintermedius*; Sps; *Schr: S. chromogenes; SSci: S. sciuri: Sin: S. intermedius;* MRSP (methicillin-resistant *S. pseudintermedius*)

[c]Technique used: Disk-diffusion method (DD-method), MIC determination (MIC). Top 4 groups of antimicrobial agents: beta-lactams (BL). Fluoroquinolones (FQ), Macrolides (Mc), Tetracycline (Tet), Vancomycin (Van)

[d]NT: Not tested

NA: Not available (tested but result not published on article)

ND: Not detected

NR: Not reported in detail

PEN: Penicillin; AMX: Amoxicillin; ERY: Erythromycin; CHL: Chloramphenicol; CLI: Clindamycin; STR: Streptomycin; Van: vancomycin; TET: Tetracycline; OXA: Oxacillin; RIF: Rifampicin, FOX: Cefoxitin; LEV: Levofloxacin; CIP: Ciprofloxacin; KAN: Kanamycin; GEN; Gentamycin; MUP: Mupirocin; SXT: Trimethoprim/sulfamethoxazole.
